# High-throughput spatial proteomics from cellular to subcellular scales via a hanging droplet workflow

**DOI:** 10.1038/s41467-026-78415-x

**Published:** 2026-10-09

**Authors:** Hao Chen, Ulises H. Guzman, Jesper V. Olsen

**Affiliations:** 1 Novo Nordisk Foundation Center for Protein Research, Department of Cellular and Molecular Medicine, Faculty of Health and Medical Sciences, University of Copenhagen, Copenhagen, Denmark; 2 Copenhagen Center for Glycocalyx Research, Department of Cellular and Molecular Medicine, Faculty of Health and Medical Sciences, University of Copenhagen, Copenhagen, Denmark

**Keywords:** Mass spectrometry, Proteomic analysis, Proteomics

## Abstract

Formalin-fixed paraffin-embedded (FFPE) samples are central for clinical research and spatial proteomics (SP), yet liquid chromatography-mass spectrometry (LC-MS) remains challenging due to protein extraction losses, evaporation during decrosslinking, and inefficient sample preparation. Current methods often involve complex protocols or specialized equipment, limiting scalability, throughput, and proteome depth. We present a streamlined micro-FFPE proteomics protocol for laser-capture microdissection (LMD) that enables high-throughput processing in a 96-well format, completing the workflow from tissue lysis to LC-MS-ready tryptic peptides on Evotips in just 2-hours. By integrating whole-slide imaging, LMD, and narrow-window data-independent acquisition mass spectrometry (nDIA-MS), our approach optimizes lysis, decrosslinking, and digestion within nano-wells leveraging the hanging droplet concept. This design minimizes surface adsorption, enhances sample processing efficiency, reduces preparation time by ~30% compared with reference methods, and enables parallel handling of up to 192 samples with standard equipment. This enables the quantification of >5,500 protein groups (PGs) from 50,000 µm² regions (~250 cells), with robust performance down to 1000 µm2 (~5 cells) with ~2,500 PGs identified. Application to breast cancer (BC) tissue resolves BC subtypes and microenvironments via biomarkers (TP53, HER2, PR). Even from ~60 µm² regions of single nuclei identify ~1,800 protein groups, enabling microscale spatial proteomics approaching near-organelle resolution.

## Abstract (web summary)

Formalin-fixed paraffin-embedded (FFPE) samples are central for clinical research and spatial proteomics. Here, the authors present a streamlined micro-FFPE proteomics workflow that enables rapid, high-throughput spatial proteomics of laser-capture microdissected tissue.

## Introduction

Eukaryotic cells in tissues and organs are embedded in highly organized tissue architectures that facilitate specialized biological functions^1^. The identity and function of cells are ultimately defined by their individual proteomes; however, it is equally important to consider the spatial distribution of these proteins within tissues and subcellular compartments. This spatial context is crucial for interpreting how proximity to neighboring cells, access to signaling cues, and positioning within multicellular networks influence cellular function. The emerging field of spatial proteomics addresses this need by capturing protein abundance differences within intact tissue architecture, moving beyond the averaged proteome profiles obtained from bulk and dissociated single-cell analysis. For example, Deep Visual Proteomics (DVP) is a cutting-edge technology that integrates high-resolution imaging, artificial intelligence-driven cellular phenotype analysis, laser-capture microdissection, and ultra-sensitive mass spectrometry to map thousands of proteins at single-cell resolution while preserving spatial context. By linking proteomic information to precise morphological features at cellular and subcellular resolution, spatial proteomics enables the identification of cellular heterogeneity and dynamic intercellular communication that would otherwise remain hidden in complex environments, such as the tumor microenvironment (TME) or in intricate neuronal signaling networks^2,3^.

A uniquely enabling resource for spatial proteomics is the vast number of global archives of Formalin-Fixed Paraffin-Embedded (FFPE) human tissue blocks^4^. These worldwide archived samples, preserved as part of routine clinical practices, retain fine morphological structure and are often paired with well-annotated clinical histories. These samples represent an unparalleled biobank of human biology across disease stages, tissue types, and demographic backgrounds. The chemical crosslinking used for preservation is essential for maintaining tissue architecture and enabling precise pathological annotation and LMD, but it also makes protein extraction challenging^5^. Therefore, developing robust sample preparation methods to recover high-quality data from FFPE samples is critical for translating spatial proteomics into widespread clinical and translational research applications^6^.

A central bottleneck in spatial proteomics workflows lies in reversing the formaldehyde-induced covalent methylene bridges between proteins, a process that requires high temperatures (typically >90 °C) to restore protein accessibility for enzymatic digestion^7^. While effective for bulk samples, high-temperature decrosslinking is particularly challenging at the microscale or in thin tissue sections, where the small reaction volumes are highly susceptible to rapid evaporation before decrosslinking is complete. As drying occurs, three major issues arise. First, the tissue can be physically lost or damaged, directly reducing proteome coverage. Second, premature drying interrupts the *de*crosslinking process and prevents efficient enzymatic digestion, leading to severely reduced peptide recovery and failure of the overall sample preparation workflow^8^. Third, as the evaporation is difficult to control, it is often variable across FFPE samples, leading to loss of sample preparation reproducibility.

To mitigate these challenges, some approaches employ continuous replacement of buffer within a heating system^9,10^, enabling robust proteome coverage and high throughput. However, these methods rely on specialized instrumentation^9,10^, which limits their scalability and broader applicability. Other workflows depend on multi-step processing, including SpeedVac drying^3,11,12^, repeated *de*crosslinking cycles^3,11^, and prolonged tryptic digestion^3,11,12^, all of which reduce throughput. More recently, innovative “hanging droplet” strategies have been developed to mitigate evaporation in a simpler and more practical manner. For instance, a “hang-down” droplet configuration has been implemented to enhance *de*crosslinking and peptide generation from microscale samples by minimizing evaporation during heating^13^. Similarly, the Surfactant-assisted One-Pot voxel method (wcSOP) employs a suspended droplet on the tube cap to process laser-capture microdissected material, improving sample retention and proteomic depth^14^. However, despite their effectiveness in preventing evaporation and enhancing sensitivity, these approaches remain inherently low-throughput, as they require processing samples individually.

Here, we introduce a one-pot, high-throughput spatial proteomics workflow capable of processing hundreds of LMD tissue regions in parallel. Our method leverages the proteoCHIP EVO 96 chip to directly collect samples from a laser-capture microdissection microscope system, employing a suspended droplet format to prevent evaporation during thermal *de*crosslinking while maintaining stable reaction volumes, and reduces sample preparation time by ~30% compared with reference methods^10^. This approach enables robust proteome profiling from extremely limited starting material, identifying ~5500 protein groups (PGs) from FFPE sample regions containing ~ 250 cells and ~ 2500 PGs from regions containing ~5 cells using an 80 samples-per-day (SPD) LC-MS workflow. Comparable performance is retained under rapid-gradient conditions (120 SPD), covering ~90% of the proteome depth achieved at 80 SPD in 4000 µm² BC sections. Moreover, our method achieves subcellular resolution, yielding 1800 PG-form subcellular structures as small as ~60 µm² (e.g., isolated nuclei). Finally, we demonstrate the applicability of our method by profiling breast cancer tissues from four distinct molecular subtypes, accurately resolving tumor-and microenvironment-specific proteomic signatures.

## Results

### High-throughput scalable workflow optimization

**Fig. 1:**
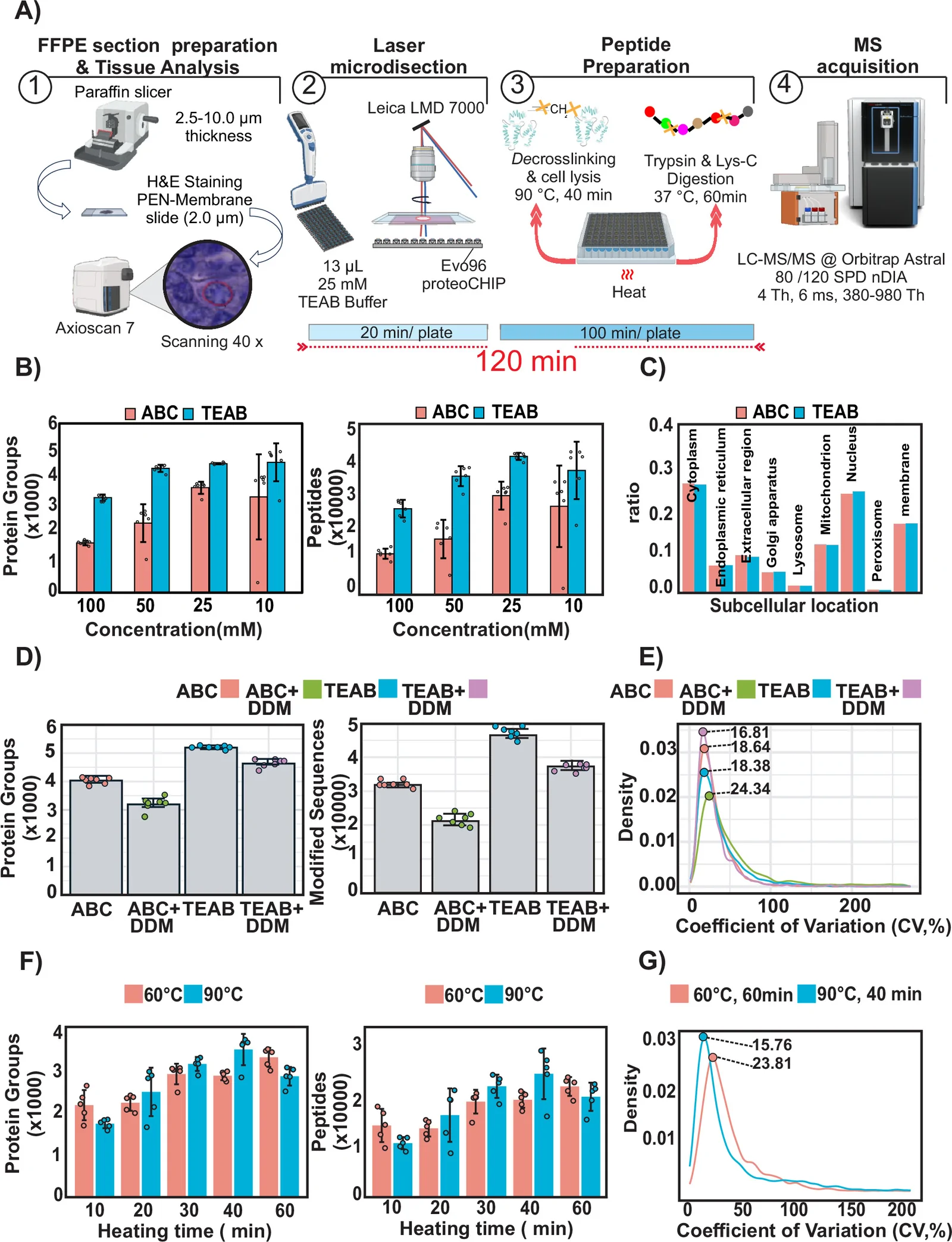
High-throughput hanging droplet workflow for micro-FFPE spatial proteomics. **A** Schematic overview of the integrated workflow combining whole-slide imaging, laser capture microdissection, and suspended droplet sample processing on EVO96 chips. **B** Comparison of Protein Groups and Modified Sequences identified using Ammonium Bicarbonate (ABC) versus Triethylammonium Bicarbonate (TEAB) digestion buffers across different concentrations (*n* = 6, biological replicates). **C** Proportion of subcellular localization categories among proteins identified under optimal ABC and TEAB conditions (25 mM). **D** Comparison of Protein Groups and Modified Sequences identified using Ammonium Bicarbonate (ABC) versus Triethylammonium Bicarbonate (TEAB) digestion buffers with or without n-Dodecyl-β-D-maltoside (DDM). (*n* = 7, biological replicates) **E** Distribution of coefficients of variation (CV) across different buffer systems used during the digestion process. Colored points indicate the median CV for each condition. **F** Optimization of decrosslinking temperature (Blue: 90 °C, Red: 60 °C). (*n* = 5, biological replicates) **G** Distribution of Coefficients of Variation (CV) across different temperatures used in the decrosslinking process. Colored points indicate the median CV for each condition. Data are presented as mean ± SD. Figure 1A was partially created in Biorender.com. Olsen, J. (2026) https://BioRender.com/g8xhcyq. The MS acquisition section was created by the authors based on publicly available information.

Spatial proteomics (SP) faces several technical challenges that can limit its effectiveness, particularly during sample preparation. One major issue is sample loss during protein extraction, which can adversely affect both the quantity and quality of the resulting data. Additionally, evaporation during the *de*crosslinking process threatens sample integrity, complicating subsequent analyses. Current sample preparation methods often rely on specialized equipment or multi-step processing, creating barriers to broader adoption. To address these limitations, streamlined sample preparation strategies are required that minimize sample loss and evaporation while improving throughput. Here, we developed a formalin-fixed paraffin-embedded (FFPE) sample preparation protocol that integrates the hanging droplet concept^13,15^. This approach minimizes sample loss, enhances *de*crosslinking efficiency, and circumvents the need for specialized equipment during sample preparation, as the sample is directly excised from the FFPE samples into a reaction chamber formed by a droplet on Teflon-based capillaries of EVO96 proteoCHIP (Fig. 1A, Supplementary Fig. 1A, Table 1.). Briefly, the hanging droplet concept utilizes droplets that remain suspended from the chip pillars when the EVO96 proteoCHIP is placed face-down over a standard 96-well plate and heated to 90 °C. In this system, droplet stability is governed by the interplay of surface tension and gravity, while evaporation during *de*crosslinking and digestion is minimized by vapor-liquid equilibrium within the confined headspace. This configuration enables rapid saturation of the surrounding headspace environment with solvent vapor, effectively suppressing net evaporation and maintaining stable reaction volumes throughout the *de*crosslinking process and digestion, with minimal volume loss (~1 µL; Supplementary Fig. 1A).

**Table 1:** General reagents and tools

Reagent or tools	Company	Identifier/catalog/verison
Reagent		
Triethylammonium bicarbonate buffer (TEAB)	Sigma	18597
n-dodecyl-β-D-maltoside (DDM)	Sigma	850520 P
Formic Acid, LC-MS Grade	Thermor Fisher	28905
ddH2O	MilliQ	NA
Trypsin	Promega	V5280
Lys-C	Thermo Fisher Scientific	A40007
Acetonitrile	Merck	1000302500
2-proponal	Merck	1070222511
Tools		
1.5 mL PCR Tube	Eppendorf	0030120086
Evotips	Evosep	EV2014
EVO 96 chips	Cellenion	C-PEVO-96-10
Adapter for EVO 96 chips	-	NA
3D printer	UltiMaker	S3
Heating Block Armadillo plate	Eppendorf Theromo Fisher	38-5360000038 AB-3396
AxioScan	ZEISS	7
Laer microdissection	Leica Microsystems	7000
Evosep, Eno	Evosep	EV1500
Column	Aurora Rapid, ionopticks	4 generation
Orbitrap Astral	Theromo Fisher	NA
Software		
Leica LMD Software	Leica Microsystems	8.0.0.6043
ZEISS ZEN	ZEISS	3.9
Orbitrap Astral application	Thermo Fisher	1.0.100.40
Chronos	Axel Semrau	4.10.0.0
Spectronaut	Biognosys	20
R	RStudio Desktop - Posit	4.4.2
R.studio	RStudio Desktop - Posit	2025.5.0.496
Adobe Illustrator	Adobe	2019
Qupath	www.qupathgithub.io	0.5.0
Qupath_to_LMD	https://github.com/CosciaLab/Qupath_to_LMD	NA

To establish a high-throughput and scalable spatial-proteomics workflow, we next defined optimal buffer conditions for Lys-C and trypsin proteolytic digestion within the hanging droplet by comparing two commonly used MS-compatible digestion buffering systems: Ammonium Bicarbonate Buffer (ABC) and Triethylammonium Bicarbonate (TEAB), each tested across a range of concentrations from 0 to 100 mM applied to 20,000 µm² BC sections (5 µm thickness). Notably, TEAB demonstrated better performance compared to ABC, yielding approximately 12.5% more PGs and 25% more peptide identifications at the optimal buffer concentration of 25 mM (Fig. 1B).

This improved performance of TEAB may reflect subtle differences in buffering behavior and physicochemical properties in microscale droplets. Although both salts provide comparable pH buffering capacity in solution, TEAB is less volatile than ABC and therefore may maintain more stable local ionic conditions during extended incubation in small-volume droplets.

The observation that the optimal buffer concentration for both systems is around 25 mM emphasizes the importance of balancing buffer concentration to maintain pH stability within the hanging droplet for effective tryptic digestion, while ensuring a low salt concentration to minimize disturbances in vapor pressure and surface tension within the system, as such disturbances lead to reduced identifications or increased variability in the results. Regardless of the buffer system used at optimal buffer concentration, both systems identified similar subcellular terms, suggesting no apparent protein identification bias (Fig. 1C).

Next, we tested the effect of adding the MS-compatible surfactant n-Dodecyl β-D-maltoside (DDM) to LMD-excised micro-FFPE BC samples (20,000 µm², 10 µm thickness) prepared in ABC and TEAB buffer systems. Although DDM is commonly used to reduce protein adsorption to plastic surfaces, a major source of loss when handling minute specimens such as single cells and micro-FFPE material, its omission in both buffer conditions resulted in higher numbers of identified PGs and modified peptide sequences. Among the tested buffers, TEAB consistently demonstrated better performance, yielding greater protein and peptide recovery in combination with improved reproducibility, as reflected by lower coefficients of variation (CVs) (Fig. 1D,E). The same trend was observed whether DDM was added before (B) or after (A) the decrosslinking step (Supplementary Fig. 1B,C). Furthermore, to test this effect in greater detail, we evaluated a DDM concentration range from 0 to 0.2 percent in the optimal 25 mM TEAB digestion buffer using BC samples (20,000 µm² BC sections; 5 µm thickness). Reassuringly, we observed a consistent decrease in identified PGs and peptides with increasing DDM concentration (Supplementary Fig. 1D). Given that DDM is also known to enhance solubilization of membrane-associated proteins, we compared the subcellular distribution of identified proteins across conditions (Supplementary Fig. 1E). Although a slightly higher proportion of membrane proteins and proteins associated with extracellular regions was detected when 0.02% DDM was added to the digestion buffer, the overall number of identifications remained consistently higher when using TEAB alone. This behavior likely reflects competing effects of the detergent: while DDM can reduce sample loss by limiting surface adsorption and improve stabilization of membrane proteins, it also alters droplet surface tension, which may compromise droplet stability during heating (Supplementary Fig. 1F).

To further optimize the workflow, we systematically varied the concentration of the Trypsin/Lys-C mix (1 to 25 ng/µL), the proteolytic digestion time (15–90 min), the *de*crosslinking time (10–60 min), and temperature (60 and 90 °C). We found that a protease mix concentration of 5 ng/µL was optimal, at which both the mean number of identified PGs and peptides reached a maximum, while further increases in protease concentration did not improve identification rates on minute samples (20,000 μm², 10 µm-thick BC sections; Table 2) (Supplementary Fig. 2A). A similar trend was observed for the missed tryptic cleavage rate, where the proportion of peptides with zero missed cleavages increased only marginally (by approximately 1–3%), even when the protease mix concentration was increased 2- to 5-fold (Supplementary Fig. 2B). Evaluating the missed cleavage rate is a critical metric for assessing the efficiency of proteases in proteomics, particularly for trypsin, where lower rates indicate higher enzyme efficiency. Moreover, a 60 min digestion time resulted in the highest mean number of identified PGs and modified peptide sequences, while maintaining a similar proportion of missed cleavages compared to a 90 min digestion (Supplementary Fig. 2C, D). For the *de*crosslinking step, a 40-minute incubation yielded the greatest number of identified protein groups and modified peptides. Additionally, adjusting the *de*crosslinking temperature to 90 °C improved both protein and peptide recovery while also enhancing reproducibility across replicates (Fig. 1F, G). Together, these optimized conditions enable the preparation of LC-MS-ready samples in a 96-well format in approximately 2 h.

**Table 2:** Research materials

Series	Organ	Pathology Diagnosis	Gender	Age	Grade	TMN	Thickness	IHC
02005 T	Human Breast	Human breast Invasive ductal carcinoma	Female	50	2	Na	5/10 um	ER(+), PR(+), P53(-), Her2 (+)
02004 T	Human Breast	Human breast Invasive ductal carcinoma	Female	50	1	Na	5 um	ER(+), PR(+),P53(-), Her2 (-)
02002 T	Human Breast	Human breast Invasive ductal carcinoma	Female	37	3	Na	5 um	ER(-), PR(-),P53(-), Her2 (+)
02024 T	Human Breast	Human breast Invasive ductal carcinoma	Female	65	3	Na	5 um	ER(-), PR(-),P53(-), Her2 (-)

Finally, we compared on-chip hanging droplet digestion using the EVO 96 proteoCHIP with on-tip digestion performed directly on the C18 resin membrane inside an EvoTip, following transfer of the droplet from the proteoCHIP by centrifugation. Importantly, both conditions were carried out using the same input material and identical samples to ensure a fair comparison (FFPE BC samples 20,000 µm², 10 µm thickness). Encouragingly, we found that the hanging droplet approach enables the identification of twice as many protein groups and more than twice as many modified peptide sequences compared to on-tip digestion, while also showing a lower proportion of missed-cleaved tryptic peptides compared to the fastest published workflow to date using comparable digestion conditions^10^ (Supplementary Fig. 2E, F).

### High-throughput deep proteome coverage of FFPE samples in single-shot analysis across variable sample loads

Because the objective of our method is to enable robust, high-throughput processing of more than 100 SPD from sample preparation through mass spectrometry analysis, we stress-tested the sample collection step of the workflow. This step represents a known limitation in laser microdissection (LMD)–based workflows, where sample transfer from the LMD collection surface into the reaction chamber can compromise robustness and throughput^14,16^. We therefore optimized the liquid volume required during collection and evaluated the effect of adding buffer prior to collection versus collecting without pre-added buffer across different sample sizes. Under standard laboratory conditions, we measured an evaporation rate of 0.13 µL min⁻¹ from an initial volume of 15 µL TEAB buffer on the chip pillar. This rate permits sample collection in a 96-well plate format for approximately 40 min while retaining sufficient buffer volume to support subsequent *de*crosslinking and proteolytic digestion using the hanging-droplet approach (Supplementary Fig. 3A). Although the collection rate depends on tissue area and the LMD cutting parameters, we found that 96 samples can typically be collected within 20–30 min under standard LMD settings. We next assessed the impact of adding buffer prior to collection versus collecting without pre-added buffer across different sample sizes. A marginal improvement in collection efficiency (~6%) was observed only for very small samples 20,000 μm², 10 µm-thick, BC sections) when buffer was added prior to collection (Supplementary Fig. 3B). However, when sections equivalent to 5- and 20-cell samples were analyzed, the number of identified protein groups (PGs) and peptides was comparable between conditions, with high reproducibility regardless of whether TEAB buffer was added. This was further supported by principal component analysis (PCA), which showed no separation between conditions in the score space, but consistent clustering patterns in the loading profiles (Supplementary Fig. 3C–F). Together, these results indicate that the presence of buffer prior to collection does not affect proteome depth, while modestly improving collection efficiency for minute samples, an effect that may become more pronounced when ultra-low input samples are analyzed.

**Fig. 2:**
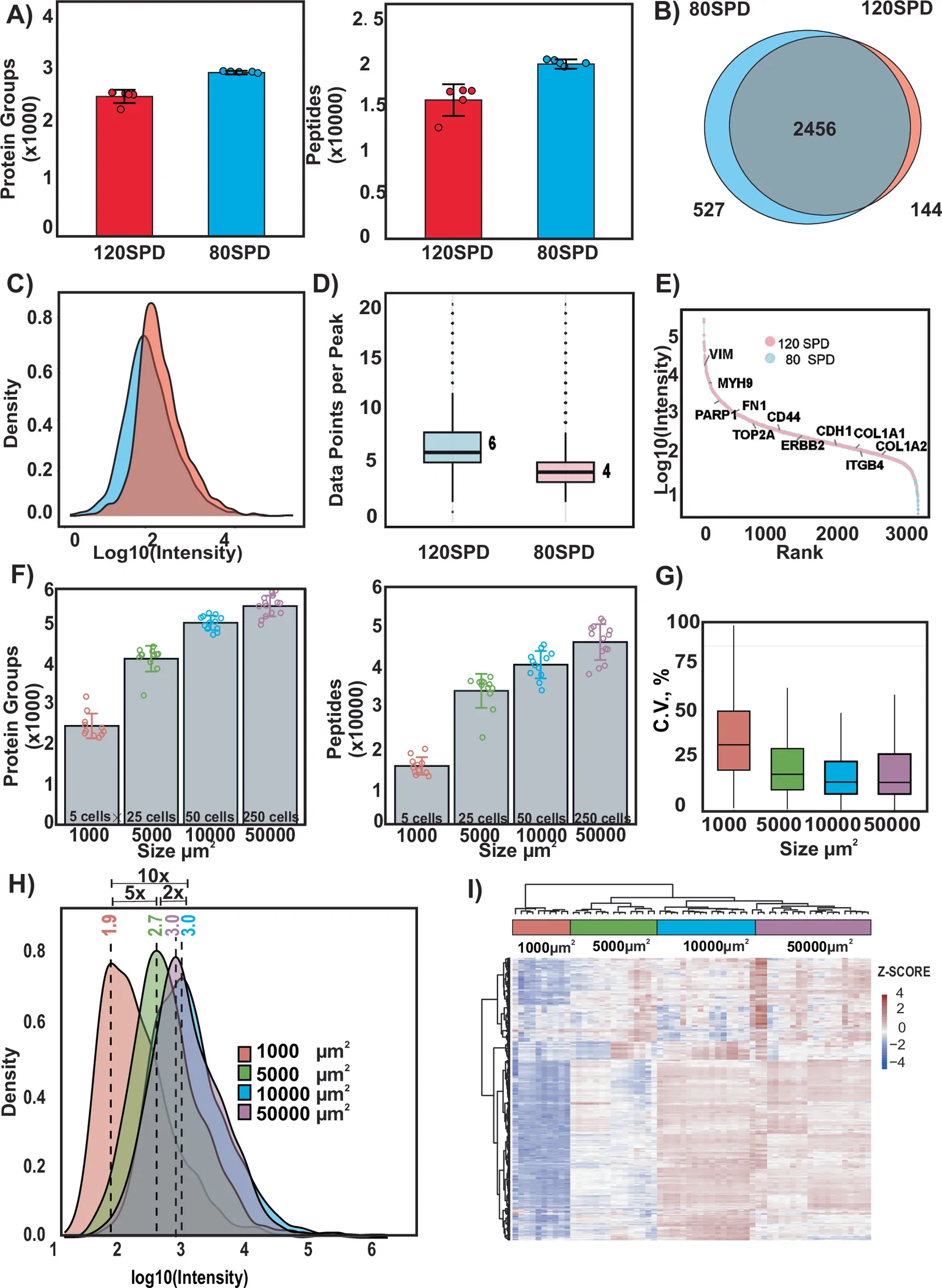
Single-shot proteome coverage across microdissected FFPE inputs and short LC gradients. **A** Protein groups and peptides identified at 80 samples-per-day (SPD) versus 120 SPD LC gradients on the Evosep One system (*n* = 5, biological replicates). **B** Venn diagram showing the overlap of protein groups identified between both LC gradients (80 SPD and 120 SPD, *n* = 5, biological replicates). **C** Density plot of protein groups intensities for the 80 SPD and 120 SPD methods (red and blue, respectively). **D** Box plot comparing the distribution of data points per peak for the 80 SPD and 120 SPD LC gradients (*n* = 5, biological replicates). **E** Comparison of the dynamic range of identified protein groups between the 80 SPD and 120 SPD LC gradients; commonly identified protein groups are highlighted. **F** Protein group and modified sequence identifications across different microdissected region size inputs, ranging from ~50,000 μm² (≈250 cells) to ~1000 μm² (≈5 cells) (*n* = 12, biological replicates). **G** Box plots denoting the coefficient of variation (CV) across different microdissected region size inputs (*n* = 12, biological replicates). **H** Distribution of log10-transformed intensities of common protein groups across different microdissected region input sizes. Fold changes between conditions are indicated above. **I** Heatmap displaying unsupervised hierarchical clustering across different microdissected region size inputs. All box plots include the median line, the box denotes the interquartile range (IQR), whiskers denote the rest of the data distribution and outliers are denoted by points greater than ±1.5 × IQR. Data are presented as mean ± SD.

Once we established the robustness of the sample collection workflow, we evaluated our FFPE sample preparation approach using rapid Whisper Zoom liquid chromatography (LC) gradients on the Evosep One system (80 and 120 SPD), coupled via an electrospray source to an Orbitrap Astral mass spectrometer. This configuration employed a narrow-window data-independent acquisition (nDIA) method^17,18^, which has demonstrated the sensitivity required for analysis of 10-cell–level sample amounts (4000 μm², 5 μm). As expected, the 80 SPD method identified ~15% more PGs and ~25% peptides than the 120SPD method (Fig. 2A). Nevertheless, both methods shared ~80% of the identified PGs (Fig. 2B), enabling coverage of similar top ten Gene Ontology (GO) biological process terms (Supplementary Fig. 4A). This indicates that, although the 120 SPD method moderately reduces overall proteome coverage, it retains the ability to capture comparable biological signatures to the 80 SPD method (Supplementary Fig. 4A). Closer inspection of the log₁₀-transformed protein intensity distributions showed that the 80 SPD method enabled identification of lower-intensity protein groups (PGs) and provided higher quantitative performance with a higher number of data points per peak (6 DPPP for 80 SPD versus 4 DPPP for 120 SPD) (Fig. 2C, D). Despite this difference, the 120 SPD method still identified key PGs relevant to BC biology, including PARP1, TOP2A, ERBB2, CDH1, and COL1A1, all of which fell within the three highest intensity quantiles of the log₁₀-transformed intensity distribution in both methods (Fig. 2E).

To place our method in context with current state-of-the-art standardized LMD-based spatial proteomics workflows, such as scDVP^11^ and openDVP^10^, we benchmarked our workflow against published datasets. Although the sampled tissues differed, this comparison enabled evaluation of performance at minute sample inputs and under rapid LC gradient conditions.

Encouragingly, our method identified 27% more PGs and 38% more peptides than openDVP for samples with same area and thickness (8000 µm², 5 µm) using the 80 SPD gradient (Supplementary Fig. 4B). Despite differences in tissue type, this comparison highlights the identification performance of our method at comparable input amount and gradient length. Furthermore, when compared with scDVP at similar single-cell equivalent inputs based on volume (2000 µm³), our approach identified ~46% more peptides while achieving a comparable number of PGs (Supplementary Fig. 4C). Although scDVP identified slightly more low-intensity PGs, this is likely attributable to its longer online LC gradient (40 SPD) and the use of a single compensation voltage (CV) FAIMS acquisition strategy. Overall, the hanging-droplet methods represent a favorable trade-off between analytical throughput and proteome depth for low-input samples, providing substantially higher peptide identifications while maintaining comparable PG coverage in half the analysis time (Supplementary Fig. 4D).

As a final step, we evaluated the proteome coverage achieved from BC FFPE samples processed using the hanging-droplet method across excised areas ranging from 1000 µm² to 50,000 µm² at a thickness of 10 µm, corresponding to approximately 5 to 250 cells equivalents using the 80 SPD gradient (Supplementary Fig. 5A). As expected, the number of identified PGs and modified peptide sequences scaled with input amount, identifying approximately ~5500 PGs and ~50,000 modified peptides at the highest input (~250 single cells), and ~2500 PG and ~13,000 modified peptides at the lowest input (~5 single cells), representing 42% of the proteins detected at the highest load (Fig. 2F and Supplementary Fig. 5B). Notably, smaller sampled regions exhibited greater biological heterogeneity, resulting in reduced reproducibility (Fig. 2G). Nonetheless, low-input samples showed ~85% overlap in identified PGs with Pearson correlation coefficients of ~ 0.9 in their quantitative profiles, indicating high reproducibility and consistency even at minimal input levels (Supplementary Fig. 5C, D). While larger samples exhibited higher overall protein intensity, signal intensity scaled with input as expected and reached saturation at loads exceeding ~50 cells (Fig. 2H, I). Gene Ontology (GO) term enrichment analysis further showed that, despite fewer identifications in low-input regions, the core functional processes remained consistent (Supplementary Fig. 5E, F).

### Spatial characterization of the tumor microenvironment within triple-negative breast cancer (TNBC) tissue samples

**Fig. 3:**
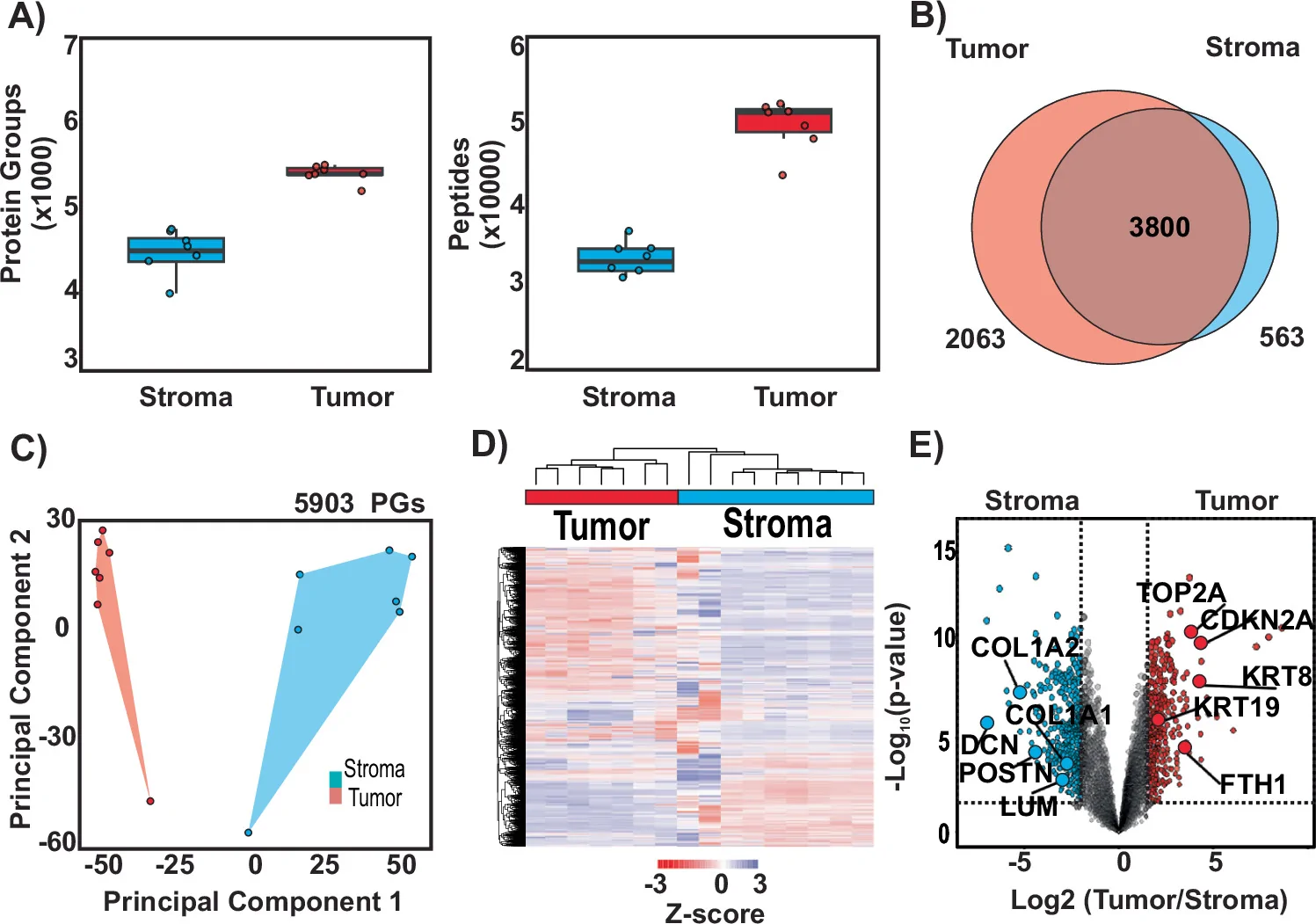
Spatial proteomics resolves tumor and stromal proteomic microenvironment in TNBC. **A** Boxplots depicting the protein group and peptide identifications from tumor and stromal regions collected from FFPE TNBC tissue (*n* = 7, biological replicates). **B** Venn diagram showing protein group identifications overlap between cancer and stromal microdissected regions. **C** Principal Component Analysis (PCA) depicting differences between tumor and stromal samples based on spatial origin. **D** Heatmap displaying distinct clustering of tumor vs. stromal proteome signatures (red and blue, respectively). **E** Differential expression profiling of the Cancer and Stromal microdissected regions in a volcano plot. Significantly regulated proteins at 1% false discovery rate (FDR) are delimited by dashed lines, respectively (FDR controlled, two-sided *t* test, randomizations = 250, s0  =  0.1). All box plots include the median line; the box denotes the interquartile range (IQR), whiskers denote the rest of the data distribution and outliers are denoted by points greater than ±1.5 × IQR.

While bulk proteomics of cancer-invaded tissues can provide deep insights into the state of the disease, this method has the limitation of averaging the protein signals from all the different cells involved in the disease process^19^. In contrast, single-cell proteomics (SCP) based on single-cell suspensions can detect molecular signals from individual cells but loses spatial information^20,21^, which restricts the investigation of the complex interactions between malignant and non-malignant cells. Conversely, spatial proteomics allows for the capture of molecular signals at single-cell resolution while preserving spatial context^1,3^. To test this, we collected cancer and stroma cell regions of 20,000 µm^2^ from 10 µm-thick FFPE triple-negative breast cancer samples (Supplementary Fig. 6A). Reassuringly, our method enables us to identify approximately 4500 and 5500 protein groups, alongside around ~33,000 and ~50,000 modified sequences from stromal and cancerous regions, respectively, with a good overlap between the samples (Fig. 3A, B). Principal component analysis (PCA) revealed that tissue architecture of the sampled regions was the major source of variation in the samples (Fig. 3C). The heatmap analysis revealed distinct clustering patterns based on the sample regions and protein intensity (Fig. 3D). The differential protein abundance analysis between stromal and cancerous regions identified 398 protein groups upregulated in cancerous regions and 407 protein groups upregulated in stromal regions, respectively (Fig. 3E). Among the proteins upregulated in the tumor regions, we identified key cancer-related proteins such as KRT8, FTH1, TOP2A, CDKN2A, which are recognized hallmarks of triple-negative breast cancer (TNBC)^22–25^. In fact, it has been reported that KRT8 plays a role as a luminal marker overexpressed in invasive epithelia^26^, whereas TOP2A, an enzyme essential for DNA replication and cancer cell proliferation, serves as a direct or indirect target of several cytotoxic anti-cancer agents^22^. Moreover, FTH1 has been identified as a prognostic marker for triple-negative breast cancer^24^. In contrast, the stromal regions showed a distinct molecular signature, with proteins such as COL1A1, COLA2, DCN, and LUM upregulated in the stroma. In the stromal microenvironment, fibrillar collagens are known to accumulate in breast cancer and often appear as poorly defined spiculated masses in mammographic imaging^27^. Their abundance has been associated with multiple clinicopathological features, including tumor progression and patient outcome^28^. Additionally, the proteoglycans DCN and POSTN exhibit context-dependent roles, frequently functioning as endogenous regulators that modulate growth factor signaling and collagen organization, thereby helping to restrain tumor progression^29,30^. Consistent with these observations, gene ontology (GO) and KEGG pathway analyses revealed that stromal regions were enriched in biological processes such as extracellular matrix organization, immune response, B cell-mediated immunity, and wound healing (Supplementary Fig. 6B), indicating active fibrotic and immune processes. In contrast, tumor regions were enriched for processes including RNA splicing, ribonucleoprotein complex biogenesis, and mRNA metabolic pathways (Supplementary Fig. 6C), supporting enhanced cellular proliferation and invasive potential. The compartment-specific protein and pathway signatures identified here are in strong agreement with known biological features of tumor and stromal microenvironments, demonstrating the robustness of our workflow in capturing molecular characteristics of the tumor microenvironment.

### Spatial characterization of the tumor microenvironment in Luminal B breast cancer at subcellular resolution

**Fig. 4:**
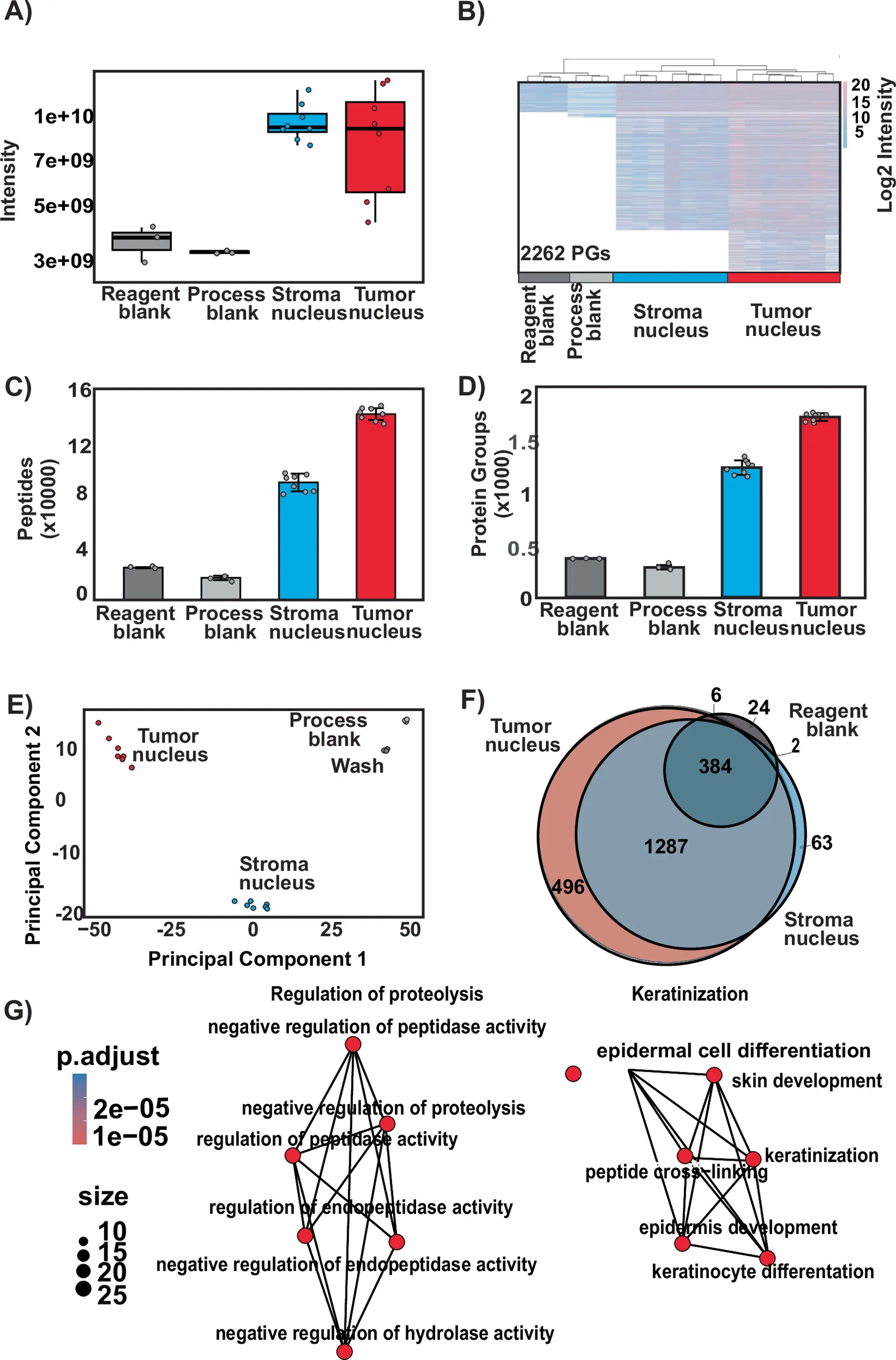
Benchmark of subcellular-resolution spatial proteomics in nuclear-enriched microregions. **A** Boxplot depicting the difference between Reagent blank (*n* = 3 technical replicates), Process blank (*n* = 3 technical replicates), Stroma and Nucleus (single nuclei from stroma, *n* = 8 biological replicates), and Tumor_nucleus (single nuclei from tumor, *n* = 8 biological replicates). **B** Heatmap showing protein expression intensity across different samples. **C** Bar plot showing peptide identification counts from nuclear-enriched stromal and tumor regions (~60 µm²), as well as reagent and process blanks; Reagent blank (*n* = 3 technical replicates), Process blank (*n* = 3 technical replicates), Stroma and Nucleus (single nuclei from stroma, *n* = 8 biological replicates), and Tumor_nucleus (single nuclei from tumor, *n* = 8 biological replicates). **D** Same as (**C**) but showing the protein group counts. **E** Principal component analysis illustrating differences between samples in score space. **F** Venn diagram showing the overlap of protein group identifications between blank samples and nuclear proteomes derived from distinct regions (stroma and tumor). **G** Gene Ontology (GO) enrichment analysis of biological process (BP) terms for proteins identified in blank samples. Statistical significance was determined using a one-sided hypergeometric test with Benjamini-Hochberg correction (adjusted *p*-value < 0.05). All box plots include the median line, the box denotes the interquartile range (IQR), whiskers denote the rest of the data distribution and outliers are denoted by points greater than ±1.5 × IQR. Data are presented as mean ± SD.

Spatial proteomics has primarily focused on tissue-level protein mapping; an emerging frontier is subcellular proteomics, which aims to resolve protein distribution at organelle-scale resolution^31^. This approach is essential for understanding molecular function in situ. Yet conventional approaches rely on organelle enrichment from bulk tissue, obscuring cellular heterogeneity. Therefore, we tested our optimized micro-FFPE workflow for high-sensitivity subcellular spatial proteomics profiling by collecting microdissected ~60 µm² regions from 5 µm-thick HER(–) ER(+) PR(+), P53(+) sections (Table 2.), targeting distinct tumor and stromal areas. Although the resolution of laser-capture microdissection (LMD) does not allow isolation of single, pure nuclei, the sampled regions were enriched for single nuclei, enabling robust proteomic profiling across tissue compartments (Supplementary Fig. 7A). To ensure that the detected signal originated from the collected material rather than from carry-over or background contamination introduced during collection or processing, we included two blank controls: a process blank (mimicking the whole workflow, including sampling and collection with an empty LMD membrane processed through all steps) and a reagent blank (with no sampling steps). Encouragingly, the signal intensity of the samples was more than three-fold higher than that of the blanks (Fig. 4A, B). These controls were processed in the same batch as single-nuclei samples derived from tumor (Sn-T) and stroma (Sn-S) regions. Consistent with this, we observed a marked increase in identification rates of protein groups (PGs) and peptides relative to blanks, with Sn-S yielding ~3.3-fold more PGs (~1300) and ~20-fold more peptides (~8000), and Sn-T yielding ~4.5-fold more PGs (~1800) and ~35-fold more peptides (~14,000). (Fig. 4C, D). Moreover, PCA revealed clear separation between blanks and single-nuclei samples, with tumor and stroma nuclei also segregating according to their spatial origin in score space, despite sharing ~20% of identified PGs (Fig. 4E, F). To further investigate the co-identified protein groups (PGs) in blanks and samples, we performed Gene Ontology (GO) enrichment analysis, which revealed two main molecular signatures: regulation of proteolysis and peptidase activity, and keratinization (Fig. 4G, E). These terms are consistent with trypsin-related proteases and skin-derived keratins (epithelial debris), which are common contaminants in proteomics workflows^32^.

**Fig. 5:**
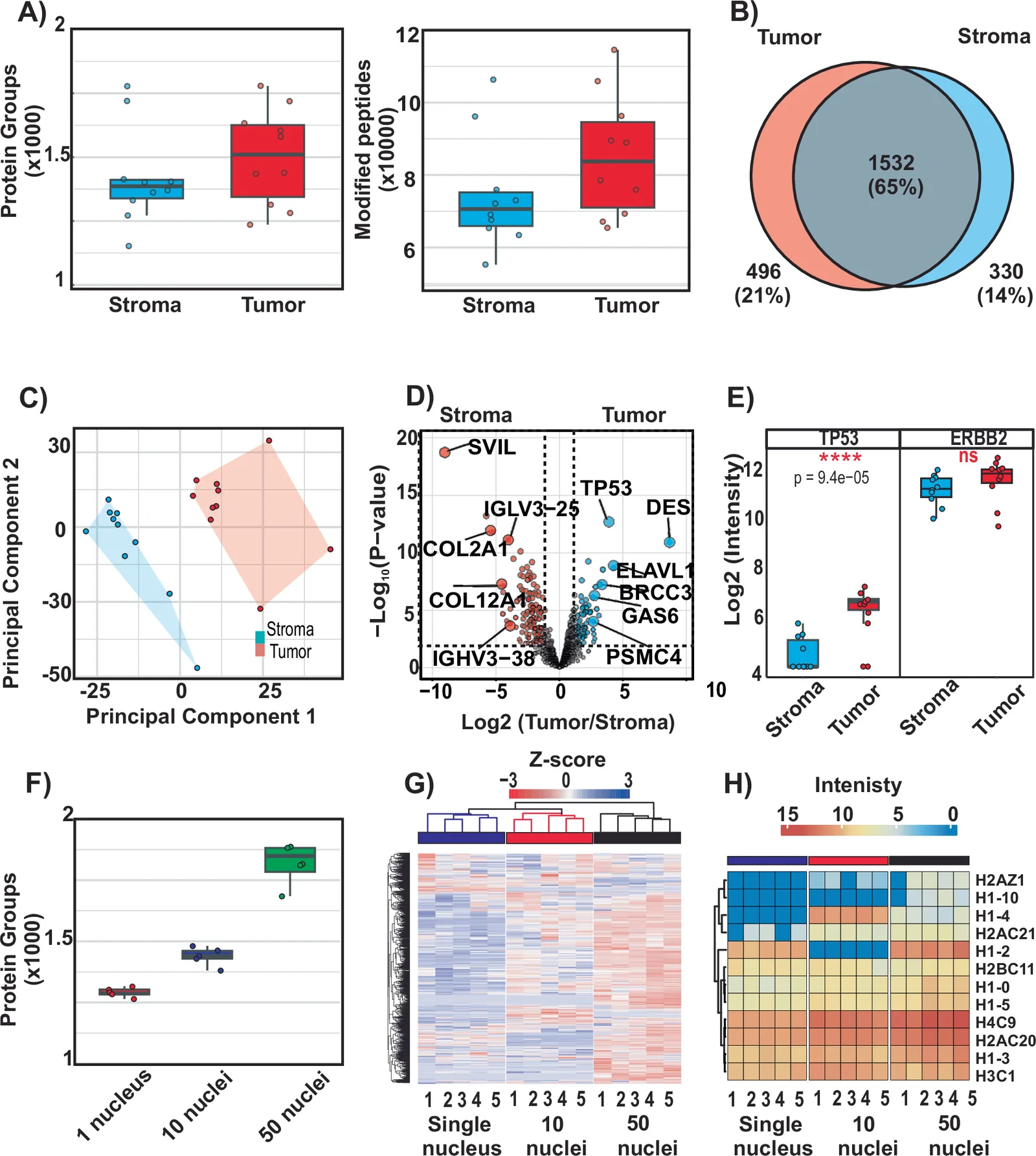
Subcellular-resolution spatial proteomics of nuclear-enriched micro-region. **A** Box plots depicting the proteome depth (Protein Groups and Modified sequences) from nuclear-enriched regions (~60 μm², *n* = 10, biological replicates). **B** Venn diagram showing protein group identifications overlap between nuclear proteomes derived from distinct regions (Stroma and Cancer). **C** Principal Component Analysis (PCA) depicting differences between tumor vs. stromal nuclear states. **D** Differential protein expression profiling enriches tumor and cancer nuclei in a volcano plot. Significant regulated proteins at 1% false discovery rate (FDR) are delimited by dashed lines, respectively (FDR controlled, two-sided *t* test, randomizations = 250, s0  =  0.1). **E** Boxplots depicting clinically relevant cancer markers (ERBB2 and TP53) at subcellular scale (*n* = 10, biological replicates). Statistical significance was assessed using a two-sided *t*test (*P* < 0.05). **F** Boxplot displaying the protein group identifications from 1 to 50 nuclei inputs (*n* = 5, biological replicates). **G** Heatmap showing clustering groups samples by input level. **H** Heatmap displaying the nucleosome coverage across distinct input amounts (*n* = 5). All box plots include the median line, the box denotes the interquartile range (IQR), whiskers denote the rest of the data distribution, and outliers are denoted by points greater than ±1.5 × IQR.

The proteome profiling of ten individual nuclear-enriched regions yielded slightly higher identifications on tumor than stroma (~1500 vs ~1,400 PGs; ~8500 vs 7500 modified peptides; Fig. 5A), although the two proteomes were largely overlapping (Fig. 5B). PCA further resolved the samples into distinct tumor and stromal nuclear states (Fig. 5C). The differential expression analysis of the nuclear-enriched regions identified 115 proteins upregulated in tumor and 90 PGs in stroma. Tumor nuclei were enriched for cancer- and nucleus-associated proteins, including TP53, BRCC3, ELAVL1, and PSMC4, whereas stromal nuclei showed elevated levels of proteins linked to extracellular matrix organization, fibrosis, and immune regulation, such as COL2A1, COL12A1, IGLC3, and IGHV3 (Fig. 5D). These experiments enabled observation of patterns consistent with established biological differences between tumor and stroma and with trends reported from larger-scale sampling of similar regions (Supplementary Fig. 7A). Although estrogen receptor (ER) and progesterone receptor (PR) were not detected, we identified and quantified clinically relevant cancer markers, including ERBB2 (Receptor tyrosine-protein kinase erbB-2) also known as HER2, and TP53, with the latter showing a significant difference between tumor and stromal nuclei (Fig. 5E). Overall, these findings demonstrate the applicability of our method for recovering biologically meaningful compartment-specific signals in clinical FFPE tissue, while being derived from a single-patient dataset. To further assess the sensitivity of our workflow at the subcellular level, we collected 1, 10, and 50 subcellularly enriched nuclei samples (*n* = 5). As expected, base peak intensity increased proportionally with the sample input (Supplementary Fig. 7B), and the number of identified protein groups ranged from ~1200 to ~1800, with ~7000 to ~ 13,000 unique peptides detected (Fig. 5F). Unsupervised hierarchical clustering revealed sample grouping based on input material (Fig. 5G), and most nucleosome subunit proteins were detected even at the single-enriched nuclei level (Fig. 5H). Similarly, the Pearson correlation analysis between samples showed the same trend (Supplementary Fig. 7C). GO analysis of the top 20 PGs detected showed strong enrichment for nuclear-related cellular components (Supplementary Fig 7D). Altogether, these results demonstrate that our optimized workflow can detect quantitative differences between subcellular structures from distinct histological regions while preserving biologically relevant information.

### Spatial profiling of the tumor microenvironment in breast cancer subtypes

**Fig. 6:**
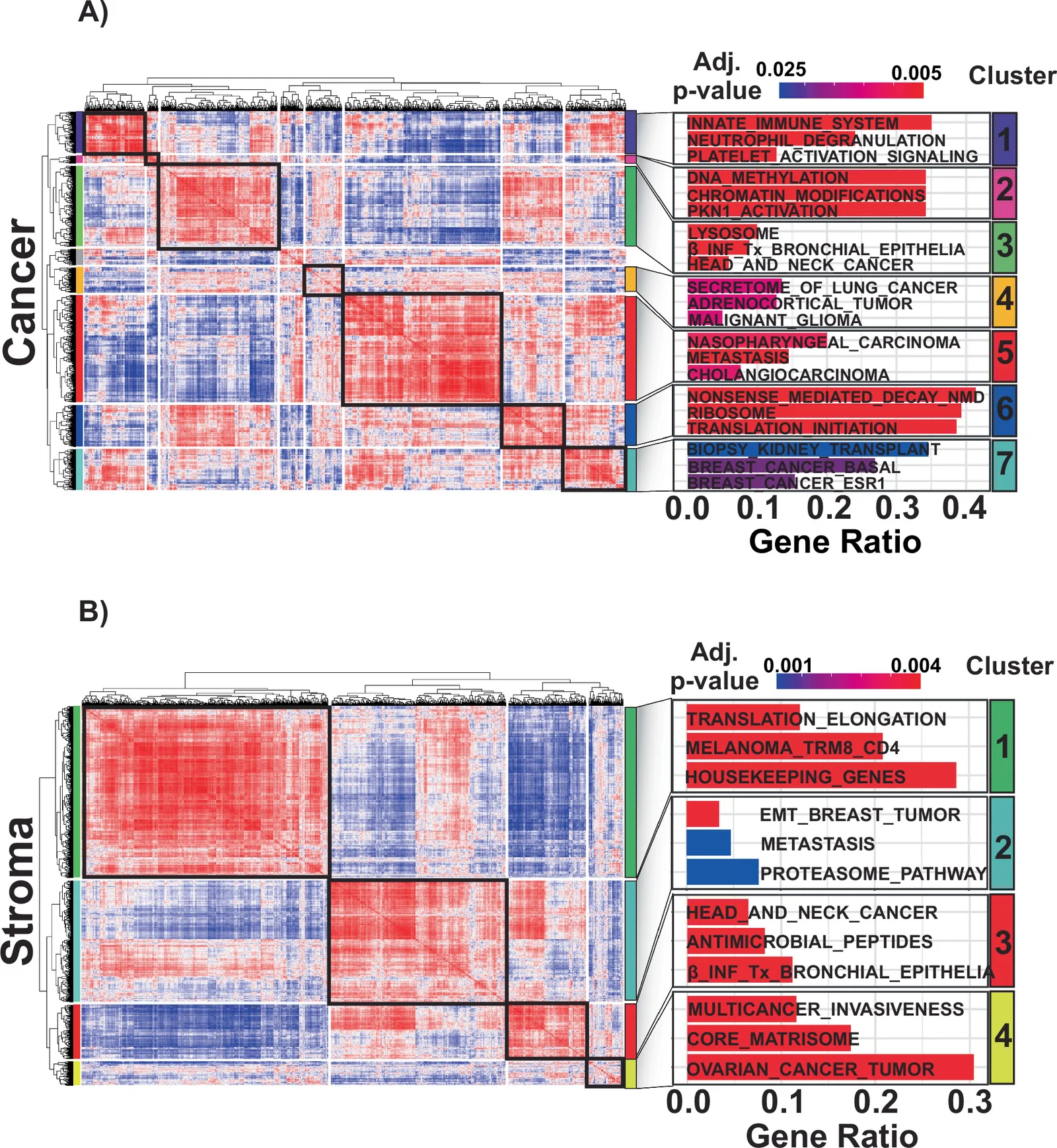
Spatial proteomic mapping of tumor and stromal compartments across breast cancer subtypes. **A** Pearson correlation analysis across all quantified protein groups identifies distinct co-regulated molecular modules within the tumor (upper panel) and **B** stromal (lower panel) compartments among ER⁻/PR⁻/HER2⁺, ER⁺/PR⁺/HER2⁺, ER⁺/PR⁺/HER2⁻, and triple-negative (TNBC) subtypes. Functional enrichment by module and microdissected region is presented in the left panel. Terms are ranked by GeneRatio. Statistical significance was assessed using a one-sided hypergeometric test with Benjamini–Hochberg correction (adjusted *P* < 0.05).

**Fig. 7:**
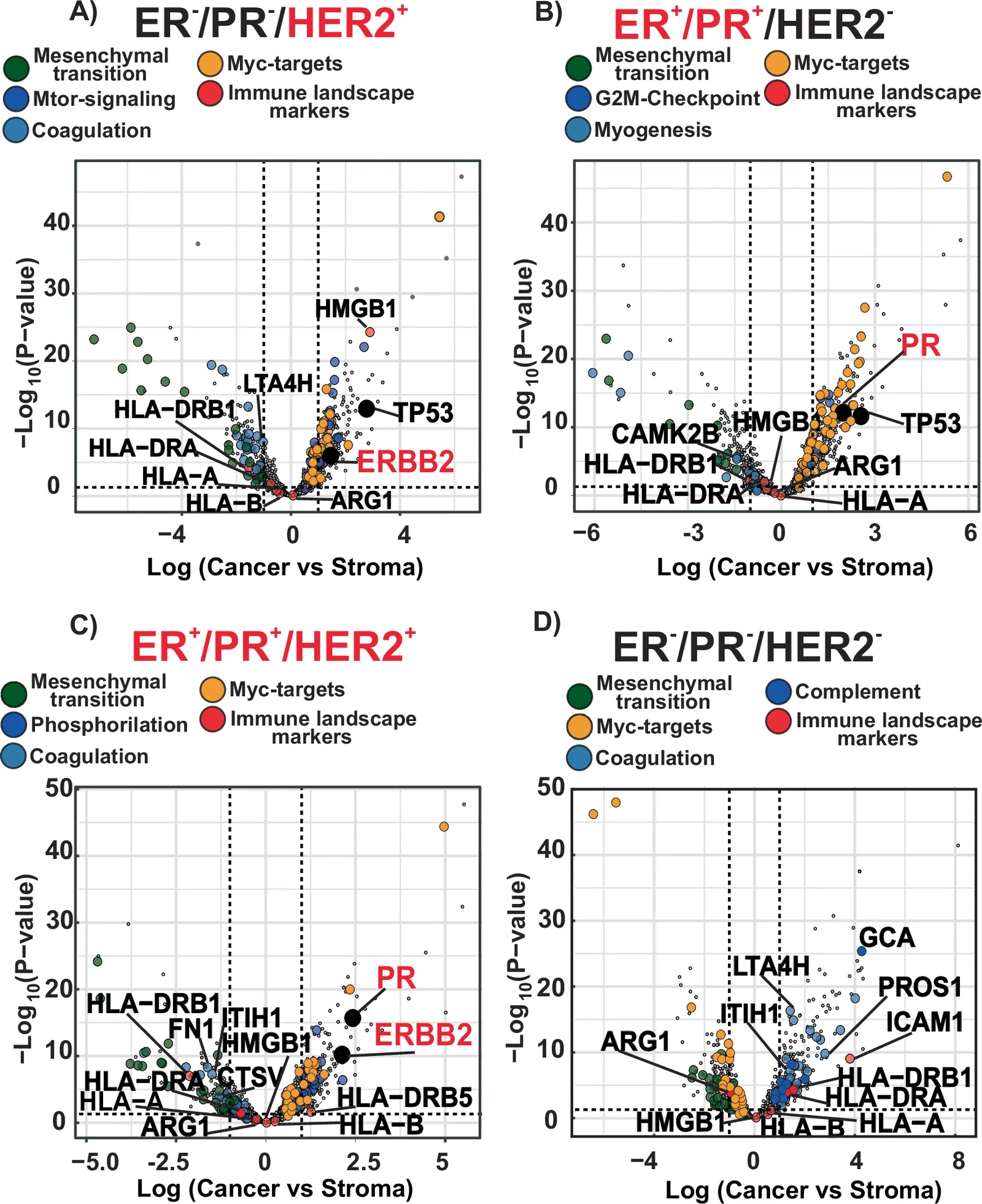
Spatial proteomic analysis of protein abundance changes in tumor and stromal regions across breast cancer subtypes. **A** Differential protein expression profiling of tumor regions compared to their corresponding stromal regions by subtype in a volcano plot. **A** ER⁻/PR⁻/HER2⁺, **B** ER⁺/PR⁺/HER2⁺, **C** ER⁺/PR⁺/HER2⁻, and **D** triple-negative (TNBC). Significantly regulated proteins at 1% false discovery rate (FDR) are delimited by dashed lines, respectively (FDR controlled, two-sided *t* test, randomizations = 250, s0  =  0.1). Molecular signatures were determined by statistical significance assessed using a one-sided hypergeometric test with Benjamini–Hochberg correction (adjusted *P* < 0.05).

The four major breast cancer subtypes are Luminal-A, Luminal-B, HER2-positive, and triple-negative. These categories are determined by the tumor’s status regarding the hormone receptor sestrogen receptor (ER) and progesterone receptor (PR), as well as the human epidermal growth factor receptor 2 (HER2). Each subtype has different growth rates, prognoses, and responses to treatment^33^. To investigate subtype-specific features of the tumor microenvironment across the four major breast cancer subtypes: HER2-positive (ER⁻/PR⁻/HER2⁺), Luminal-B (ER⁺/PR⁺/HER2⁺), Luminal-A (ER⁺/PR⁺/HER2⁻), and triple-negative (ER⁻/PR⁻/HER2⁻), we microdissected ~4000 μm² and 5 μm thickness regions (~10 cells equivalent) from paired tumor and stromal compartments and analyzed them using our optimized spatial-proteomics workflow (Table 2). Hierarchical clustering based on proteins significantly differing across subtypes (two-way ANOVA; FDR 0.05) revealed pronounced molecular divergence between regions (Supplementary Fig. 8A, B). Within tumor regions, the ER⁻/PR⁻/HER2⁻ (triple-negative) subtype showed strong enrichment for immune-related proteins, consistent with previous reports of increased immune infiltration and antigen-presenting cell engagement in triple-negative breast cancer (TNBC)^34,35^. In contrast, the HER2 and/or hormone receptor positive subtypes showed enrichment for proteins associated with translational machinery, aligning with luminal and HER2-driven proliferative programs^36^. Conversely, stromal regions exhibited subtype-specific extracellular matrix (ECM) remodeling signatures: ER⁻/PR⁻/HER2⁺, ER⁺/PR⁺/HER2⁺, and ER⁺/PR⁺/HER2⁻ stroma were enriched for collagen and fibronectin-associated matrix organization, in agreement with known fibroblast-mediated ECM remodeling in hormone receptor–positive and HER2⁺ tumors^37,38^. In contrast, ER⁻/PR⁻/HER2⁻ stroma showed greater enrichment of translational and metabolic pathways, suggesting a distinct stromal activation state characteristic of TNBC-associated microenvironments^39^. To resolve molecular signatures across cancer subtypes and regions, we performed correlation analysis across all identified protein groups, irrespective of the cancer subtype, revealing co-regulated molecular modules. Seven distinct signatures were observed in tumor regions and four in stromal regions (Fig. 6A, B). Examination of tumor-associated modules highlighted subtype-specific differences; for example, the metastasis-associated cluster comprising proteins with significant subtype-dependent variation, showed upregulation of S100P and EVPL in the ER⁻/PR⁻/HER2⁻ (triple-negative) subtype, consistent with previous reports linking S100P to TNBC metastasis^40^(Supplementary Fig. 9A). Within the breast cancer-associated cluster, as expected, PR was detected in ER⁺/PR⁺/HER2⁺ and ER⁺/PR⁺/HER2⁻ subtypes^40^(Supplementary Fig. 9B). Notably, the immune system-associated module exhibited upregulation of proteins including SERPINA1, SERPINB1, and LRG1; the latter is a glycoprotein known to promote angiogenesis and tumor progression, and its elevated expression in breast cancer has been associated with lymphatic metastasis and poor patient survival^41^(Supplementary Fig. 9C). In contrast, the metastasis-associated module derived from the stromal compartment showed the upregulation of proteosome subunits, including PSMA1, PSMB1 and PSMB2 across the luminal B and triple-negative breast cancer subtypes analyzed. Notably, PSMA1 has been previously implicated in breast tumor progression, underscoring its potential role in supporting malignant growth^42^. More broadly, coordinated expression of proteasome gene signatures has been associated with high tumor grade and aggressive phenotypes in breast cancer, highlighting the contribution of proteasomal regulation to tumor malignancy^43^(Supplementary Fig. 10A). Within the matrisome-associated cluster, the Luminal A and HER2-positive subtypes showed increased abundance of collagen matrix proteins (Supplementary Fig. 10B), including COL1A1, COL1A2, and COL6A1, compared with luminal B and triple-negative breast cancer subtypes, suggesting differential collagen matrix deposition between cancer subtypes^44,45^. Moreover, the triple-negative subtype exhibited marked activation of Rho-GTPase signaling (Supplementary Fig. 10C), alongside elevated expression of S100A11 and S100A4, proteins associated with the promotion of breast cancer cell invasiveness^46–48^. Finally, to assess compartment-specific differences, we compared tumor and stromal regions within each subtype. This analysis reliably recovered the expected cell-type–specific markers, with ERBB2 more abundant in cancer cells than in stroma in HER2-positive and luminal-B samples (Fig. 7A, C), whereas PR was more abundant in cancer cells than in stroma in luminal-A and luminal-B samples, respectively (Fig. 7B, C). No differentially regulated markers were observed in the triple-negative breast cancer samples (Fig. 7D). Across most subtypes, we observed broad activation of the MYC pathway and epithelial-to-mesenchymal transition (EMT). In the triple-negative subtype, MYC pathway activation was detected predominantly in the stromal compartment, suggesting that stromal cells may contribute to the aggressive TNBC phenotype^49^. Alternatively, this signal could reflect cancer-cell infiltration into the stroma or paracrine induction of MYC signaling in stromal cells^50^. We also identified immune microenvironment–associated markers, including HMGB1 (Fig. 7A), which was upregulated in HER2-positive tumor regions and has been clinically explored as an indicator of tumor immunogenicity and potential response to chemotherapy in this breast cancer subtype^51,52^. Although these experiments were conducted using samples from a single patient per cancer subtype, they demonstrate the applicability of our method by capturing biologically meaningful differences both between tumor and stromal regions and across cancer subtypes. However, we acknowledge that these observations cannot yet be generalized and may in part reflect inter-patient variability rather than true subtype-specific differences.

## Discussion

Spatial proteomics aims to characterize protein expression within the structural context of tissues, enabling insights into cell–cell communication, microenvironmental influences, and functional specialization of cell states that are not captured by bulk or dissociated single-cell proteomics^53^. However, the widespread adoption of spatial proteomics has been constrained by practical challenges, particularly the efficient extraction and digestion of proteins from microscale tissue regions, as well as the use of specialized equipment or low-throughput methods to tackle these challenges. To address this, we introduce a scalable high-throughput workflow based on the hanging droplet concept that enables reproducible processing of up to 96 laser-microdissected samples in parallel, generating LC-MS–ready material in ~2 h. Our streamlined approach achieves robust proteome coverage from low-input samples, recovering ~5500 PGs from input equivalent to ~250 cells and ~2500 PGs from input equivalent to ~5 cells. Moreover, the workflow supports reproducible subcellular-scale profiling, recovering ~1800 protein groups from nuclear-enriched regions as small as ~60 µm², approaching near-organelle resolution while preserving cell-type–specific molecular signatures. The biological application of this workflow to triple-negative breast cancer (TNBC) tissues revealed robust compartment-specific proteomic programs distinguishing tumor from stromal regions. Tumor regions were enriched for RNA processing pathways, proliferative signaling networks, and canonical cancer-associated markers, whereas stromal regions exhibited signatures of extracellular matrix remodeling, immune activation, and fibrotic repair. Extending this analysis across multiple breast cancer subtypes uncovered distinct molecular programs within both tumor and stromal compartments, highlighting subtype-dependent differences in microenvironmental composition and signaling dynamics. Notably, our approach demonstrates the ability to detect biologically meaningful protein signatures from regions representing as small as ~60 μm², bridging the sensitivity gap between single-cell proteomics and tissue- or subcellular-level spatial resolution, without the need for highly specialized instrumentation. Altogether, our results support that our approach provides a scalable, reproducible, and ultrasensitive spatial proteomics workflow that unlocks micro-regional and subcellular proteomic profiling in FFPE tissues. By integrating established clinical tissue resources with efficient processing and high-sensitivity nDIA mass spectrometry, this method represents a critical step toward routine spatial proteomics in translational research and precision pathology.

## Methods

### Materials

Formalin-fixed paraffin-embedded (FFPE) human triple-negative breast cancer tissue slides, measuring 5 µm and 10 µm in thickness, embedded in a 2.0 um PEN membrane glass slides were obtained from VitroVivo Biotech. (VitroVivo Biotech LLC P.O. Box 83644 Gaithersburg, MD 20883) and processed as follows.

### H&E staining

Prior to analysis, formalin-fixed paraffin-embedded (FFPE) slides were dewaxed in xylene for 3 min, repeated twice. Following dewaxing, samples were subjected to a gradient hydration protocol, which included immersion in 100% ethanol for 1 min (twice), followed by 95% ethanol for 1 min, 70% ethanol for 1 min, and finally distilled water for 3 min. Subsequently, the slides were stained with hematoxylin for 1 min. Excess of hematoxylin was removed with distilled water before staining with Eosin for 10 s, after which any remaining stain was also washed off with distilled water. For directly capturing samples using the laser capture microdissection (LMD) system, the stained slides were air-dried and stored at −20 °C. For annotation and more accurate sample capture based on clear images, samples were sealed with an anti-fade fluorescence mounting medium and covered with a glass coverslip.

### Visualization and annotation

Images captured at 20X or 40X magnification using the Axioscan 7 microscope are imported into QuPath 0.5.1^54^, where regions of interest (ROIs) are marked and annotated before being exported as a GeoJSON file. The web application “Steamlist(https://qupath-to-lmd-mdcberlin.streamlit.app/)” then converts the GeoJSON file into an XML file, which contains the coordinate information necessary for the Laser Microdissection (LMD) microscope to identify the samples^55^. For sample collection, a proteoCHIP EVO 96 (cellenONE X1 Neo) is used as a holder, requiring an adapter to secure the EVO 96 chip within the LMD shelter. This adapter was 3D printed, with the original design from Makhmut et al., 2024^9^. Prior to laser capture, the coverslips and mounting medium should be removed by incubating the slides in 1x PBS at 37 °C for 30 min. Subsequently, the sections should be air-dried for 30 min at 37 °C.

### Laser microdissection (LMD) microscope settings

The Leica LMD 7000 microscope was used for laser microdissection and sample collection according to the parameters specified in the XML file. The configuration for the laser parameters is as follows:

For large-area sample capture (>1000 µm²), laser microdissection was performed using a 10× objective. Laser settings were as follows: power, 60; aperture, 10; cutting speed, 8; middle pulse count, 4; final pulse, 8; head count, 69%; and pulse frequency, 2200. For single-cell or subcellular sample capture (<1000 µm²), laser microdissection was performed using a 63× objective. Laser settings were as follows: power, 46; aperture, 1; cutting speed, 10; middle pulse count, 4; final pulse, 0; head count, 55%; and pulse frequency, 2600. These parameters were optimized to ensure precise dissection and efficient collection of individual cells or subcellular regions while minimizing collateral damage to surrounding tissue.

Following the laser capture and collection process, verification performed by microscope was conducted to determine whether the samples had been successfully deposited into the wells of the chip.

### Sample preparation by hanging on droplet

To initiate the *de*crosslinking process, 13 µL of 25 mM TEAB buffer is added to the wells of the proteoCHIP EVO 96 chip, which is positioned on a standard 96-well plate (Armadillo 96-well plate with pre-chilling at −20 °C) with the pillars facing inward. The assembly is then incubated in a heating block at 90 °C for 40 min, with the heating block lid removed (see Supplementary Note 1). After the heating period, the EVO 96 chips are carefully removed from the 96-well plate and allowed to cool to room temperature for 5 min. Subsequently, to initiate the digestion process 2.5 µL of a 10 ng/µL trypsin and Lys-C mix is added. The EVO 96 chips are then placed back on the 96-well plate and incubated at 37 °C for 1 h. After digestion, the reaction is quenched by adding 1 µL of 10% formic acid (FA) to each well. Subsequently, samples were transferred to EvoTips for downstream cleanup and storage prior to LC–MS/MS analysis. Briefly, the transfer was performed by aligning the EVO 96 pillars with the Evotips mounted in a rack and applying a brief centrifugation (60 s at 800 × g) to drive the material into already equilibrated tips. Evotip loading was carried out according to the standard Evosep sample loading protocol: tips were first rinsed with solvent B (ACN, 0.1 %FA) and centrifuged, soaked in 1-propanol until fully wetted, then equilibrated with solvent A (0.1% FA). Peptide samples were loaded onto wetted Evotips and centrifuged at 800 × g to bind peptides to the C18 resin. After loading, tips were washed with solvent A and centrifuged again, followed by addition of solvent A and a short spin to keep the resin hydrated. Prepared Evotips were stored at 4 °C until LC–MS/MS analysis. Alternatively, the EVO 96 chips were suspended above a 96-well plate and stored at −20 °C until further processing. Two experimental scales were defined: “single-cell level” (≤5 cells; ~2000 µm², 5 µm-thick BC samples) and “micro-scale” (>5 cells). For single-cell level experiments, 2.5 µL of a 5 ng/µL trypsin/Lys-C mix was applied, corresponding to a total enzyme input of 12.5 ng. For micro-scale experiments, 2.5 µL of a 10 ng/µL enzyme solution was used. To ensure experimental consistency while minimizing batch effects, all optimization experiments were processed on Evo-96 plates in a single batch, enabling the successful processing and analysis of 192 samples in one workflow. Further detailed protocols, including additional operational details and troubleshooting guidance, are provided in Supplementary Note 1.

### Sample preparation by on-tip digestion

To initiate the *de*crosslinking process, 13 µL of 25 mM TEAB buffer was added to the wells of the EVO 96 chip, which is positioned on a standard 96-well plate with the pillars facing inward. The assembly is then heated in a heating block at 95 °C for 40 min. After the *de*crosslinking step, the EVO 96 chips were carefully removed from the 96-well plate and allowed to cool to room temperature for 5 min. Subsequently, 2.5 µL of a trypsin/Lys-C mixture (10 ng/µL) were added to each sample. Droplets were then transferred to Evotips by aligning the EVO 96 pillars with the tips mounted in a rack and applying a brief centrifugation pulse (20 s at 100 × g) to drive the material into the tips. Proteolytic digestion was carried out directly on already conditioned EvoTips by incubating at 37 °C for 1 h. The resulting peptide mixtures were desalted and concentrated on C18-reversed-phase material in Evotips following the vendor’s instructions. Briefly, to condition the Evotips, they were washed with 20 μl of Solvent B and centrifuged at 800 g for 60 s. To condition the Evotips, they were soaked in 100 μl of 1-propanol until they turned pale white. After this, they were equilibrated with 20 μl of Solvent A and centrifuged at 800 g for 60 s. After droplet transfer and on-tip digestion, samples were further loaded on Evotips by centrifugation at 800 for 60 s. The Evotips were then washed with 20 µL of solvent A and centrifuged at 800 × g for 60 s. Subsequently, 100 µL of solvent A was added to each tip and centrifuged at 800 × g for 10 s. Water was added to the Evotip box to the level of the C18 resin to prevent drying, and the tips were stored at 4 °C until LC–MS/MS analysis.

### LC separation and mass spectrometry analysis

All LC-MS/MS analysis was performed on an Orbitrap Astral MS using Thermo Tune software (version 1.0.100.40), coupled with an Evosep One system (EvoSep Biosystems) operated via Chronos software (Chronos 4.10.0.0, Chronos plugin 1.0.25.4). FFPE-derived peptide mixtures were analyzed using the predefined 80SPD whisper zoom method (5.6-minute gradient), using a 5-cm commercial analytical column (Aurora Rapid, IonOpticks), interfaced online with an EASY-Spray™ source. The Orbitrap Astral MS was operated in narrow-window data-independent acquisition (nDIA) mode^17^ with a full MS resolution setting of 240,000 and MS1 maximum injection time limited to 3 ms., covering a full scan range of 380–980 m/z. The full MS AGC was set to 500%. The nDIA isolation windows were set to 4 Th with a maximum ion injection time (IT) restricted to 6 ms. The MS/MS scanning range was set to 380–980 m/z, with a normalized collision energy (NCE) of 27%.

### Proteomics raw data analysis

Raw LC-MS files were analyzed in Spectronaut v19 and v20 (Biognosys) with a spectral library-free approach (directDIA  + ) using the human protein reference database (Uniprot 2024 release, 20,588 sequences) for all samples complemented with common contaminants (246 sequences). Note that, as the protocol does not involve reduction and alkylation, database searches were performed with free cysteine sulfhydryls; hence, cysteine carbamylation was not set as a fixed modification, whereas methionine oxidation and protein N-termini acetylation were set as variable modifications. Precursor filtering was set to perform based on Q-values, and cross-run normalization was checked. Method evaluation mode was enabled when method optimization experiments were conducted. Data analysis was performed in RStudio (v2026.01.1) using R (v4.4.2) and standard libraries, including ggplot2 (v3.5.1) and tidyverse (v2.0.0). For tumor microenvironment and subtype profiling at subcellular resolution, data were first log2-transformed and filtered for missing values, retaining proteins with up to 70% valid observations prior to downstream processing. Afterwards, imputation was performed using the Structured Least Squares Algorithm (SLSA) implemented in the DAPAR package with default parameters. Data were then normalized using the limma package (v3.60.0) via normalizeBetweenArrays with the cyclic loess (cyclicloess) method. Statistical analyses were performed using the limma package (v3.60.0), including moderated *t*tests and analysis of variance (ANOVA). GO term enrichment analysis was performed using the clusterProfiler package (v4.12.0) in R (v4.4.2).

### Inclusion and ethics statement

We are committed to promoting diversity and inclusion in science and ensuring that our research is conducted ethically and responsibly. Our study was designed and conducted in accordance with ethical principles and guidelines, including obtaining informed consent from all participants and complying with relevant regulations and laws.

### Reporting summary

Further information on research design is available in the Nature Portfolio Reporting Summary linked to this article.

## Supplementary information

Supplementary Information

Supplementary Movie 1

Reporting Summary

Transparent Peer Review file

## Source data

Source data

## Data Availability

Data is available via the ProteomeXchange with the identifier PXD078037. Source data are provided with this paper.
