# Supplementary material for: High-throughput spatial proteomics from cellular to subcellular scales via a hanging droplet workflow
Source: Nat Commun. 2026 Oct 9;17:11488. doi: 10.1038/s41467-026-78415-x (PMC13740325; doi:10.1038/s41467-026-78415-x)
Supplement: Supplementary file 1 [file 41467_2026_78415_MOESM1_ESM.pdf]

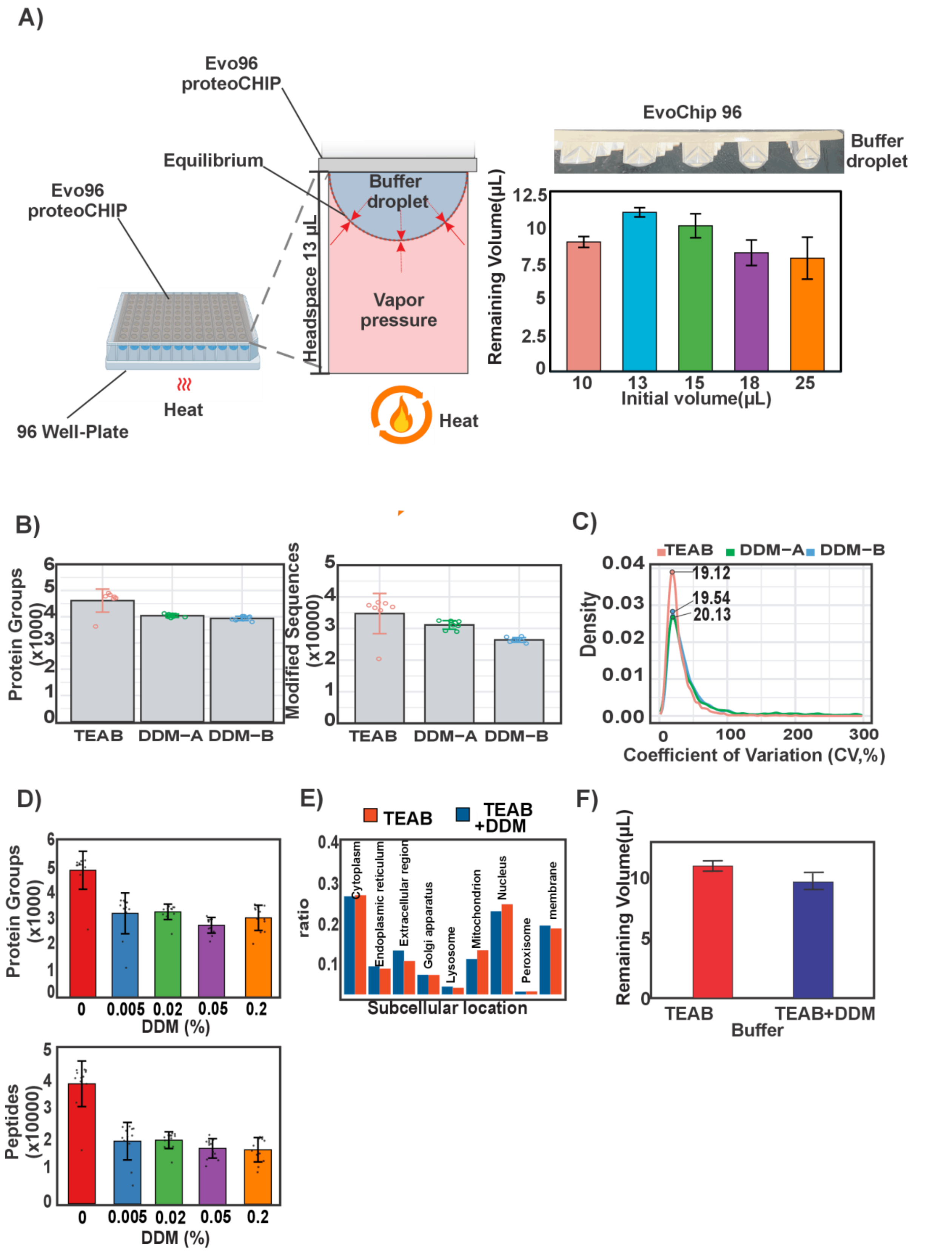
A)
Evo96
proteoCHIP
Equilibrium
Evo96
proteoCHIP
Buffer
droplet
Headspace 13 µL
Vapor
pressure
Heat
96 Well-Plate
Heat
EvoChip 96
Buffer
droplet
Remaining Volume(µL)
12.5
10
7.5
5
2.5
0
10
13
15
18
25
Initial volume(µL)
B)
Protein Groups
(x1000)
TEAB
DDM−A
DDM−B
Modified Sequences
(x10000)
TEAB
DDM−A
DDM−B
C)
TEAB
DDM−A
DDM−B
19.12
19.54
20.13
Density
Coefficient of Variation (CV,%)
D)
Protein Groups
(x1000)
0
0.005
0.02
0.05
0.2
DDM (%)
Peptides
(x10000)
0
0.005
0.02
0.05
0.2
DDM (%)
E)
TEAB
TEAB
+DDM
ratio
Cytoplasm
Endoplasmic reticulum
Extracellular region
Golgi apparatus
Lysosome
Mitochondrion
Nucleus
Peroxisome
membrane
Subcellular location
F)
Remaining Volume(µL)
TEAB
TEAB+DDM
Buffer

Supplementary Fig. 1. Conceptualization and optimization of the hanging droplet FFPE workflow. (A) Schematic illustration of the suspended droplet on the hanging droplet configuration on EVO96 chip pillars during decrosslinking (left panel). Reaction volume optimization during crosslinking conditions (right panel). **(B)** Comparison of Ammonium Bicarbonate (ABC) and Triethylammonium Bicarbonate (TEAB) buffer systems adding n-Dodecyl-β-D-maltoside (DDM) detergent before and after crosslinking process. **(C)** Distribution of Coefficients of Variation (CV) across different buffer systems adding DDM detergent before and after *de*crosslinking process. **(D)** Bar plots showing protein group (Top panel) and peptide identifications across varying DDM concentrations (%) in 25 mM TEAB buffer during digestion (Bottom panel). **(E)** Proportion of subcellular localization categories among proteins identified under optimal ABC and TEAB conditions (25 mM). **(F)** Remaining reaction volume after *de*crosslinking (90 °C, 30 min) in 25 mM TEAB buffer with and without DDM (0.005%). Data are presented as mean ± SD. Supplementary Fig. 1A was partially created in Biorender.com. Olsen, J. (2026) https://BioRender.com/g8xhcyq

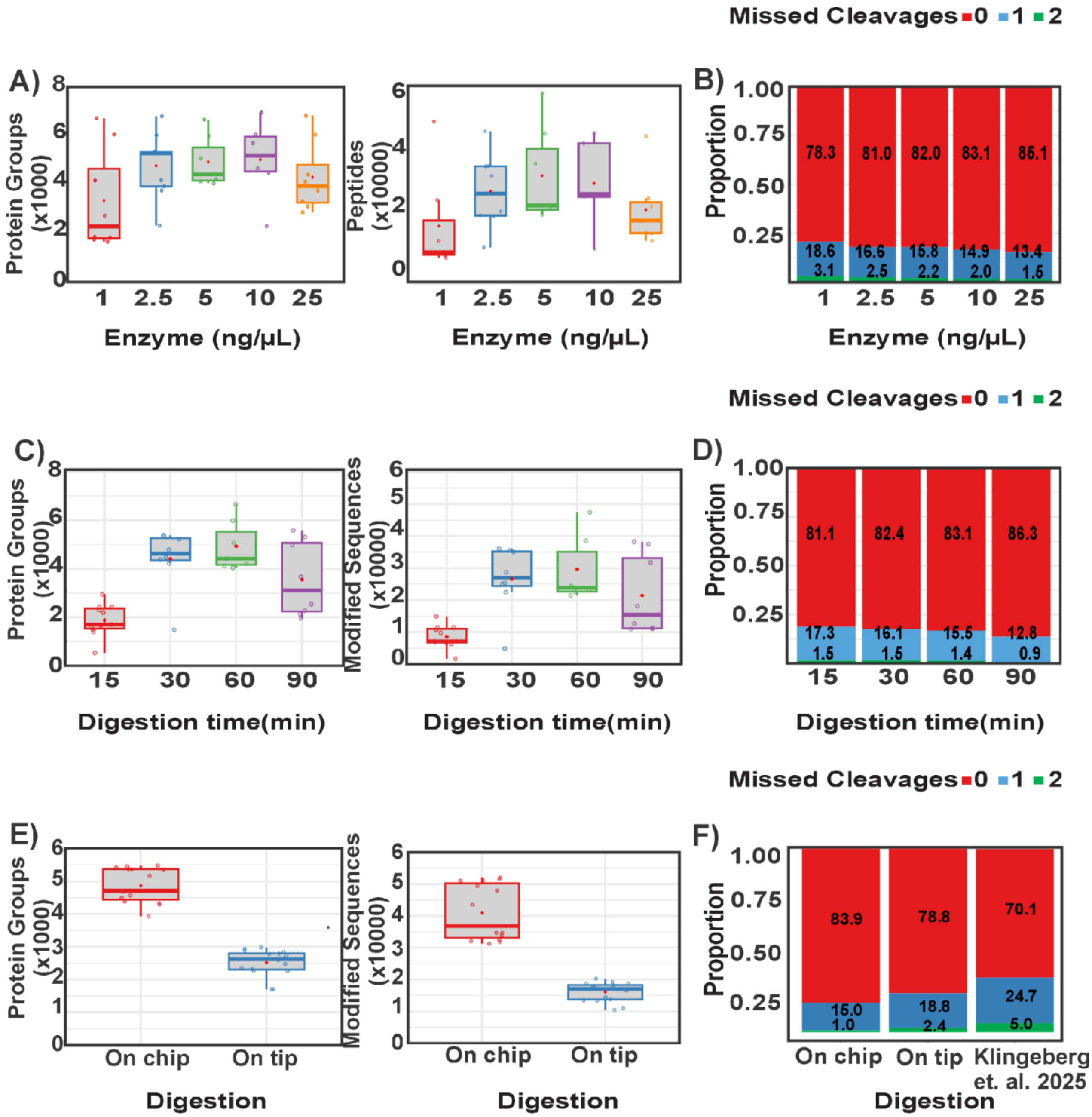

Supplementary Fig. 2. Optimization of hanging droplet–based rapid proteolytic digestion. (A) Box plots showing protein group and peptide identifications across different trypsin/Lys-C mixture concentrations. **(B)** Missed tryptic cleavage rates as a function of trypsin/Lys-C mixture concentrations. **(C)** Boxplots illustrating protein group and modified peptide identifications across digestion time conditions. **(D)** Percentage of peptides containing up to two missed cleavage sites across distinct digestion time conditions. **(E)** Benchmark comparison between On-Chip (Red) vs On-tip (Blue) digestion. **(F)** Percentage of peptides containing up to two missed cleavage sites in on-chip versus on-tip digestion, compared with the digestion performance reported by Klingeberg et al. (2025).

All box plots include the median line, the box denotes the interquartile range (IQR), whiskers denote the rest of the data distribution and outliers are denoted by points greater than ±1.5 × IQR. Red dots in the box plots indicate the mean.

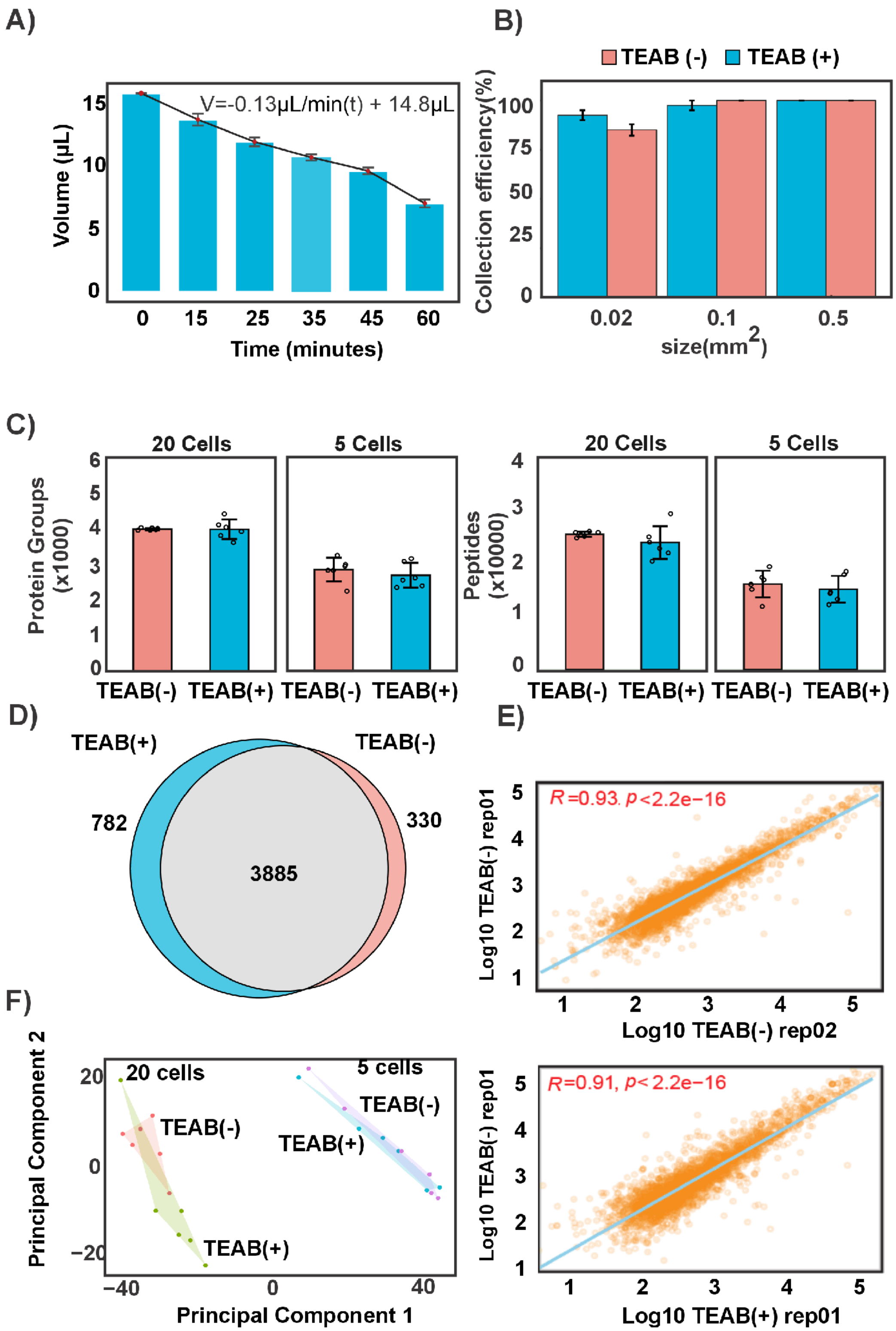

Supplementary Fig. 3. Recovery efficiency assessment in laser microdissection workflows. (A) Bar plot showing volume loss during sample collection under the laser capture microdissection microscope. The initial volume was 15 µL (n = 12, biological replicates), and the volume loss rate is indicated above the bars. **(B)** Collection efficiency across different sample sizes between the Triethylammonium bicarbonate TEAB buffer

added prior (TEAB (+)) and after (TEAB (-)) collection (n = 4, biological replicates). **(C)** Bar plots showing peptide (left panel) and protein group (right panel) identifications for 5- and 20-cell equivalents using TEAB (+) and TEAB (−) collection methods (n=6, biological replicates). **(D)** Venn diagram showing the overlap of protein groups identified using TEAB (+) and TEAB (-) collection methods. **(E)** Scatterplots depicting the Pearson correlation within replicates and different collection methods. **(F)** Principal component analysis (PCA) illustrating differences between TEAB (-) and TEAB (+) collection methods and input amounts. Data are presented as mean ± SD.

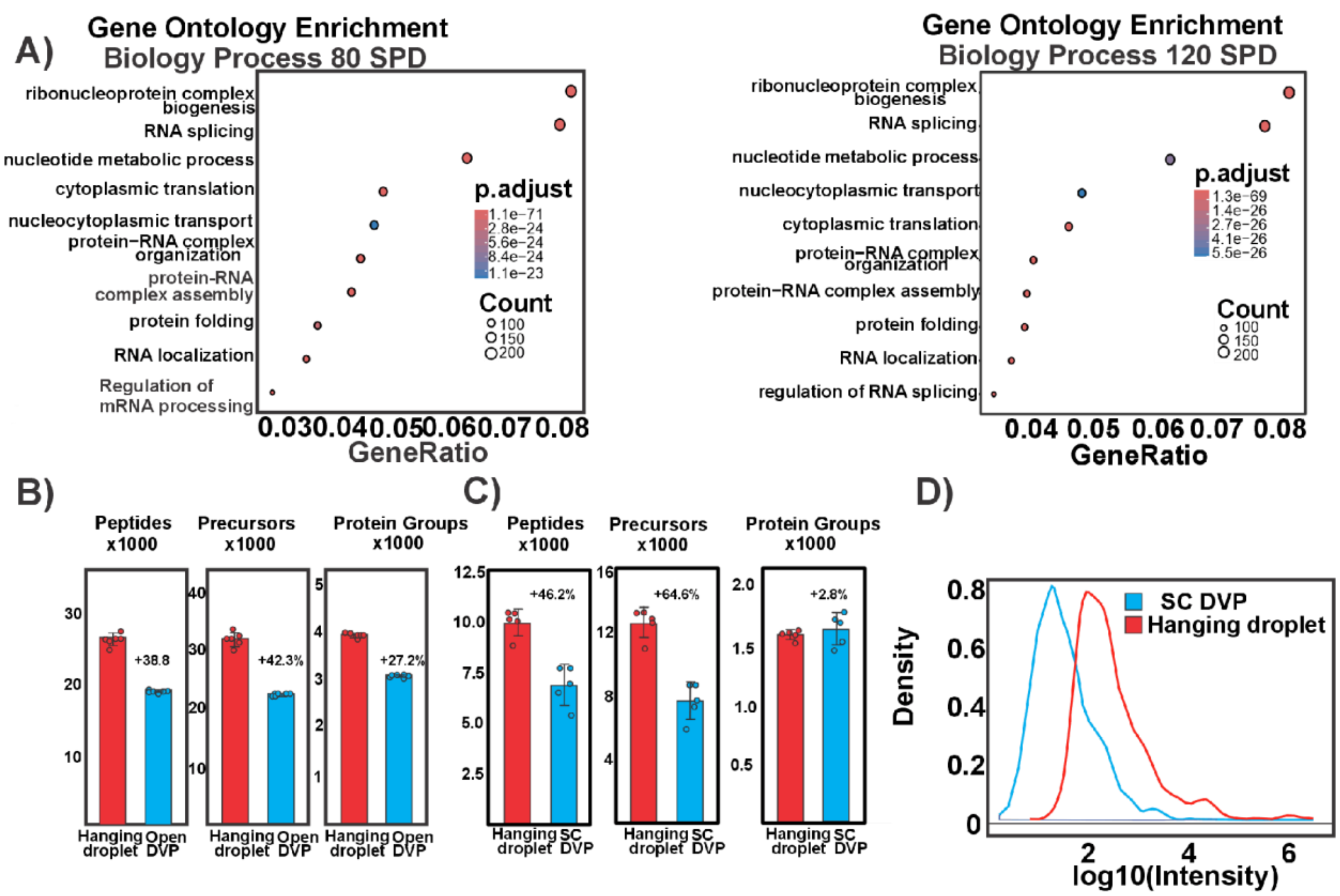

Supplementary Fig. 4. Benchmark of the hanging droplet method against existing spatial proteomics workflows. (A) Top ten Gene Ontology (GO) biological process (BP) terms enriched among proteins identified using the 80 samples-per-day (SPD) (right panel) and 120 SPD (left panel) methods. Terms are ranked by GeneRatio. Statistical significance was assessed using a hypergeometric test with Benjamini–Hochberg correction (adjusted P < 0.05). **(B)** Peptide, precursor, and protein group identifications from 8,000 µm² triple-positive breast cancer sections (5 µm thickness) analyzed by narrow-window DIA (nDIA) using the hanging-droplet method (80 SPD, Evosep one; Orbitrap Astral MS), compared to Open DVP DIA-PASEF (Tonsil regions, 8,000 µm², 5 µm thickness, Evosep, 80 SPD; timsTOF Ultra AIP), as reported by Klingeberg et al. (2026). **(C)** As in **(B),** but for single-cell–equivalent inputs, comparing the hanging-droplet method (80 SPD, Evosep one; Orbitrap Astral MS) to the scDVP workflow reported by Rosenberger et al. (2025) (40 SPD; FAIMS CV-40, Orbitrap Astral MS). **(D)** Log10-transformed intensity distributions of six single-cell–equivalent samples processed using the hanging-droplet method (triple-positive breast cancer regions, 5 µm), compared to single-cell DVP data (hepatocyte-like single shape, 10 µm thickness) reported by Rosenberger et al. (2025). Data are presented as mean ± SD.

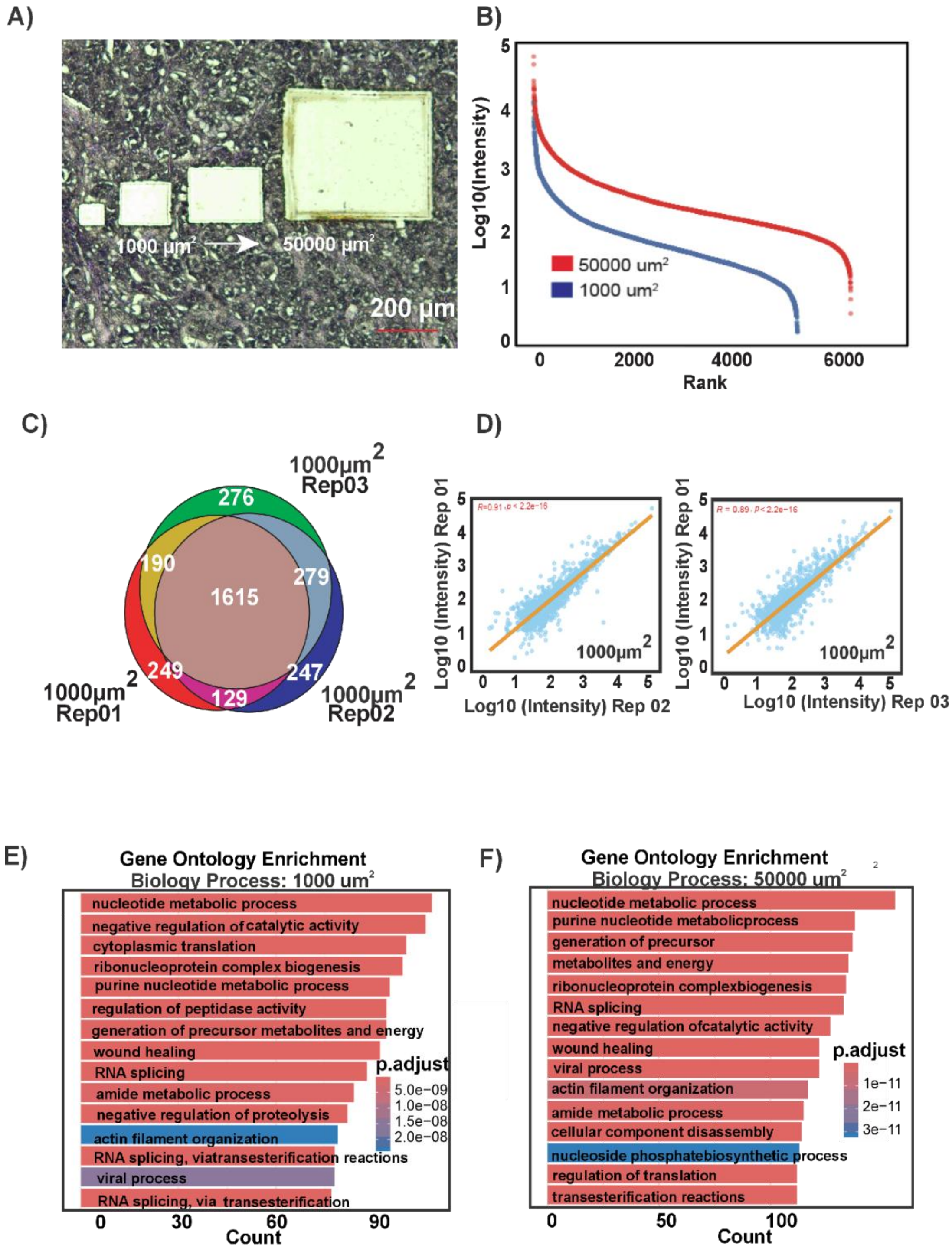

Supplementary Fig. 5. Proteome coverage and reproducibility across FFPE sample input amounts. (A) Microdissected region size representation ranging from ~1000 µm² to ~50,000 µm² (~5–250 cells). **(B)** Rank intensity plots showing the dynamic range across loading levels. **(C)** Venn diagram showing protein group identifications overlap between biological replicates. (n =3, 1000 µm² input)**. (D)** Scatter plot showing the Pearson correlation across biological replicates. **(E)** Top 15 Biological process GO-terms enriched

by Gene set enrichment analysis (GSEA) for samples with 1,000 µm² input area. **(F)** Same as **(E)**, but for samples with a 50,000 µm² input area.

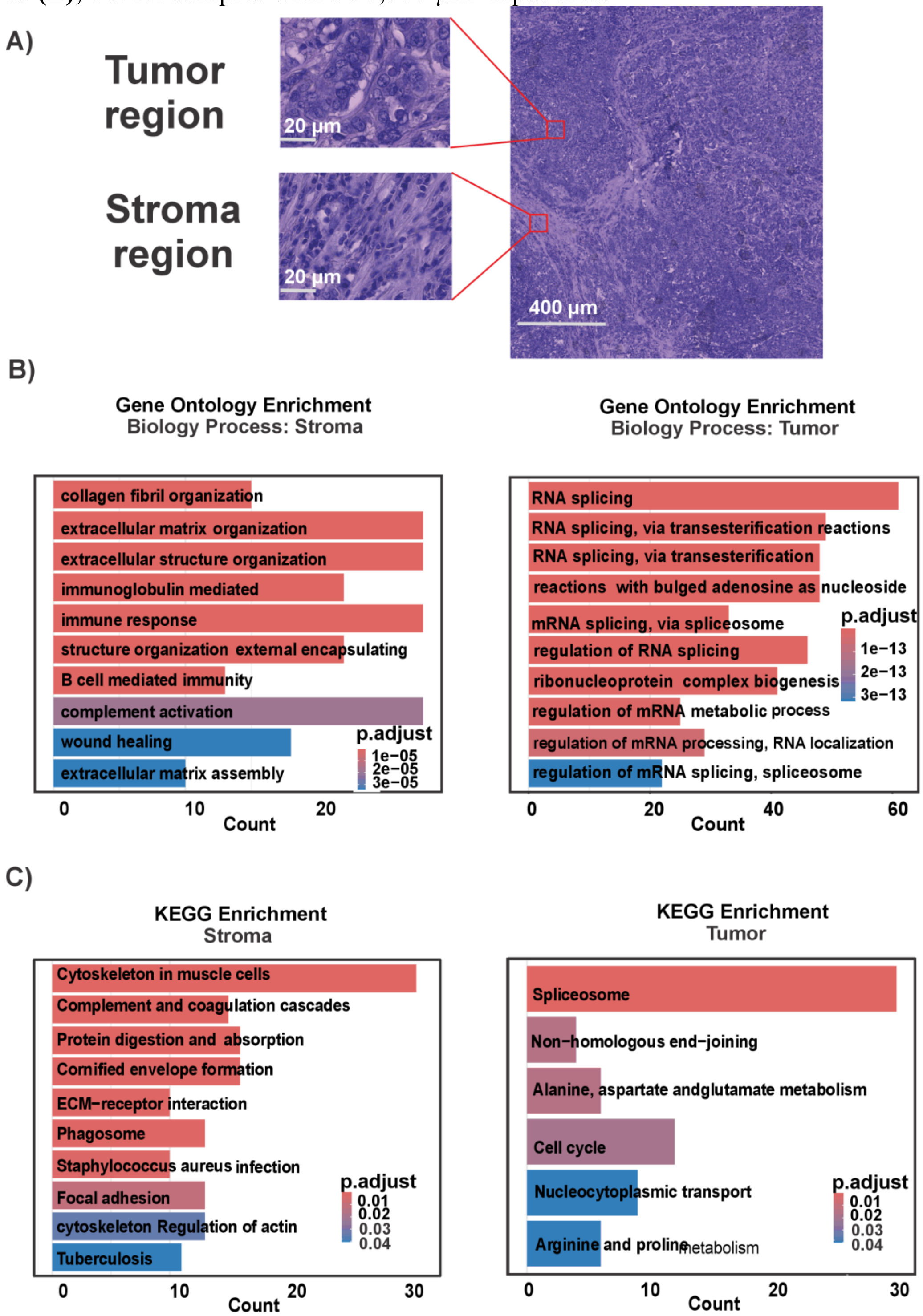

Supplementary Fig. 6. Spatial proteomic differentiation of tumor and stromal regions in TNBC. (A) Representative annotated tissue sections showing cancer and stromal microdissected regions. **(B)** GO-term enrichment of Biological process (upper panel) and KEGG pathway enrichment analysis of stromal regions (lower panel). **(C)** GO-term enrichment of Biological process (upper panel) and KEGG pathway enrichment analysis of tumor regions.

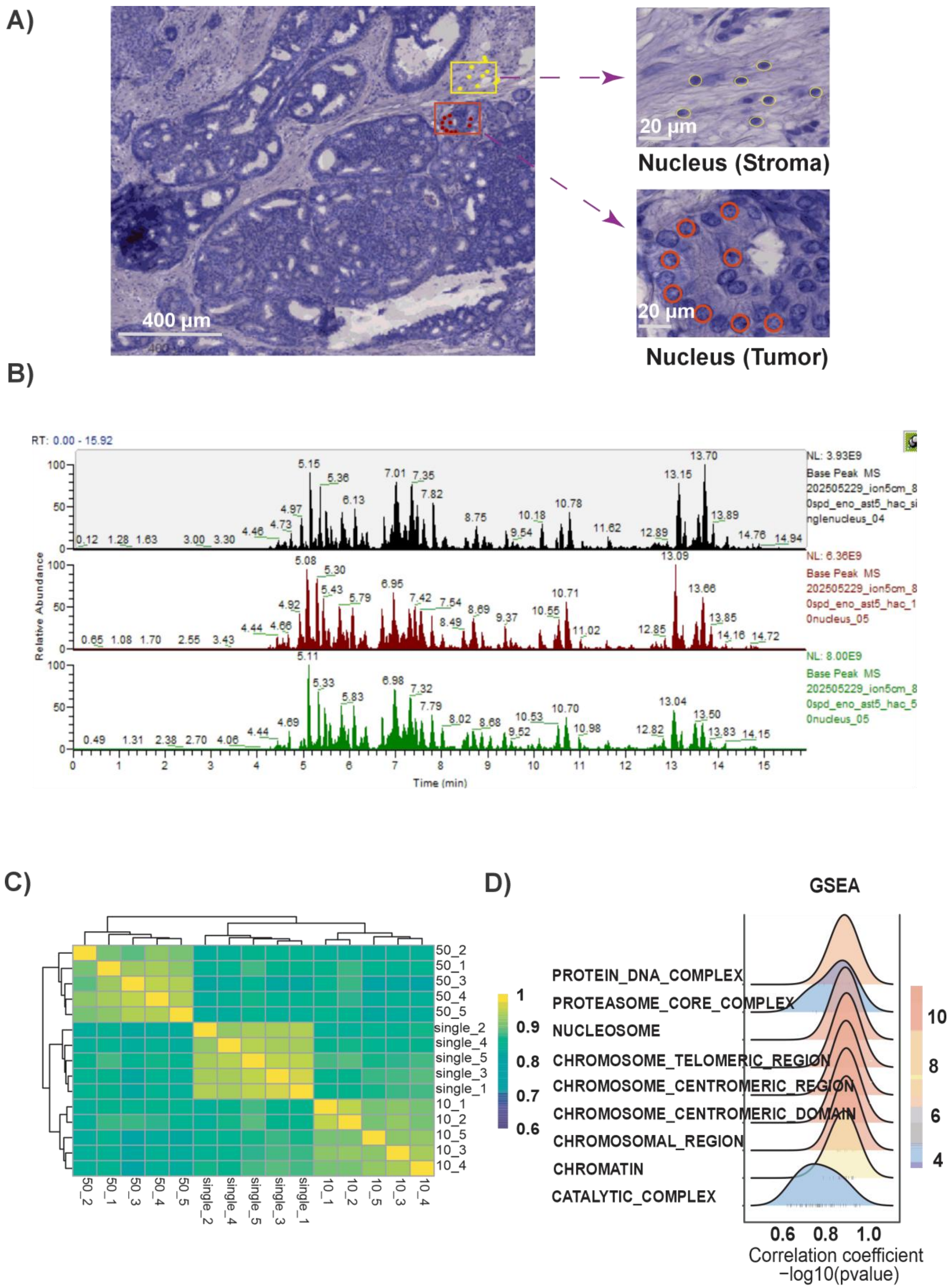

Supplementary Fig. 7. Subcellular spatial proteomics of nuclear-enriched micro-regions. (A) Microdissected nuclear-enriched regions (~60 µm²) from tumor and stromal compartments. **(B)** Representative base peak chromatograms showing peptide signal scaling across input levels of 1, 10, and 50 nuclei. **C)** Pearson correlation and clustering analyses

reflect consistent quantitative proteome profiling across biological replicates. **(D)** Gene set enrichment analysis (GSEA) enrichment analysis of nuclear enriched microdissected samples.

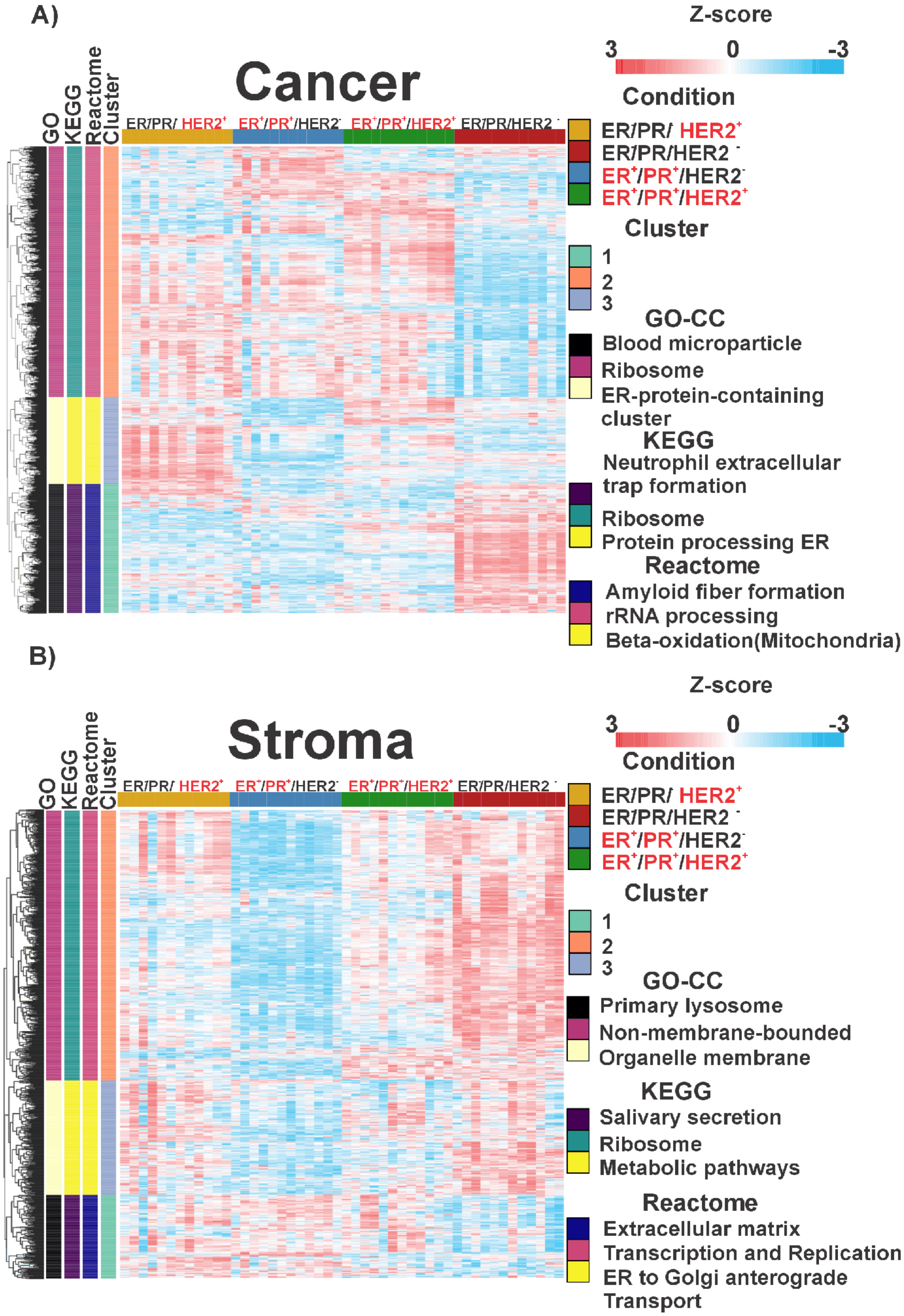

Supplementary Fig. 8. Spatial proteomic signatures across breast cancer molecular subtypes. (A) Hierarchical clustering of differentially expressed protein groups across ER$^-$/PR$^-$/HER2$^+$, ER$^+$/PR$^+$/HER2$^+$, ER$^+$/PR$^+$/HER2$^-$, and triple-negative breast cancer

subtypes, visualized independently for tumor (upper) and stromal (lower) compartments (n = 5 biological replicates; (two-way ANOVA; FDR 0.05).

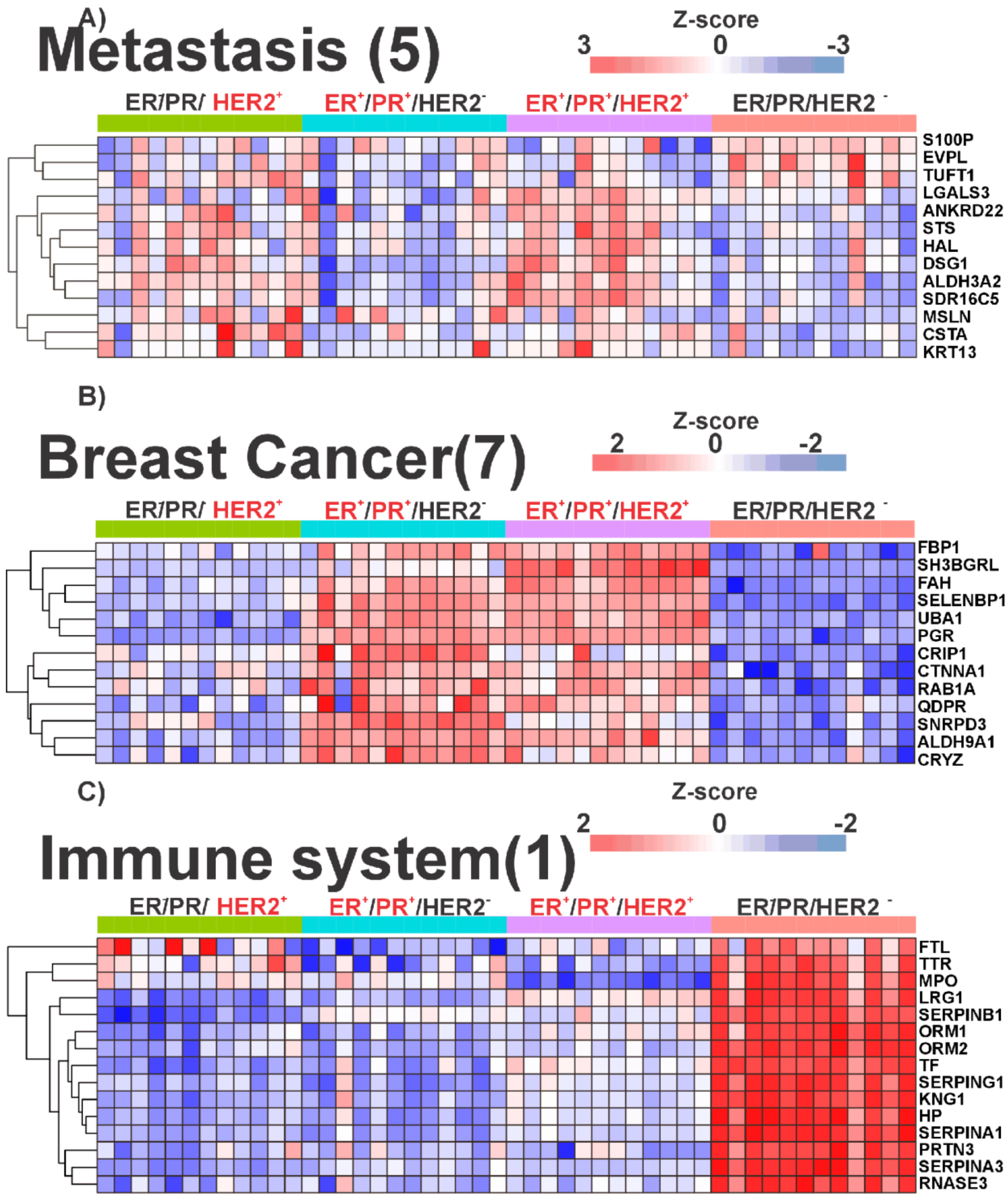

Supplementary Fig. 9. Spatial proteomic modular signatures across breast cancer subtypes.

Hierarchical clustering of protein groups identified as differentially expressed by ANOVA across ER⁻/PR⁻/HER2⁺, ER⁺/PR⁺/HER2⁺, ER⁺/PR⁺/HER2⁻, and triple-negative breast cancer subtypes, shown for distinct functional modules: (A) metastasis-associated proteins.

(B) breast cancer–associated signaling programs. (C) immune-related signatures. The enriched derived cluster is shown in parentheses. Data are displayed separately by tumor subtype (n = 5 biological replicates; two-way ANOVA, FDR < 0.05).

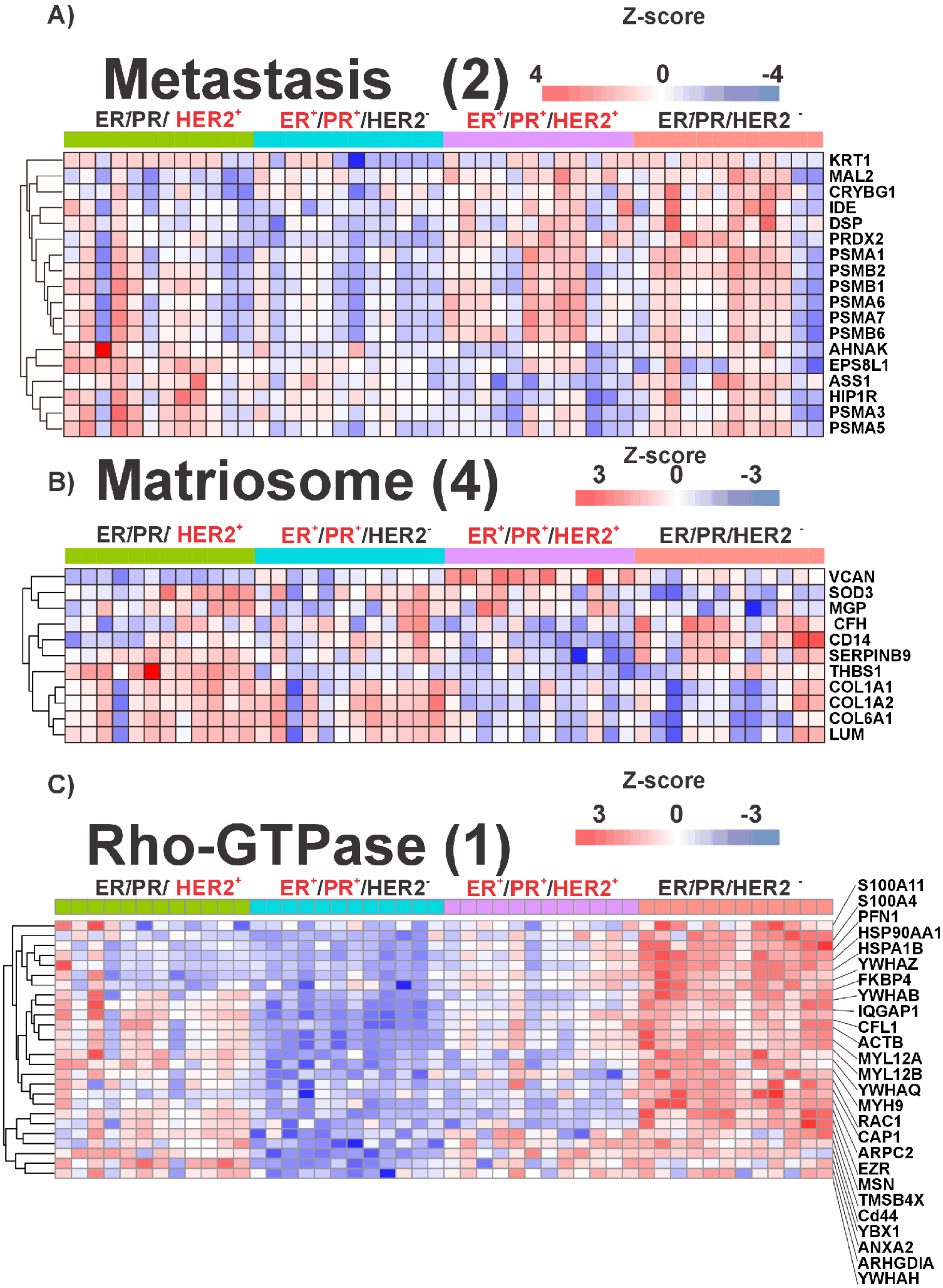

Supplementary Fig. 10. Spatial proteomic modular signatures across breast cancer stromal subtypes. Hierarchical clustering of protein groups identified as differentially expressed by ANOVA across $ER^-/PR^-/HER2^+$, $ER^+/PR^+/HER2^+$, $ER^+/PR^+/HER2^-$, and triple-negative breast cancer stromal subtypes, shown for distinct functional modules: (A) metastasis-associated proteins. (B) Matriosome–associated signaling programs. (C) Rho-

GTPase-related signatures. The enriched derived cluster is shown in parentheses. Data are displayed separately by stromal tumor subtype (n = 5 biological replicates; two-way ANOVA, FDR < 0.05).

Supplementary Note 1

Spatial Hanging-Droplet Proteomics: a Laser-Microdissection Workflow for Region-Resolved Sample Preparation on the EVO 96 Chip

This protocol describes an integrated, low-input workflow for spatial proteomics that couples laser-microdissection (LMD) of histological regions of interest with hanging-droplet sample processing on a 96-well EVO 96 chip. Tissue regions are excised by LMD directly into individually addressable chip wells, decrosslinked and lysed using a sealed hanging-droplet configuration that suppresses evaporation, digested with trypsin/Lys-C, and desalted on Evotips for direct injection into liquid chromatography–tandem mass spectrometry (LC–MS/MS). The workflow supports both rapid, standard-shape sampling for method optimization and precise, large-format regional sampling for biological studies, and is compatible with single-cell- to micro-scale protein inputs. The complete procedure, from chip loading to Evotip storage, requires approximately 120 minutes of hands-on and incubation time, excluding LC–MS/MS analysis.

Limitations

Successful microdissection depends critically on tissue-section dryness and laser-parameter optimization, both of which are tissue- and instrument-specific and may require preliminary calibration (see Critical steps, Step 8). The hanging-droplet seal is sensitive to plate choice and temperature; substitution of the specified Armadillo plate or omission of plate pre-chilling is not recommended without re-validating seal integrity. The protocol has been optimized for the EVO 96 chip and Leica LMD7000; adaptation to other LMD platforms will require redesign of the collection adapter (Step 2).

Experimental design

The workflow is organized into four stages: (i) chip and adapter preparation and loading into the LMD instrument (Steps 1–2); (ii) calibration and laser microdissection (Steps 3–8); (iii) hanging-droplet decrosslinking, lysis and digestion (Steps 9–17); and (iv) acidification and Evotip-based desalting (Steps 18–19). A key branch point occurs at Step 9, where the timing of TEAB buffer addition relative to LMD is chosen according to whether rapid, standard-shape collection or slower, precise regional collection is being performed.

Level of expertise needed

This protocol assumes familiarity with laser-microdissection microscopy and standard proteomic sample-preparation techniques. Prior experience operating a Leica LMD7000 (or equivalent) is recommended.

Materials

Biological materials

Fresh-frozen or FFPE tissue sections mounted on LMD-compatible membrane slides, prepared according to the standard histology protocol used in the originating laboratory (see accompanying Materials and Methods for tissue-specific preparation details).

Reagents

Reagent	Notes
Triethylammonium bicarbonate (TEAB), 25 mM	Lysis/decrosslinking buffer; prepare fresh (see Reagent Setup)
Trypsin/Lys-C mix, MS grade	5 ng/µL working solution for single-cell-scale inputs; 10 ng/µL for micro-scale inputs
Formic acid (FA), 10% (v/v)	For quenching after digestion
Acetonitrile (ACN)	Component of Evotip solvent B
Solvent B: ACN with 0.1% FA	Evotip conditioning
Solvent A: 0.1% FA in water	Evotip equilibration and washing
1-Propanol	Evotip wetting
Milli-Q water (LC–MS grade)	Evotip storage/hydration
pH indicator paper (range ~2–10)	In-process pH verification

▲ **CRITICAL STEP** All buffers should be prepared using LC–MS-grade water and freshly diluted where indicated; residual salts or contaminants at these sub-microliter volumes disproportionately affect downstream MS sensitivity.

Equipment

Equipment	Notes
EVO 96 chip	96-well microfabricated collection chip; catalog number and supplier as listed in the source Reagents and Tools table (Materials and Methods)
Custom 3D-printed LMD adapter	Design parameters obtained from the Fabian Coscia laboratory (see Materials and Methods); required because the EVO 96 chip does not fit directly into the LMD collection holder
Standard 96-well plate holder	Houses the assembled adapter–chip unit inside the LMD instrument
Leica LMD7000 laser-microdissection microscope (or equivalent)	For region-of-interest excision and collection
Armadillo 96-well PCR plate (Thermo Fisher)	Selected for its tight seal with the EVO 96 chip in the hanging-droplet configuration
Heating block (e.g., Eppendorf ThermoMixer, static mode)	For heat-induced decrosslinking/lysis at 90 °C
Standard laboratory incubator	For 37 °C tryptic digestion
Plate centrifuge, capable of 800 × g	For Evotip conditioning and peptide loading
Evotips (Evosep)	C18 desalting tips for direct LC–MS/MS loading
Single- and multi-channel pipettes (µL range)	For buffer, enzyme and acid addition

Equipment	Notes
–20 °C freezer	For plate pre-chilling and optional sample storage after acidification
4 °C refrigerator	For short-term Evotip storage prior to LC–MS/MS

Reagent setup

- 25 mM TEAB. Dilute from concentrated stock in LC–MS-grade water immediately before use; do not store diluted working solution beyond the day of preparation.
- Trypsin/Lys-C working solution. Reconstitute and dilute to 5 ng/µL (single-cell scale) or 10 ng/µL (micro-scale) in the appropriate digestion buffer immediately before Step 12; keep on ice until use.
- 10% formic acid. Prepare from concentrated FA in LC–MS-grade water; store at room temperature, protected from light.
- Evotip solvent A (0.1% FA) and solvent B (ACN, 0.1% FA). Prepare fresh and store at room temperature for up to 1 week.

Equipment setup

- Pre-chill the Armadillo 96-well plate at –20 °C for at least 30 min before hanging-droplet assembly (Steps 9 and 13); this improves the seal between the plate and the EVO 96 chip.
- 3D-print and assemble the custom LMD adapter according to the Cosias Fabian laboratory design files before the first use of the workflow; verify a tight fit between the EVO 96 chip and the adapter before proceeding to Step 2.
- Set the heating block to 90 °C and confirm static (0 rpm) operation before Step 14; remove any lid or cover from the heating block, as a closed lid disrupts the hanging-droplet seal.

Procedure

Stage 1: EVO 96 chip and adapter preparation • Steps 1–2

1| Retrieve an EVO 96 chip (C-PEVO-96-10, Cellenion).

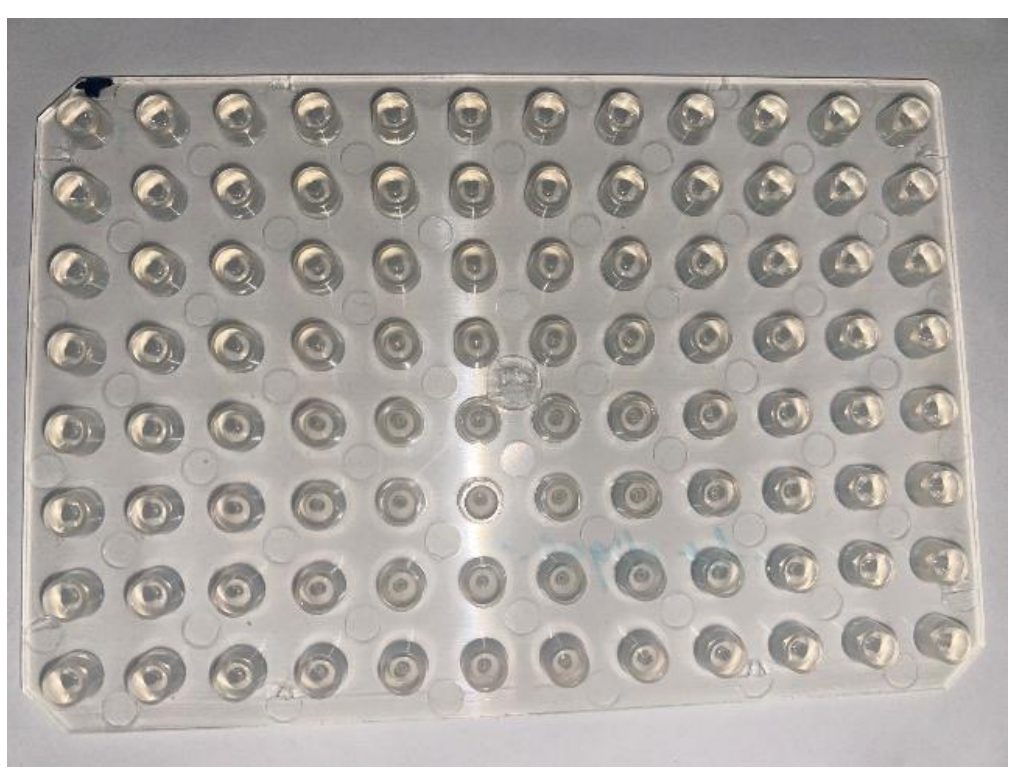

Figure 1. *The EVO 96 chip used for laser-microdissection sample collection.*

2| Fit the EVO 96 chip tightly into the custom 3D-printed adapter, then insert the assembled adapter–chip unit into the standard 96-well plate holder. Load the holder assembly into the LMD instrument.

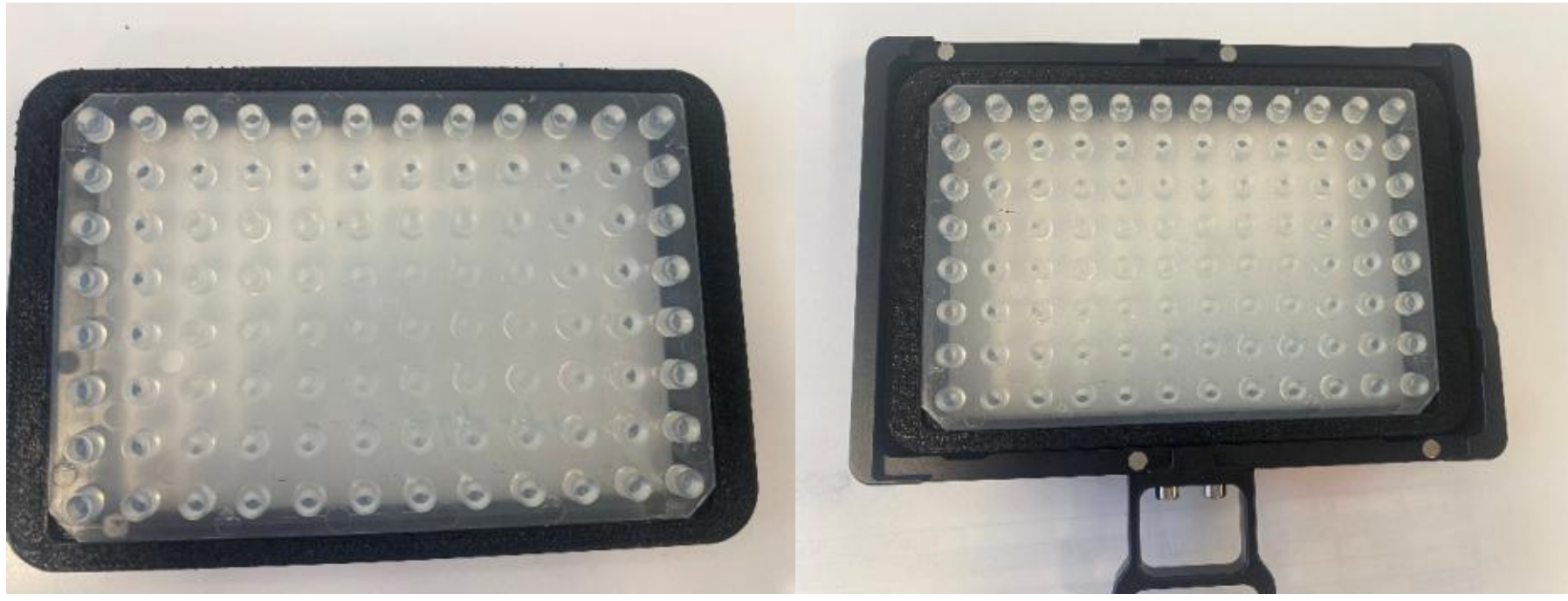

Figure 2. *The EVO 96 chip fitted into the custom 3D-printed adapter (left) and the assembled adapter–chip unit (right).*

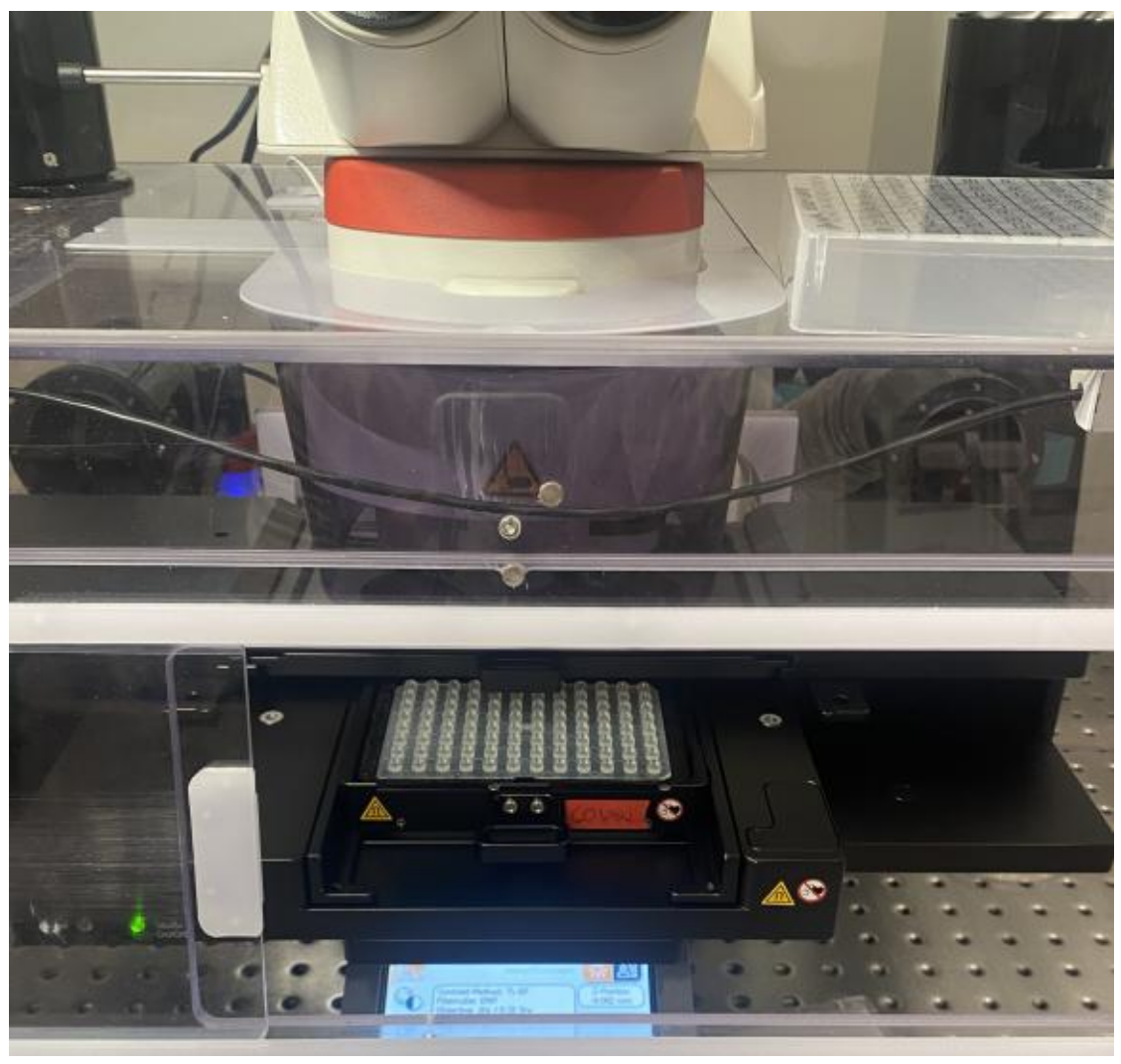

Figure 3. *The adapter–chip unit seated in the standard 96-well plate holder, ready for loading into the LMD instrument.*

⏱ ***TIMING*** *Steps 1–2, ~5 min*

Stage 2: Calibration and laser microdissection • Steps 3–8

3| Perform position calibration to confirm that the laser aligns correctly with each chip well, so that dissected material falls reliably into the designated well.

4| Perform cutting-accuracy calibration to confirm precise excision of the intended target regions.

5| Set laser capture parameters (intensity, aperture, speed and focus) as described in the Laser Capture subsection of the Materials and Methods.

6| Before loading the tissue slide, heat it at 37 °C for 20–30 min to achieve the sample dryness required for clean microdissection.

> **▲ CRITICAL STEP** Sample dryness is, together with laser-parameter optimization, the single most important determinant of successful microdissection. Under- or over-dried sections produce ragged cuts or poor detachment.

7| Before starting collection, mark the top-left or top-right corner of the chip with a visible label.

> **▲ CRITICAL STEP** Sample position must be tracked relative to the tissue for spatial proteomics. The chip will be inverted in Step 9, so orientation marking at this stage is essential for downstream reconstruction of sample identity.

8| Perform laser microdissection, collecting the target regions into their designated EVO 96 chip wells.

> **? TROUBLESHOOTING** If dissected fragments fail to land in the intended well, repeat position calibration (Step 3) and confirm the slide is correctly seated and the section is adequately dry (Step 6).

⏱ ***TIMING*** *Steps 3–8, 20–30 min for rapid/standard-shape collection; >30 min for precise regional or large-yield collection, depending on the number and complexity of target regions*

Stage 3: Decrosslinking and cell lysis • Steps 9–12

The timing of TEAB buffer addition depends on the collection strategy used in Stage 2. Follow option A for rapid, standard-shape collection (≤30 min total LMD time) or option B for precise regional or large-yield collection (>30 min total LMD time).

9| **Add 25 mM TEAB buffer, choosing option A or B:**

(A) Rapid workflow optimization (≤ 30 min collection). Pre-load 13–14 µL of 25 mM TEAB into each designated well using a single- or multi-channel pipette before LMD begins (i.e., before Step 8). Because the entire 96-well plate is processed within 20–30 min, evaporative loss is negligible.

(B) Precise regional selection or large sample yield (> 30 min collection). Do not pre-load buffer. Instead, add 13–14 µL of 25 mM TEAB to each well immediately after LMD collection is complete (after Step 8), to avoid the substantial evaporative loss that occurs over prolonged collection times.

10| Invert the EVO 96 chip and place it onto a pre-chilled Armadillo 96-well plate to form the hanging-droplet configuration (see Equipment setup). Ensure a tight seal by using the Armadillo plate and confirming it has been pre-chilled at –20 °C.

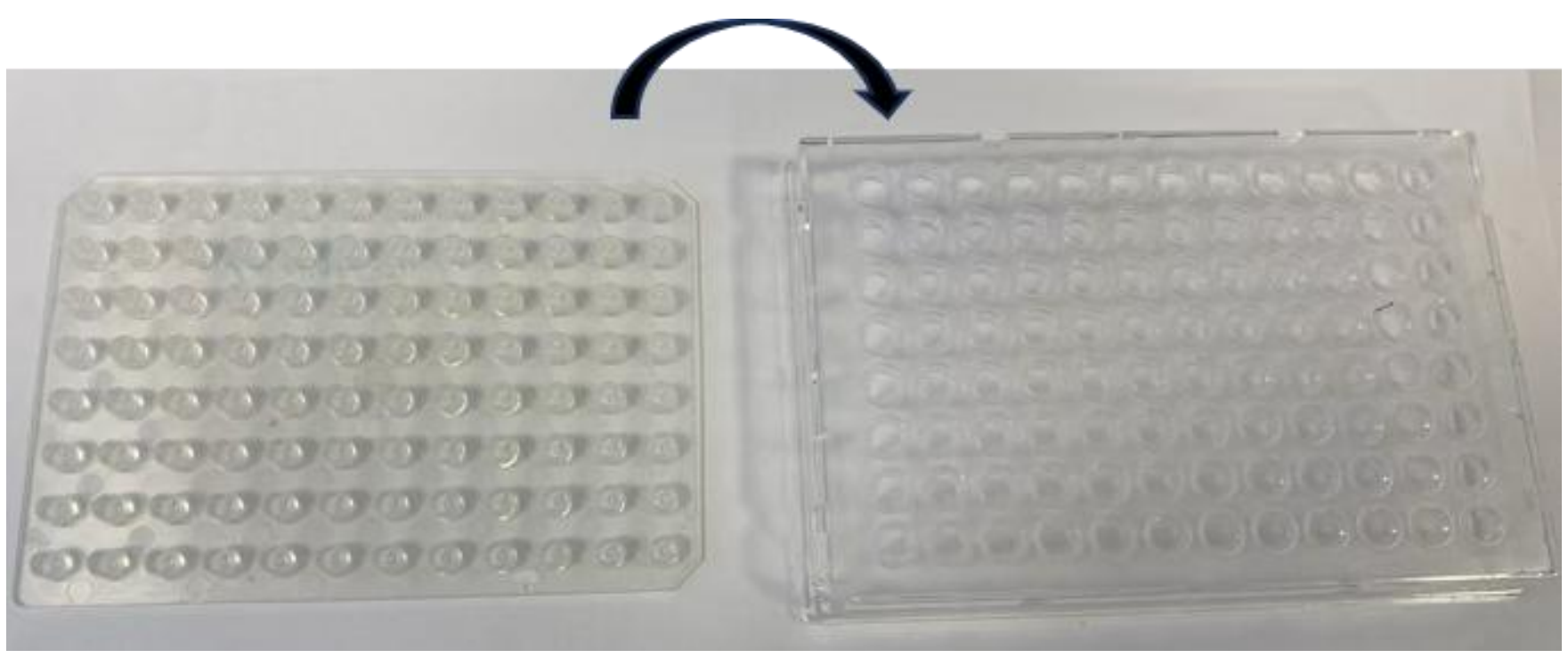

Figure 4. *Schematic of the hanging-droplet configuration: inversion of the EVO 96 chip onto the Armadillo 96-well plate suspends each sample droplet and limits evaporation (compare Supplementary Figure 1A).*

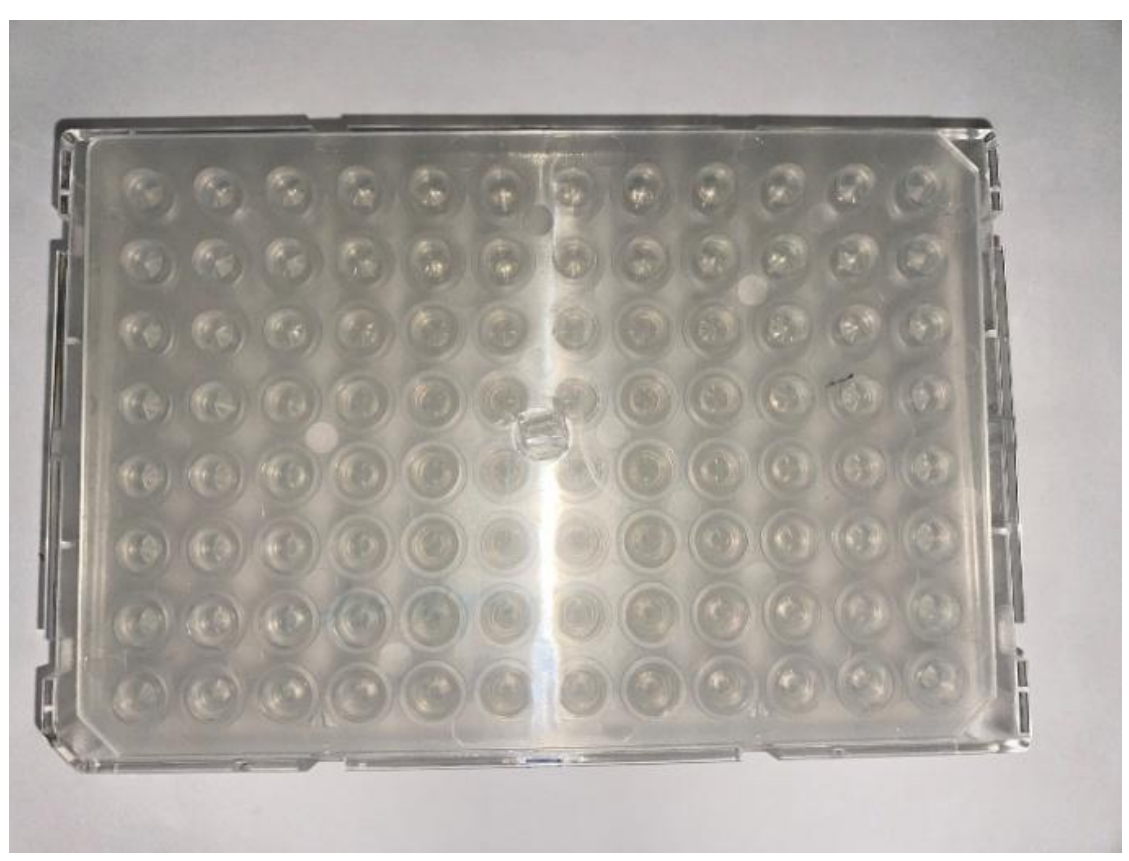

Figure 5. *The Armadillo 96-well plate used as the sealing base for the inverted EVO 96 chip.*

▲ **CRITICAL STEP** Seal integrity between the EVO 96 chip and the Armadillo plate is the primary determinant of hanging-droplet performance. Use only the Armadillo plate (not alternative 96-well formats) and pre-chill it at –20 °C before assembly.

11| Place the assembled Armadillo plate with the inverted EVO 96 chip onto a heating block pre-set to 90 °C.

12| Incubate for 40 min under static conditions (0 rpm).

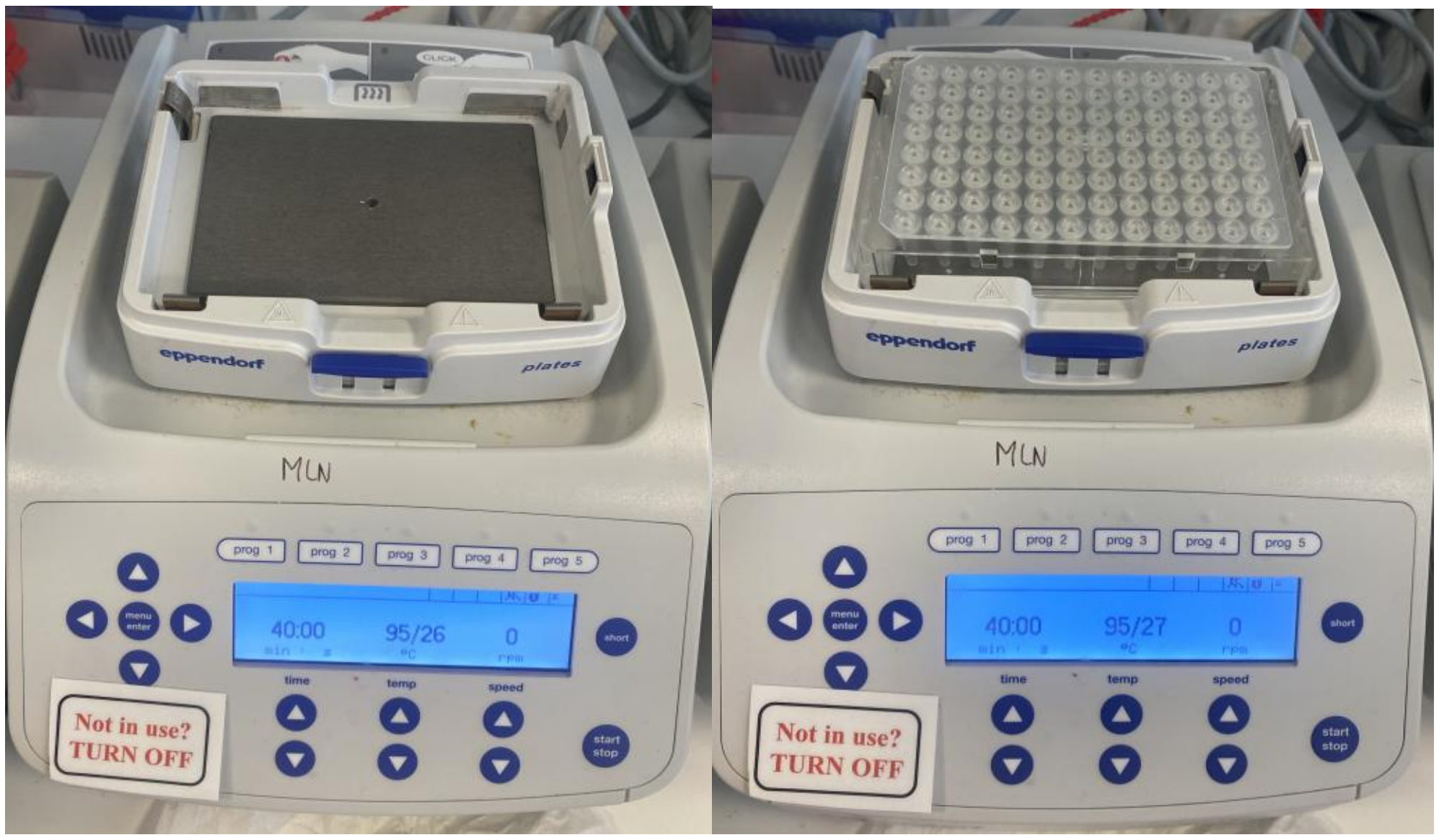

Figure 6. *Heat-induced decrosslinking and lysis: the sealed Armadillo plate–EVO 96 chip assembly on the heating block, incubated at 90 °C, 40 min, 0 rpm.*

▲ **CRITICAL STEP** Do not place a lid on the heating block. A lid interferes with the hanging-droplet principle and will compromise the seal. Optionally, place a weight or other flat object on top of the EVO 96 chip to improve seal tightness.

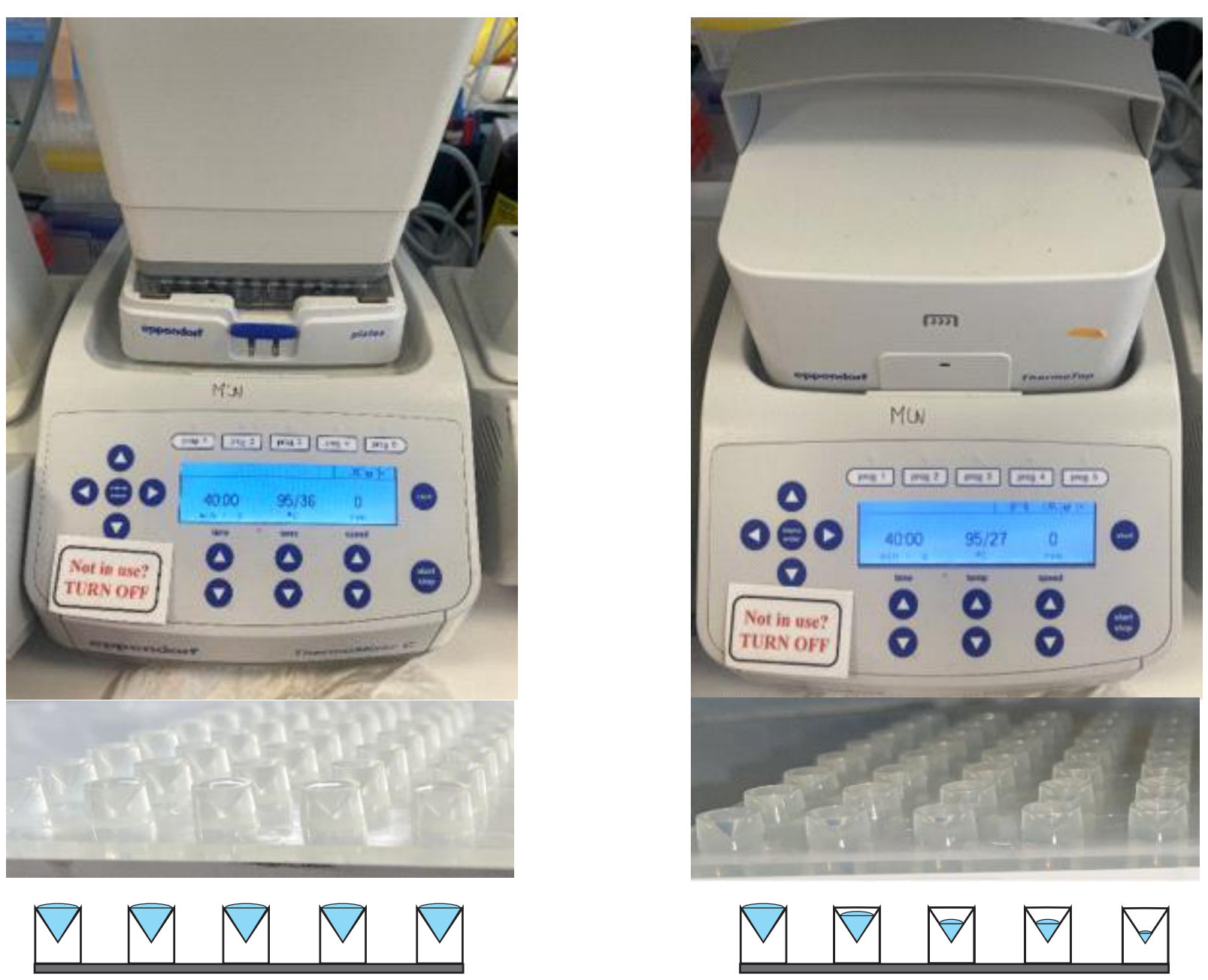

Figure 7. *Representative outcomes after decrosslinking: a correctly sealed, intact hanging droplet (left, ✓) versus a failed seal with visible droplet loss (right, ✗).*

? TROUBLESHOOTING If droplets are lost or wells appear dry after the 90 °C incubation (as in the failure case shown in Figure 7), check that the Armadillo plate was adequately pre-chilled, confirm the heating block lid was removed, and consider adding a weight on top of the chip to improve seal tightness.

⏱ ***TIMING*** *Steps 9–11, ~45–50 min (5–10 min setup + 40 min incubation)*

Stage 4: Digestion • Steps 13–16

13| Verify the pH of the sample using pH paper; it should be approximately 8, which is optimal for tryptic digestion.

? TROUBLESHOOTING If pH is substantially below 8, prepare fresh 25 mM TEAB (buffer degrades over time) and repeat buffer addition.

14| Add the trypsin/Lys-C enzyme mix: 2.5 µL of 5 ng/µL mix per well for single-cell-scale experiments, or 2.5 µL of 10 ng/µL mix per well for micro-scale experiments.

15| Repeat the hanging-droplet inversion and sealing procedure described in Step 10 to reassemble the chip on a (freshly pre-chilled) Armadillo plate.

16| Place the assembled plate into a standard incubator and incubate at 37 °C for 1 h to allow complete protein digestion.

⏱ ***TIMING*** *Steps 12–15, ~1 h 10 min*

Stage 5: Digestion quenching and desalting • Steps 17–25

17| Add 1 µL of 10% formic acid to each well to quench digestion. Verify the resulting pH using pH paper; it should read approximately 2–3 for efficient peptide binding during desalting.

■ PAUSE POINT After acidification, the Armadillo plate with the EVO 96 chip can be stored at –20 °C until further processing.

18| Rinse Evotips with 20 µL solvent B (ACN, 0.1% FA) and centrifuge at 800 × g for 1 min.

19| Soak the Evotips in 1-propanol until fully wet.

20| Equilibrate the Evotips with 20 µL solvent A (0.1% FA) and centrifuge at 800 × g for 1 min.

21| Load the acidified peptide samples from the EVO 96 plate onto the wetted Evotips and centrifuge at 800 × g for 1 min to bind peptides to the C18 resin.

22| Wash the loaded Evotips with 20 µL solvent A and centrifuge at 800 × g for 1 min.

23| Add 100–200 µL solvent A to each tip and centrifuge at 800 × g for 10 s to keep the resin hydrated.

24| Add Milli-Q water to the Evotip storage box to keep the tips wet.

25| Store the prepared Evotips at 4 °C until LC–MS/MS analysis.

■ PAUSE POINT Prepared Evotips are stable at 4 °C for short-term storage prior to LC–MS/MS injection; process within the timeframe validated in your laboratory to avoid peptide loss or oxidation.

*⏱ **TIMING** Steps 16–24, ~20–25 min hands-on time*

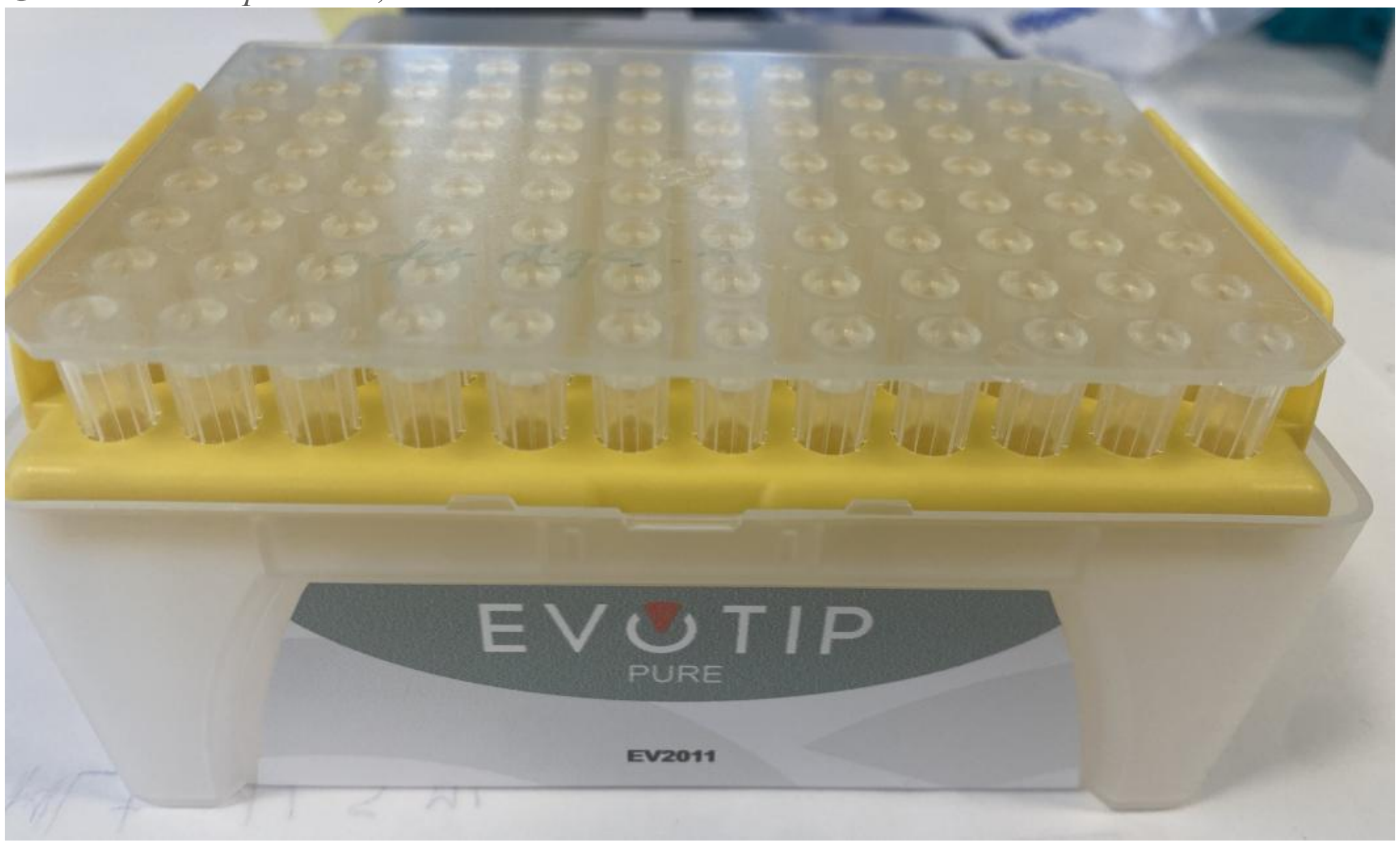

Figure 8. *Loading of acidified peptide samples from the EVO 96 plate onto wetted Evotips prior to centrifugation.*

Troubleshooting

Problem (step)	Possible cause	Solution
Dissected regions do not land in the designated well (Step 8)	Position calibration error	Repeat position calibration (Step 3); confirm chip/adapter is seated correctly in the holder
Ragged or incomplete cuts during microdissection (Step 8)	Section too wet or too dry; suboptimal laser parameters	Re-optimize the 37 °C drying step (Step 6, 20–30 min) and revisit laser intensity/aperture/speed/focus settings
Excessive evaporation / low residual volume after LMD (Stage 2–3)	Buffer pre-loaded but collection exceeded 30 min	Switch to post-collection buffer addition (Step 9, option B) for future runs; do not pre-load buffer for long or complex collections
Hanging droplet collapses or leaks during 90 °C incubation (Step 11)	Inadequate seal between chip and plate	Confirm use of the Armadillo plate, pre-chill at –20 °C before assembly, remove any heating-block lid, and add a weight on top of the chip
Sample pH not ~8 before enzyme addition (Step 12)	TEAB buffer degraded or diluted incorrectly	Prepare fresh 25 mM TEAB and repeat buffer addition/quantification
Sample pH not ~2–3 after acidification (Step 16)	Insufficient formic acid or pipetting error	Re-verify with fresh pH paper; add additional 10% FA in 0.5 µL increments until pH 2–3 is reached
Low peptide yield after Evotip desalting (Steps 17–24)	Incomplete binding, tip not fully wetted, or resin dried out	Confirm 1-propanol wetting step is complete (Step 18) and that resin hydration (Step 22) is performed promptly after washing

Timing

- Steps 1–2 (chip and adapter preparation): ~5 min
- Steps 3–8 (calibration and laser microdissection): 20 min to >30 min, dependent on collection strategy
- Steps 9–12 (buffer addition, hanging-droplet assembly, decrosslinking/lysis): ~40 min
- Steps 13–16 (digestion): ~ 60 min
- Steps 17–25 (acidification and Evotip desalting): ~20 min
- Total hands-on sample preparation (~120 min) plus Evotip loading protocol: ~ 150 min, excluding LC–MS/MS analysis

Anticipated results

A successfully executed workflow yields intact, correctly sealed hanging droplets throughout decrosslinking and digestion (Figure 7, left), with negligible evaporative loss, and Evotips loaded with acidified tryptic peptides ready for direct LC–MS/MS injection. Samples processed at single-cell to micro-scale input should be evaluable by standard LC–MS/MS quality metrics (e.g., number of protein groups identified, chromatographic peak shape) consistent with the sensitivity of the mass spectrometry platform used; refer to the associated Materials and Methods and results sections for platform-specific benchmarks.

.
