# Supplementary material for: High-throughput spatial proteomics from cellular to subcellular scales via a hanging droplet workflow
Source: Nat Commun. 2026 Oct 9;17:11488. doi: 10.1038/s41467-026-78415-x (PMC13740325; doi:10.1038/s41467-026-78415-x)
Supplement: Supplementary file 3 [file 41467_2026_78415_MOESM4_ESM.pdf]

nature portfolio

Peer Review File

High-Throughput Spatial Proteomics from Cellular to Subcellular Scales via a Hanging Droplet Workflow

Corresponding Author: Professor Jesper Olsen

This file contains all reviewer reports in order by version, followed by all author rebuttals in order by version.

Version 0:

Reviewer comments:

Reviewer #1

(Remarks to the Author)
In this manuscript, the authors describe a hanging droplet-based, EVO 96-compatible sample preparation workflow for laser capture microdissection (LCM)-based spatial proteomics of minute FFPE tissue regions. The proposed method aims to streamline the processing of small tissue inputs, by integrating LCM, decrosslinking, digestion, and DIA-MS analysis.

The reviewer appreciates the authors' efforts to address technical challenges associated with spatial proteomics of FFPE samples and minute LCM regions. The study demonstrates technically competent execution and presents several application examples, including subcellular-scale analyses. However, the overall methodological advance appears modest. Similar LCM-based spatial proteomics workflows for FFPE tissues and small sample inputs have been reported previously, and the present work does not clearly establish a substantial advantage in terms of spatial resolution, proteome depth, or throughput, despite the use of state-of-the-art instrumentation.

If positioned as a methodology-focused study, the workflow would benefit from clearer articulation of novelty relative to recent literature, more rigorous benchmarking against established approaches, and substantially expanded methodological descriptions. These aspects are critical for evaluating the significance and reproducibility of the proposed method at the level expected for a Nature Communications publication.

Major Comments
1. The opening statement of the Abstract does not convincingly define a current methodological gap. Multiple state-of-the-art spatial proteomics approaches have already addressed FFPE samples and minute regions, including subcellular-scale analyses (e.g., Deep Visual Proteomics, and other related methods; PMIDs: 37909047, 35590073, 27062885, 40240610, 39477916, etc.). Moreover, challenges such as evaporation during decrosslinking and low throughput are not universal limitations across existing workflows.

Notably, a recent study from the Coscia group (PMID: 41418981) reports a standardized LCM-based spatial proteomics workflow that integrates EVO 96 with further loss-reduced tissue processing using CellenONE, achieving high throughput and robust proteome depth from small FFPE regions. Given that the present study relies on a similar multiwell-based peptide preparation strategy, the novelty of the proposed workflow is not immediately apparent. In this context, the reported throughput of ~80 samples per day does not exceed the ~100 samples per day reported by the Coscia group using a comparable workflow with similar sample sizes and proteome depth. The authors should therefore clearly articulate what specific methodological advance is introduced beyond existing approaches.
2. The Introduction is insufficiently developed and does not comprehensively cover recent advances in spatial proteomics of FFPE and minute samples. Several key developments are missing or under-referenced. For example, Deep Visual Proteomics is discussed primarily via an early 2022 publication, while more recent work from the Mann group and other laboratories, some of which address closely related technical challenges, are not adequately considered. A more balanced and up-to-date overview is required to properly position the present work within the current literature.
3. To justify the use of a hanging droplet strategy for FFPE decrosslinking and digestion, the authors should perform more comprehensive benchmarking against established workflows from the other groups, like the Mann and Coscia groups. Currently, comparisons are made primarily to the One-Tip method, which was originally developed for cultured single-cell proteomics and is not an appropriate reference for FFPE minute-sample preparation. Without direct comparison to state-of-the-art FFPE LCM workflows, it is difficult to assess whether the proposed approach provides meaningful advantages.

4. The authors state that n-dodecyl-β-D-maltoside (DDM) was mainly introduced to prevent protein adsorption to surfaces. However, this is not the primary rationale for DDM usage in the proteomics community. A previous study (PMID: 33649493), which is widely cited and adopted, demonstrates that DDM can also improve enzymatic digestion efficiency and overall proteome recovery when applied under well-defined conditions. In contrast, the present study reports fewer protein identifications in the presence of DDM, which is opposite to the published observations. However, the manuscript does not provide additional analyses to clarify the underlying reasons for this discrepancy, such as assessments of missed cleavage rates, digestion efficiency, or changes in protein class composition (e.g., membrane-associated versus soluble proteins). Moreover, the Materials and Methods section lacks critical details regarding DDM usage, including its concentration and timing of addition. Given that DDM concentration is known to be a key determinant of its performance, the lack of methodological transparency makes it difficult to evaluate the results or assess reproducibility.
5. The Materials and Methods section is overly brief and appears incomplete. Beyond limited descriptions of sample preparation, key details related to downstream statistical analysis and bioinformatics processing are missing or unclear. This lack of detail further limits the ability to critically evaluate the robustness and reproducibility of the reported results.
6. Figure 1a is poorly presented and contains multiple issues, including misaligned graphical elements, apparent design errors (e.g., stray crossing lines), and inappropriate labeling (e.g., numbering within "Section V"). In addition, the inclusion of standard H&E staining as a major component of the workflow diagram is not acceptable, as it is neither novel nor a defining technical element of the proposed method. Overall, the figure is difficult to follow and requires substantial revision to clearly convey the workflow.
7. The Discussion session does not contain a balanced positioning of the method, and comparison with the state of the art methods in the literature.
Minor Comments
1. The title may be overly broad. Including both "cellular" and "subcellular" resolution may be unnecessary, as achieving subcellular resolution inherently implies cellular resolution. A more concise title may be preferable.
2. The manuscript formatting is inconsistent and appears to retain elements from a previous submission, including a "Science Advances" footer. Furthermore, inconsistencies in layout, spacing, and section formatting affect the readability and overall professionalism of the manuscript. All formatting issues and remnants from previous submissions should be carefully corrected.
3. The authors state that "2.5 µL of a 10 ng/µL trypsin and Lys-C mix" was used for digestion (25 ng enzyme). It is unclear for which sample input this amount was applied. For very small samples (e.g., a few cells or isolated nuclei), this enzyme amount appears excessive and should be justified.
4. The PRIDE raw data are provided as individual files spanning multiple acquisition dates, making it difficult to identify experimental batches and corresponding conditions. Grouping raw files from the same experiment into clearly annotated batches (e.g., via folders or a short README file) would substantially improve data organization and reuse.

Reviewer #2

(Remarks to the Author)
Chen et. al. described a sample preparation method for spatial proteomics of FFPE tissue. They integrated the approach with Evo96 chip, Evosep LC, and Astral MS with DIA analysis. They evaluated the approach's sensitivity and confirmed that it is consistent with existing technologies. They also applied to study proteomic differences between stromal and tumor regions, and the DE proteins were consistent with cancer biology. While it is a viable approach to improve the throughput of spatial proteomics, the reviewer is not convinced that such incremental improvement justifies publication in Nature Communications.

Main comments:

(1) The improvement of the hanging drop approach is mainly to facilitate high-temperature de-crosslinking in a small volume. The data shown in Figure 1 did indicates the improvement. However, they are using non-HIER-processed FFPE tissue. In most DVP or spatial proteomics, the tissues are pre-processed with HIER (high temperature epitope retrieval). Many groups (including my own lab) understand that the on-slide HIER is sufficient to de-crosslink proteins for spatial proteomics. Thus, the presented approach seems redundant, unless there is a case where tissue can not be HIER-processed. Note HIER is easy to perform and widely adopted in pathology labs.

The use of EVO96 chip for spatial proteomics is not new. Many papers have been published in the last several years. The work actually used a lot of protocol (Python script and 3D printed holders) from these published work.

(2) The author compared the new approach with the "on-tip" approach, developed and used in the authors' lab. Note on-tip approach is not a common way to do spatial proteomics due to the technical challenge of collecting the tissue from Leica LMD to the tip. I am wondering if the author can compare their approach with the most widely used DVP approach, and use the HIER process tissue?

(3) One of the biggest concerns is the lack of blank control samples when the authors present the proteome coverages of different tissue sizes. Because the laser dissection takes a long time and many air debris (protein contaminations) can get into the well. It is not clear how much contribution of the protein IDs is actually from dissected cells in addition to background contaminations. As such, the reviewer asks the author to include blank measurements from multiple empty sample prep wells on the same Evo96 chip

(4) Figure 2E, the author shows that the intensity distribution of different sizes is surprisingly similar, although the input

changes > 50X folds. It is a confusing result. Could you plot the intensities of commonly detected proteins across these different inputs?

(5) Figure 3A and B. The author indicates an average of 4500 proteins detected in the stromal and 5500 in the tumor. However, the Venn diagram only shows 2500-3200.

(6) Figure 1B, the author shows that TEAB gives better sensitivity over the ABC buffer. There is no additional discussion on the buffer. Did the results agree with previously published data? It is noteworthy that ABC is among the most widely used buffers for label-free proteomics, whereas TEAB is primarily used for TMT proteomics.

Reviewer #3

(Remarks to the Author)
Chen, Guzman, and Olsen present a high-throughput spatial proteomics workflow using a hanging droplet configuration on EVO96 chips for processing FFPE samples. The method achieves reasonable proteome depth (about 5500 protein groups from 250 cells) with a streamlined 2-hour sample preparation time. The introduction is well written, and the language throughout is measured and avoids oversellling. That's refreshing, thank you! However, several technical clarifications and controls will be helpful:

Major Comments
- The new method should add practicality to the field, however, a critical parameter missing from the manuscript is the time samples spend in the LMD instrument during collection. In our hands, a typical dissection protocol can take up to 18 hours for 96 wells. The authors should measure droplet volume over the duration of a typical collection run. This is essential to evaluate whether evaporation during collection undermines the advantages of the hanging droplet approach. In addition, the claim that DDM alters surface tension and increases evaporation (lines 151-153) would benefit from supporting data showing actual droplet volume reduction with DDM.
- The comparison to "on-tip" digestion (Fig. 1F-G) is unclear. Reading the methods (lines 448-464), both protocols appear to perform digestion in the EVO96 chip wells and the only apparent difference is using an incubator rather than the hanging configuration. This is not on-tip digestion as typically defined (e.g., the One-Tip method cited). The authors should either clarify the actual methodological difference or rename this comparison more accurately. Additionally, please confirm that the on-tip comparison used the same sample type and input amount.
- It is unclear whether replicates represent independent patients or multiple regions from single tissue blocks per subtype. If the latter, the statistical comparisons across breast cancer subtypes are not appropriately powered, and the claims need to be tempered accordingly. Please clarify the sample structure.
- The single-nucleus proteome depths (1200 PGs) are impressive but require a blank or negative control (same workflow, just no material cut into the well during LMD). That will be essential to demonstrate that identifications are not background signal.
- Please clarify whether match-between-runs (MBR) was used or files from different experimental setups were search together. I suggest to entirely separate experimental conditions during searching to avoid artificial inflation of identifications across conditions being compared.
- Great to see the data on PRIDE, could you please reorganize the data for usability? That includes search results as .tsv/.csv files (not everyone has Spectronaut), group raw files by experiment in zipped folders, and provide an overarching metadata file linking samples to experimental conditions. Also, the Data availability section states PXD071950 or so but the cover page has a different ID and credentials (that work for that ID). Please correct.

Minor Comments
a) Results/Methods:
- Use LMD consistently throughout (LCM typically refers to thermoplastic film capture)
- Please comment on the 15% missed cleavage rate, is this expected given the workflow?
- Supplementary Fig. 1A: specify optimal volume, show replicates with SD, clarify what the photo depicts
- Line 194: rephrase "was the primary source" to "was a major source", and consider "tissue architecture" instead of "spatial localization"
- Fig. 2B: "greater variability" rather than "greater biological heterogeneity" (technical contribution unknown)
- Describe tumor vs. stroma annotation procedure, was a pathologist involved?
- Report LMD sorting efficiency and dropout rates (we always have dropouts of 5-10%)
- Describe missing value handling and any batch correction in methods
- Be consistent with PR/PGR nomenclature throughout
- Missing reference at line 307 ("cancer cell invasiveness")
- Add statistical analysis details for functional enrichment (e.g., Supplementary Fig. 5)
- Fix duplicated H&E protocol text (lines 372-377)
- Text uses "crosslinking" where "decrosslinking" is meant in several places
- Reduce redundancy in Results section, particularly for Fig. 1
b) Figures:
- Unify font sizes and types across all figures; check for text/image overlap
- Fig. 1: fix "Ethonal" typo and clean up panel A composition
- Fig. 1G: add y-axis label
- Fig. 2E: add x and y axis labels
- Fig. 2F: add annotation within figure; clarify color gradient (z-score?)
- Fig. 5: dense and difficult to interpret; colors hard to distinguish

- Specify n in all figure legends
- Define error bars (SD) for all bar plots
- Add "z-score" label for color legends throughout
- Clarify protein labeling criteria in volcano plots, these seem random at the moment.
- Supplementary Fig. 1B: arrange before/after in left/right order
- Supplementary Fig. 1D: identify the red dot
- Supplementary Fig. 5: panel B not referenced in legend
- Supplementary Fig. 6: indicate which cluster from Fig. 5 these derive from

c) References:
- Makhmut et al. (MCP 2024) cited twice (refs 9 and 47)
- Missing citation for openDVP/Nimo et al., which was clearly used for the QuPath-to-LMD coordinate pipeline
- Ref 7 is for antigen retrieval with temperature AND pressure, but the current manuscript only uses temperature. Please use a different reference.

Reviewer #4

(Remarks to the Author)
I co-reviewed this manuscript with one of the reviewers who provided the listed reports. This is part of the Nature Communications initiative to facilitate training in peer review and to provide appropriate recognition for Early Career Researchers who co-review manuscripts.

Version 1:

Reviewer comments:

Reviewer #1

(Remarks to the Author)
The authors have addressed the comments in a generally comprehensive manner, and the revised manuscript better demonstrates the strength of the method compared with existing approaches, particularly through the addition of the 120 SPD comparison. However, the reviewer still has the following concerns:
1.For major comment No. 3, regarding Figure S4C, the reviewer has concerns about the comparison between the hanging droplet method and scDVP. The standard deviation for scDVP is quite large, indicating substantial variability between replicates, and the authors should clarify the source of this variability. Additionally, for Protein Groups (PG), the hanging droplet method appears to identify fewer proteins than scDVP in this panel, which seems inconsistent with the claim that the method performs better. Finally, the figure legend and main text do not clearly specify the data sources used for this comparison, including tissue type, sample area, gradient length, and number of replicates. The authors should provide these details to ensure that the comparison is transparent and reproducible.
2.In line 855, the concentration should be 25 mM, not 25 nM.
3.The Methods section remains too brief. Important details, including the use of TEAB and the concentration of DDM, are not clearly documented, despite being discussed in the Results and figure legends. A comprehensive Methods section is necessary for others to reproduce the workflow.
4.Regarding the DDM comparison, the reviewer considers the current comparison inappropriate. The authors used 0.05% DDM, which is likely too high and may directly account for the observed decrease in performance. The previous study (PMID: 33649493) clearly reported that optimal SRM signals for most EGFR pathway peptides were achieved with 0.01–0.02% DDM, whereas higher DDM concentrations can saturate the LC column and substantially degrade chromatographic performance. Low DDM concentrations, such as ~0.015%, are also commonly used and have been shown to perform well in related workflows. Therefore, the authors need to repeat the comparison using an optimized low DDM concentration, such as 0.015%. Without this control, the current comparison is biased and does not support the conclusion that the hanging droplet method outperforms DDM.

Reviewer #2

(Remarks to the Author)
In the revised manuscript, the author provided additional supporting data (blanks), analysis, and discussions. They also lower their tone about the novelty of the work, calling it "improved throughput, scalability, and accessibility". They also cited additional references and clarified that most of the technical components have already been reported by other groups, such as the use of the proteoCHIP EVO 96 and the hanging drop approaches.

It is clear that the primary novelty of this work is the combination of the Evo-96 device with the hanging-drop approach, thereby enabling processing of multiple Evo-96 plates (192 samples across 2 plates).

However, the overall response and presentation are disappointing. If the goal of the technology development is to improve the throughput, robustness, and scalability, can you demonstrate this feasibility in the manuscript? Is it not convincing to see the major claim of throughput and robustness, but it only shows the total number of samples below 20. There must be many other technical hurdles to overcome to practically analyze > 192 samples.

As a technology development manuscript, it is essential to compare existing technologies. Both Review 1 and 2 (myself) ask

for the benchmarking study. It is important to assess the performance of this technology against the established openDVP (EVO96) or DVP workflow (384-plate). The authors ignore these requirements and only use published data for comparison, which is known to be unfair, as different MS (TIMS-TOF vs ASTRAL) are used. It should be noted that the Olsen group has all the required resources, as indicated by their recent publications.

The blank data is highly critical and should be included in the main figure (Figure 4F). The author should note how many proteins from the single nucleus are also detected in blank data (reagent and LMD membrane).

Supplementary Fig. 8C, please check the Y-axis label. It doesn't make sense to have 80k to 160k peptides.

Reviewer #3

(Remarks to the Author)
The authors have substantially improved the manuscript, and I especially appreciate the addition of process and reagent blank controls for the single-nucleus measurements, which directly address my concern that the reported identifications could partly reflect background. The clarification of the on-tip comparison is also welcome, thank you!

I have now tried the method myself but could unfortunately not entirely reproduce the authors' findings. In particular, I found dramatically varying buffer volumes in the Evochip wells after the hanging-droplet incubation. A few parameters of this step were unclear to me, and for the method to be useful I would ask the authors to expand the description of the decrosslinking incubation step, which is currently described in only about three lines (ll. 547–549).

Specifically: which 96-well plate was used (manufacturer and type), and whether a lid was used during the 95 °C incubation, and if so at which temperature.

In addition, I would like to re-iterate some requests that I think should be addressed in writing:

- Beyond the incubation step above, my original concern about evaporation over a realistic collection run was not addressed. A one-hour time course was measured and collection was stated to take 20–30 min, but full-plate runs can in practice be hours to days, and the data does not cover that case. Please add this as a limitation to the discussion.

- The response describes the 0.13 µL/min rate as measured at decrosslinking conditions, whereas the manuscript text states standard laboratory conditions. Please clarify, since collection occurs at room temperature with the droplet exposed. And the manuscript states that DDM compromises droplet stability (Supplementary Fig. 1F shows reduced remaining volume with DDM), whereas the response to my comment states that DDM reduces evaporation. Please make these consistent.

- The clarification that biological replicates correspond to independent tissue regions means that the subtype comparisons rest on a single block per subtype. The authors note the single-patient design (ll. 443–446) but still frame the cross-subtype differences as biologically meaningful. Please add a clear statement that, because each subtype comes from a single sample, the differences shown between subtypes cannot be generalised and may reflect patient-to-patient variation rather than true subtype differences.

Reviewer #4

(Remarks to the Author)
I co-reviewed this manuscript with one of the reviewers who provided the listed reports. This is part of the Nature Communications initiative to facilitate training in peer review and to provide appropriate recognition for Early Career Researchers who co-review manuscripts.

Version 2:

Reviewer comments:

Reviewer #1

(Remarks to the Author)
The authors have done a great job in improving the reproducibility and accessibility of the method, particularly by providing the detailed Supplementary Note 1 and the accompanying video tutorial. These additions significantly enhance the usability of the workflow for the community.

One suggestion is that the authors may consider providing the tutorial videos as supplementary video files associated with the manuscript, rather than relying solely on external platforms such as YouTube, to ensure more stable accessibility and long-term availability.

Reviewer #2

(Remarks to the Author)
In this revised manuscript, the authors have addressed my primary concerns about scalability. I request that the authors

include the plots (two 96-plate datasets) in the supporting information; it is direct evidence for the claim of scalability.

Although the overall novelty is not excellent, the capability to analyze large-scale samples is quite valuable to the field.

I also appreciate the authors for including a reagent blank and prep blank in the main text to show the background of the technology.

Overall, I would recommend the publication if the above requirements are met.

Reviewer #3

(Remarks to the Author)
The authors have now provided a detailed account of the methods, and I find particularly supplementary note 1 including the Youtube video very helpful. Minor requests:

- Please check Fig. 7 of the supplementary note 1, which to me does not show a "representative outcome".
- I am confused about the optima reported and how they are stated in the Methods and Note 1, specifically decrosslinking temperature (90°, 95 °C?), timing (1.7h [please give in minutes instead], 2.5h, 2h?), and enzyme amount (5ng or 10ng/uL).
- The steps in Note 1 are off, e.g., Stage 5, steps 16-19, the section runs until step 24 (also Stage 4). Given note 1 will be extremely helpful, please revise carefully.
- “Cosias Fabian laboratory” should be “Fabian Coscia”.
- The Methods section itself has not been changed extensively, but a referral to Note 1 has been added. I would welcome some key details (Armadillo plate with pre-chilling at -20 °C, removal of the heating block lid, etc.) in the methods as this is the key innovation of the paper.
- In Fig. 1B, 25mM TEAB are optimal (also stated in the main text), but then Note 1 states 10mM TEAB. Could you please harmonize?

Besides these comments, I accept the replies by the authors.

Reviewer #4

(Remarks to the Author)
I co-reviewed this manuscript with one of the reviewers who provided the listed reports. This is part of the Nature Communications initiative to facilitate training in peer review and to provide appropriate recognition for Early Career Researchers who co-review manuscripts.

Reviewer #5

(Remarks to the Author)
I co-reviewed this manuscript with one of the reviewers who provided the listed reports. This is part of the Nature Communications initiative to facilitate training in peer review and to provide appropriate recognition for Early Career Researchers who co-review manuscripts.

Open Access This Peer Review File is licensed under a Creative Commons Attribution 4.0 International License, which permits use, sharing, adaptation, distribution and reproduction in any medium or format, as long as you give appropriate credit to the original author(s) and the source, provide a link to the Creative Commons license, and indicate if changes were made.
In cases where reviewers are anonymous, credit should be given to 'Anonymous Referee' and the source.
The images or other third party material in this Peer Review File are included in the article's Creative Commons license, unless indicated otherwise in a credit line to the material. If material is not included in the article's Creative Commons license and your intended use is not permitted by statutory regulation or exceeds the permitted use, you will need to obtain permission directly from the copyright holder.
To view a copy of this license, visit https://creativecommons.org/licenses/by/4.0/

POINT-BY-POINT REBUTTAL TO REVIEWER COMMENTS

High-Throughput Spatial Proteomics at Cellular and Subcellular Resolution via a Hanging Droplet Workflow

Authored by Hao C, Guzman U.H. & Olsen J.V.

General response by authors:

We sincerely thank the Reviewers for their thorough evaluation of our manuscript, *"High-Throughput Spatial Proteomics at Cellular and Subcellular Resolution via a Hanging Droplet Workflow*."; and for their constructive and insightful comments, which has significantly strengthened our study. We greatly appreciate the time and effort invested in reviewing our work, and we believe that their feedback has helped us to improve the clarity, rigor, and overall quality of the manuscript.

Below, we provide a detailed, point-by-point response to all comments in **blue text**. All revisions made in the manuscript are clearly indicated with *"quotation marks".*

Reviewer #1 (Remarks to the Author):

In this manuscript, the authors describe a hanging droplet-based, EVO 96-compatible sample preparation workflow for laser capture microdissection (LCM)-based spatial proteomics of minute FFPE tissue regions. The proposed method aims to streamline the processing of small tissue inputs, by integrating LCM, decrosslinking, digestion, and DIA-MS analysis.

The reviewer appreciates the authors' efforts to address technical challenges associated with spatial proteomics of FFPE samples and minute LCM regions. The study demonstrates technically competent execution and presents several application examples, including subcellular-scale analyses. However, the overall methodological advance appears modest. Similar LCM-based spatial proteomics workflows for FFPE tissues and small sample inputs have been reported previously, and the present work does not clearly establish a substantial advantage in terms of spatial resolution, proteome depth, or throughput, despite the use of state-of-the-art instrumentation.

If positioned as a methodology-focused study, the workflow would benefit from clearer articulation of novelty relative to recent literature, more rigorous benchmarking against established approaches, and substantially expanded methodological descriptions. These aspects are critical for evaluating the significance and reproducibility of the proposed method at the level expected for a Nature Communications publication.

We thank the reviewer for the constructive feedback and for recognizing the technical competence of our workflow. We would like to clarify that the primary advance of this study is not increased proteome depth or spatial resolution per se, but the democratization and acceleration of spatial proteomics through substantially improved throughput, scalability, and accessibility. Our workflow reduces sample preparation time by ~30% compared to faster workflows published to date (PMID: 41418981) and enables parallel processing of up to 192 LMD FFPE samples using a single standard thermoblock, without specialized automation, microfluidics, or custom devices. This throughput can be readily scaled by operating multiple thermoblocks in parallel, providing a simple and cost-effective route to large-scale studies. We consider this a distinct methodological advance, as many existing high-sensitivity workflows rely on specialized infrastructure and limited parallelization, restricting scalability and broader adoption. In contrast, our approach enables robust, scalable, and widely accessible high-throughput spatial proteomics of FFPE tissues. Importantly, our simple but high-sensitivity workflow enables analysis of single nuclei with unprecedented proteome coverage.

Major Comments

1. The opening statement of the Abstract does not convincingly define a current methodological gap. Multiple state-of-the-art spatial proteomics approaches have already addressed FFPE samples and minute regions, including subcellular-scale analyses (e.g., Deep Visual Proteomics, and other related methods; PMIDs: 37909047, 35590073, 27062885, 40240610, 39477916, etc.). Moreover, challenges such as evaporation during decrosslinking and low throughput are not universal limitations across existing workflows.

We thank the reviewer for this important comment. We agree that several state-of-the-art spatial proteomics approaches, including Deep Visual Proteomics (DVP) and related methods, have demonstrated the analysis of FFPE tissues and minute regions, including subcellular structures. However, the key methodological gap addressed here is not feasibility per se, but the lack of simple, scalable, and high-throughput workflows that can be implemented without specialized infrastructure. In particular, several existing methods (PMIDs: 37909047, 35590073, 27062885, 40240610, 39477916) rely on specialized robotics, SpeedVac drying steps, or extended incubation times for *de*crosslinking and digestion, limit throughput and accessibility. In contrast, our workflow reduces sample preparation time by ~30% compared to state-of-the-art workflow (PMID: 41418981) and enables parallel processing of 96 samples, scalable to 192 samples using standard laboratory equipment (thermoblock) without specialized automation. Importantly, our simple but high-sensitivity workflow enables analysis of single nuclei with unprecedented proteome coverage. This provides a straightforward, scalable, and accessible approach for large-scale spatial proteomics studies. Accordingly, the abstract now reads:

"Formalin-fixed paraffin-embedded (FFPE) samples are central for clinical research and spatial proteomics (SP), yet their analysis via liquid chromatography-mass spectrometry (LC-MS) remains challenging due to protein extraction losses, evaporation during decrosslinking, and inefficient sample preparation. Current methods often involve complex protocols or specialized equipment, limiting scalability, throughput, and proteome depth. We present a streamlined micro-FFPE proteomics protocol for laser-capture microdissection (LCM) that enables high-throughput processing in a 96-well format, completing the workflow from tissue lysis to LC-MS-ready tryptic peptides on Evotips in just 2-hours. By integrating whole-slide imaging, LCM, and narrow-window data-independent acquisition mass spectrometry (nDIA-MS), our approach optimizes lysis, decrosslinking, and digestion within nano-wells leveraging the hanging droplet concept. This design minimizes surface adsorption, enhances sample processing efficiency, reduces preparation time by ~30% compared with reference methods, and enables parallel handling of up to 192 samples with standard equipment. This enables the quantification of >5,500 protein groups (PGs) from 50,000 µm² regions (~250 cells), with robust performance down to 1000 µm² (~5 cells) with ~2,500 PGs identified. Application to breast cancer (BC) tissue resolves BC subtypes and microenvironments via biomarkers (TP53, HER2, PR). Even from ~60 µm² regions of single nuclei

identify ~1,800 protein groups, enabling microscale spatial proteomics approaching near-organelle resolution".

Notably, a recent study from the Coscia group (PMID: 41418981) reports a standardized LCM-based spatial proteomics workflow that integrates EVO 96 with further loss-reduced tissue processing using CellenONE, achieving high throughput and robust proteome depth from small FFPE regions. Given that the present study relies on a similar multiwell-based peptide preparation strategy, the novelty of the proposed workflow is not immediately apparent. In this context, the reported throughput of ~80 samples per day does not exceed the ~100 samples per day reported by the Coscia group using a comparable workflow with similar sample sizes and proteome depth. The authors should therefore clearly articulate what specific methodological advances are introduced beyond existing approaches.

We would like to clarify that the primary advance of our workflow lies in its simplicity, accessibility, and scalability without the need for specialized automation platforms. While PMID: 41418981 workflow achieves impressive throughput and proteome depth, it relies on integrated robotic systems such as EVO 96 and CellenONE for loss-reduced sample handling. In contrast, our workflow achieves comparable throughput and proteome coverage using only standard laboratory equipment, without robotics or specialized dispensing systems. Specifically, our approach reduces sample preparation time by ~30% compared to PMID: 41418981 and enables parallel processing of 96 samples, readily scalable to 192 samples using a single thermoblock, representing a 2x higher parallelization capacity. Importantly, throughput can be further scaled in a linear manner by operating multiple thermoblocks in parallel, without the need for specialized automation. This removes the dependence on dedicated robotic infrastructure and provides a cost-effective and broadly accessible route to high-throughput spatial proteomics.

Regarding throughput, we demonstrate the practical scalability of the workflow by achieving a LC-MS analysis throughput of 120 samples-per-day (SPD) in an independent experiment using 120 SPD LC gradients. This experiment confirms the feasibility of analyzing >100 processed samples per day while preserving biological signatures comparable to those obtained with the 80 SPD method, despite a slight reduction in proteome coverage with the 120 SPD gradient. Lines 234-250 now read:

"Once we established the robustness of the sample collection workflow, we evaluated our FFPE sample preparation approach using rapid Whisper Zoom liquid chromatography (LC) gradients on the Evosep One system (80 and 120 SPD), coupled via an electrospray source to an Orbitrap Astral mass spectrometer. This configuration employed a narrow-window data-independent acquisition (nDIA) method20,21, which has demonstrated the sensitivity required for analysis of 10-cell–level sample amounts (4000 µm², 5 µm). As expected, the 80 SPD method identified ~15% more PGs and ~25% peptides than the 120SPD method (Fig.2A). Nevertheless, both methods shared ~80% of the identified PGs (Fig.2B), enabling coverage of similar top ten Gene Ontology

(GO) biological process terms (Supplementary Fig. 4A). This indicates that, although the 120 SPD method moderately reduces overall proteome coverage, it retains the ability to capture comparable biological signatures to the 80 SPD method (Supplementary Fig. 4A). Closer inspection of the log_{10}-transformed protein intensity distributions showed that the 80 SPD method enabled identification of lower-intensity protein groups (PGs) and provided higher quantitative performance with a higher number of data points per peak (6 DPPP for 80 SPD versus 4 DPPP for 120 SPD) (Fig. 2C,D). Despite this difference, the 120 SPD method still identified key PGs relevant to BC biology, including PARP1, TOP2A, ERBB2, CDH1, and COL1A1, all of which fell within the three highest intensity quantiles of the log_{10}-transformed intensity distribution in both methods (Fig. 2E)".

2. The Introduction is insufficiently developed and does not comprehensively cover recent advances in spatial proteomics of FFPE and minute samples. Several key developments are missing or under-referenced. For example, Deep Visual Proteomics is discussed primarily via an early 2022 publication, while more recent work from the Mann group and other laboratories, some of which address closely related technical challenges, are not adequately considered. A more balanced and up-to-date overview is required to properly position the present work within the current literature.

We thank the reviewer for this important and constructive comment. We have revised the introduction to include these recent studies (PMIDs: 37909047, 35590073, 27062885, 40240610, 39477916) and to more clearly contextualize our contribution. Now lines 86-99 and 100-108 read:

86-99

"To mitigate these challenges, some approaches employ continuous replacement of buffer with in a heating system9,10, enabling robust proteome coverage and high throughput. However, these methods rely on specialized instrumentation9,10, which limits their scalability and broader applicability. Other workflows depend on multi-step processing, including SpeedVac drying11–13, repeated decrosslinking cycles11,12, and prolonged tryptic digestion11–13, all of which reduce throughput. More recently, innovative "hanging droplet" strategies have been developed to mitigate evaporation in a simpler and more practical manner. For instance, a "hang-down" droplet configuration has been implemented to enhance decrosslinking and peptide generation from microscale samples by minimizing evaporation during heating14. Similarly, the Surfactant-assisted One-Pot voxel method (wcSOP) employs a suspended droplet on the tube cap to process laser-capture microdissected material, improving sample retention and proteomic depth15. However, despite their effectiveness in preventing evaporation and enhancing sensitivity, these approaches remain inherently low-throughput, as they require processing samples individually".

101-109

"Our method leverages the proteoCHIP EVO 96 chip to directly collect samples from a laser-capture microdissection microscope system, employing a suspended droplet format to prevent evaporation during thermal decrosslinking while maintaining stable reaction volumes, and reduces sample preparation time by ~30% compared with reference methods10. This approach enables robust proteome profiling from extremely limited starting material, identifying ~5,500 protein groups (PGs) from FFPE sample regions containing ~ 250 cells and ~ 2500 PGs from regions containing ~5 cells using an 80 samples-per-day (SPD) LC-MS workflow. Comparable performance is retained under rapid-gradient conditions (120 SPD), covering ~90% of the proteome depth achieved at 80 SPD in 4,000 µm² BC sections".

3. To justify the use of a hanging droplet strategy for FFPE decrosslinking and digestion, the authors should perform more comprehensive benchmarking against established workflows from the other groups, like the Mann and Coscia groups. Currently, comparisons are made primarily to the One-Tip method, which was originally developed for cultured single-cell proteomics and is not an appropriate reference for FFPE minute-sample preparation. Without direct comparison to state-of-the-art FFPE LCM workflows, it is difficult to assess whether the proposed approach provides meaningful advantages.

We thank the reviewer for this important comment. The hanging droplet strategy is specifically designed to streamline FFPE decrosslinking and digestion immediately after sample collection, eliminating intermediate steps such as SpeedVac drying or extended incubation times. This approach both reduces evaporation and significantly shortens preparation time. For example, compared to the workflow described in PMID: 41418981, the hanging droplet reduces sample preparation time by ~30%, while compared to the method in PMID: 40240610, the reduction reaches ~90%. These improvements directly enable high-throughput processing of FFPE LCM samples without specialized robotics or additional equipment. We have introduced a new section in lines 251–262 positioning the performance of our method in the context of the latest published datasets, with the benchmark illustrated in Supplementary Fig. 4 b,c,d, which reads as follows:

"To place our method in context with current state-of-the-art standardized LMD-based spatial proteomics workflows, such as scDVP12 and openDVP 10, we benchmarked our workflow against published datasets. Although the sampled tissues differed, this comparison enabled evaluation of performance at minute sample inputs and under rapid LC gradient conditions.

Encouragingly, our method identified 27% more PGs and 38% more peptides than openDVP for samples with same area and thickness (8,000 µm², 5 µm) using the 80 SPD gradient (Supplementary Fig. 4B). Despite differences in tissue type, this comparison highlights the identification performance of our method at comparable input amount and gradient length. Furthermore, when compared with scDVP at similar single-cell equivalent inputs based on volume (2,000 µm³), our approach identified ~30% more peptides and comparable number of PGs (Supplementary Fig. 4C). The reason that scDVP detected PGs at lower intensity levels is likely attributable to the longer online LC gradient used in that approach (Supplementary Fig. 4D).".

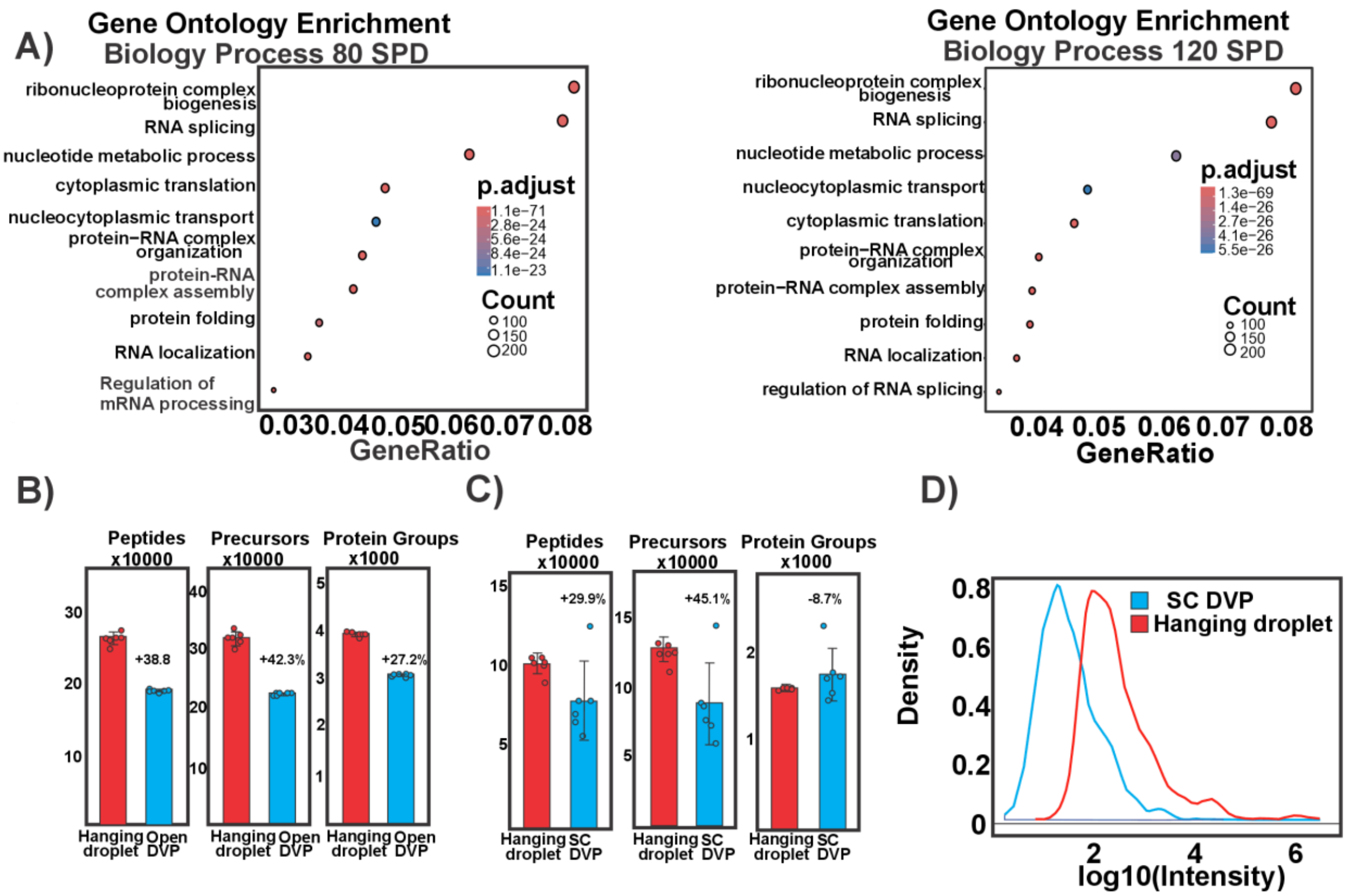

Supplementary Fig. 4. Benchmark of the hanging droplet method against existing spatial proteomics workflows. (A) Top ten Gene Ontology (GO) biological process (BP) terms enriched among proteins identified using the 80 SPD (right panel) and 120 SPD (left panel) methods. Terms are ranked by GeneRatio. Statistical significance was assessed using a hypergeometric test with Benjamini–Hochberg correction (adjusted $P < 0.05$). **(B)** Peptide, precursor, and protein group identifications from 8,000 µm² regions at 5 µm thickness analyzed by nDIA with the hanging droplet method, compared to OpenDVP DIA-PASEF reported by Nimo et al. (2026). **(C)** As in **(B)**, but for single-cell equivalent inputs, comparing the hanging droplet method to SC DVP reported by Rosenberger F. et al. (2025). **(D)** Log10-transformed intensity distribution of five single-cell equivalents processed using the hanging droplet method, compared to single-cell DVP data reported by Rosenberger F. et al. (2025). Data are presented as mean ± SD.

4. The authors state that n-dodecyl-β-D-maltoside (DDM) was mainly introduced to prevent protein adsorption to surfaces. However, this is not the primary rationale for DDM usage in the proteomics community. A previous study (PMID: 33649493), which is widely cited and adopted,

demonstrates that DDM can also improve enzymatic digestion efficiency and overall proteome recovery when applied under well-defined conditions. In contrast, the present study reports fewer protein identifications in the presence of DDM, which is opposite to the published observations. However, the manuscript does not provide additional analyses to clarify the underlying reasons for this discrepancy, such as assessments of missed cleavage rates, digestion efficiency, or changes in protein class composition (e.g., membrane-associated versus soluble proteins). Moreover, the Materials and Methods section lacks critical details regarding DDM usage, including its concentration and timing of addition. Given that DDM concentration is known to be a key determinant of its performance, the lack of methodological transparency makes it difficult to evaluate the results or assess reproducibility.

We thank the reviewer for raising this important point. As noted, the study (PMID: 33649493) reports the use of n-dodecyl-β-D-maltoside (DDM) and states that "DDM can also enhance trypsin and Lys-C enzyme activity," citing PMID: 18183947. However, as detailed in PMID: 18183947, this effect primarily arises from improved solubilization of membrane proteins, which enhances digestion efficiency and membrane proteome coverage. To address the reviewer's concerns, we performed additional experiments evaluating the addition of DDM at different stages of the sample preparation workflow, as shown in Fig. 1d,e and Supplementary Fig. B–D. Lines 157–177 now read:

"Next, we tested the effect of adding the MS-compatible surfactant n-Dodecyl β-D-maltoside (DDM) to LMD-excised micro-FFPE BC samples (20,000 µm², 10 µm thickness) prepared in ABC and TEAB buffer systems. Although DDM is commonly used to reduce protein adsorption to plastic surfaces, a major source of loss when handling minute specimens such as single cells and micro FFPE material, its omission in both buffer conditions resulted in higher numbers of identified PGs and modified peptide sequences. Among the tested buffers, TEAB consistently demonstrated better performance, yielding greater protein and peptide recovery in combination with improved reproducibility, as reflected by lower coefficients of variation (CVs) (Fig. 1D,E). The same trend was observed whether DDM was added before (B) or after (A) the decrosslinking step (Supplementary Fig. 1B,C). Furthermore, to test this effect in greater detail, we evaluated a DDM concentration range from 0 to 0.02 percent in the optimal 25 mM TEAB digestion buffer using BC samples (20000 µm² BC sections (5 µm thickness). Reassuringly, we observed a consistent decrease in identified PGs and peptides with increasing DDM concentration (Supplementary Fig. 1D). Given that DDM is also known to enhance solubilization of membrane-associated proteins, we compared the subcellular distribution of identified proteins across conditions (Supplementary Fig. 1E). Although a slightly higher proportion of membrane proteins and proteins associated with extracellular regions was detected when 0.05% DDM was added to the digestion buffer, the overall number of identifications remained consistently higher when using TEAB alone. This behavior likely reflects competing effects of the detergent: while DDM can reduce sample loss by limiting surface adsorption and improve stabilization of membrane proteins, it also alters droplet

surface tension, which may compromise droplet stability during heating (Supplementary Fig. 1F)".

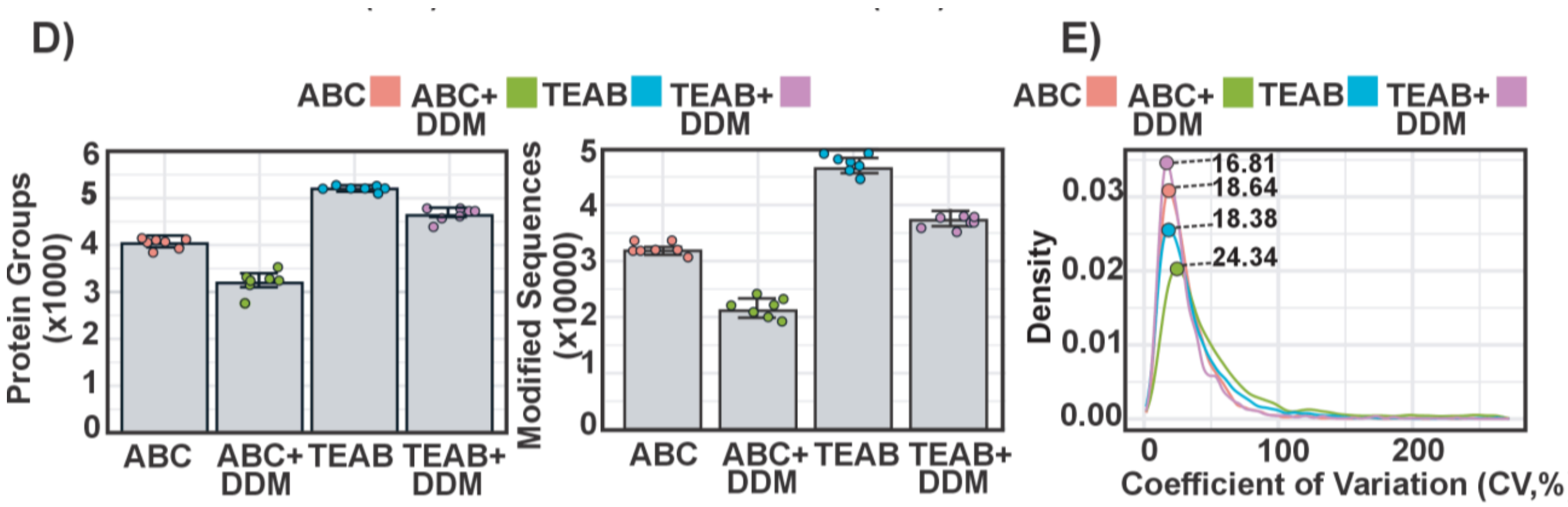

Fig.1. High-throughput hanging droplet workflow for micro-FFPE spatial proteomics. (D) Comparison of Protein Groups and Modified Sequences identified using Ammonium Bicarbonate (ABC) versus Triethylammonium Bicarbonate (TEAB) digestion buffers with or without DDM. (n=7, biological replicates) **(E)** Distribution of coefficients of variation (CV) across different buffer systems used during the digestion process. Colored points indicate the median CV for each condition.

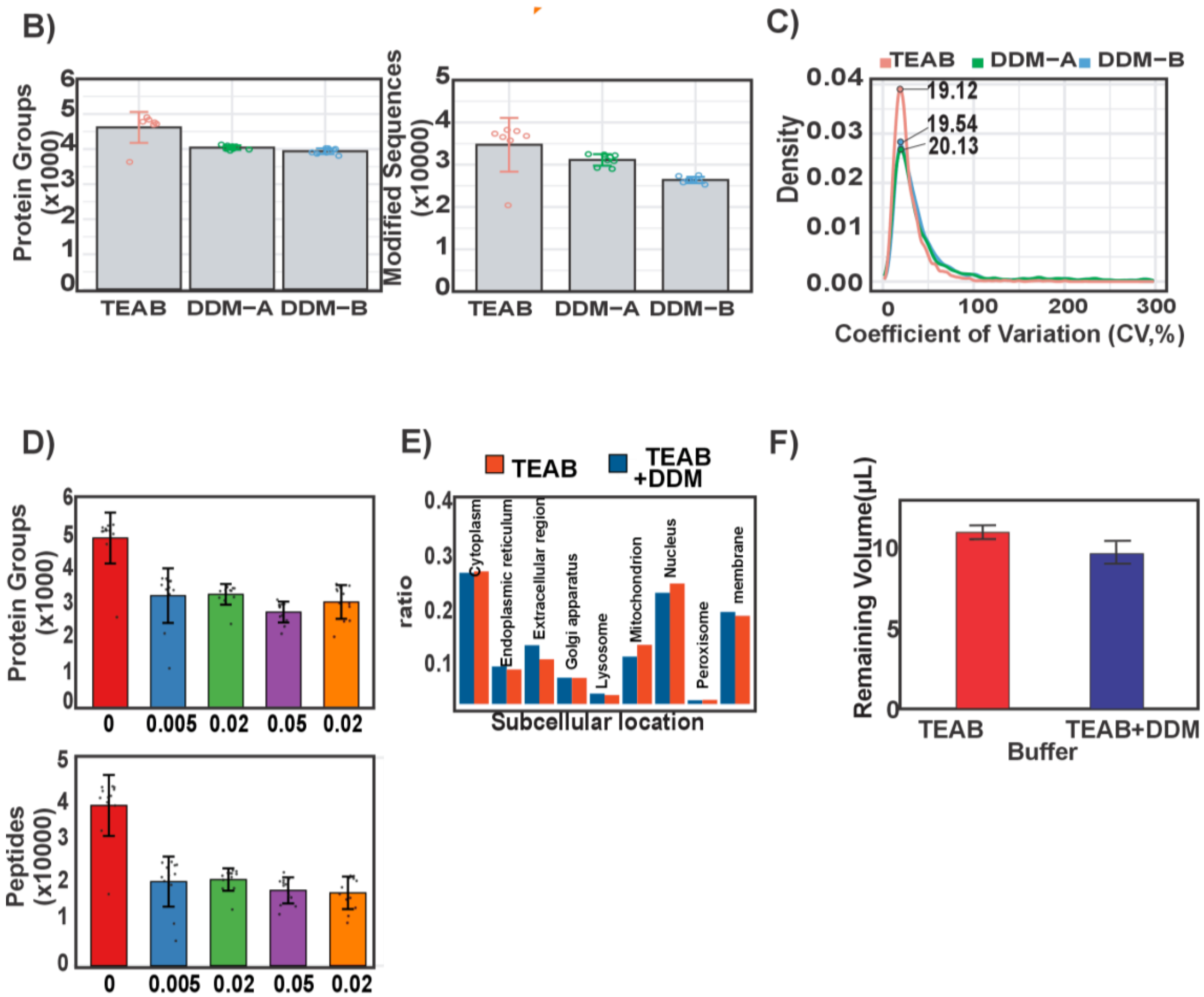

Supplementary Fig. 1. Conceptualization and optimization of the hanging droplet FFPE workflow. (B) Comparison of Ammonium Bicarbonate (ABC) and Triethylammonium Bicarbonate (TEAB) buffer systems adding DDM detergent before and after crosslinking process. **(C)** Distribution of Coefficients of Variation (CV) across different buffer systems adding DDM detergent before and after *de*crosslinking process. **(D)** Bar plots showing protein group (Top panel) and peptide identifications across varying DDM concentrations in 25 nM TEAB buffer during digestion (Bottom panel). **(E)** Proportion of subcellular localization categories among proteins identified under optimal ABC and TEAB conditions (25 nM). **(F)** Remaining reaction volume after *de*crosslinking (90 °C, 30 min) in 25 nM TEAB buffer with and without DDM (0.005%). Data are presented as mean ± SD.

5. The Materials and Methods section is overly brief and appears incomplete. Beyond limited descriptions of sample preparation, key details related to downstream statistical analysis and bioinformatics processing are missing or unclear. This lack of detail further limits the ability to critically evaluate the robustness and reproducibility of the reported results.

We thank the reviewer for this constructive comment. The Materials and Methods section has been substantially revised to provide additional detail on sample preparation and analysis, as well as downstream statistical analysis and bioinformatics processing, thereby improving clarity, transparency, and reproducibility. Briefly, in the revised manuscript we now include a more detailed description of the on-tip digestion workflow, expanded sample preparation information including a full list of reagents and step-by-step procedures, as well as a comprehensive account of all data processing steps. This includes criteria for missing value filtering, the imputation strategy (SLSA algorithm implemented in DAPAR with defined parameters), and normalization procedures (limma normalizeBetweenArrays using cyclic loess). We further clarify the statistical analysis workflow, including the use of moderated t-tests and ANOVA within the limma framework, as well as GO enrichment analysis using clusterProfiler with appropriate multiple testing correction. Version numbers for R (v4.4.2) and all relevant packages are also provided. We believe these additions significantly strengthen the Methods section and improve the reproducibility of the reported analyses.

6. Figure 1a is poorly presented and contains multiple issues, including misaligned graphical elements, apparent design errors (e.g., stray crossing lines), and inappropriate labeling (e.g., numbering within "Section V"). In addition, the inclusion of standard H&E staining as a major component of the workflow diagram is not acceptable, as it is neither novel nor a defining technical element of the proposed method. Overall, the figure is difficult to follow and requires substantial revision to clearly convey the workflow.

We thank the reviewer for this comment. Figure 1a has been fully revised to improve clarity, structure, and visual consistency. The graphical layout has been corrected by removing misaligned elements and unintended design artifacts, and the labeling has been revised, including correction of numbering within "Section V", to ensure a coherent and logically structured workflow representation. In addition, the figure has been reconfigured to more clearly emphasize the hanging droplet–based workflow, which represents the central methodological innovation of this study. We believe that the revised Figure 1A now more clearly and accurately reflects the experimental workflow and the methodological contributions of the study.

[editorial note: third party material redacted]

Fig.1. High-throughput hanging droplet workflow for micro-FFPE spatial proteomics. (A) Schematic overview of the integrated workflow combining whole-slide imaging, laser capture microdissection, and suspended droplet sample processing on EVO96 chips.

7. The Discussion session does not contain a balanced positioning of the method, and comparison with the state of the art methods in the literature.

We thank the reviewer for this important observation. In the revised manuscript, we have discussed our approach to recent high-throughput FFPE LCM workflows (37909047, 35590073, 27062885, 40240610, 39477916), including those from the Coscia and Mann groups, highlighting both the similarities and differences. We have now emphasized that, while existing methods achieve high proteome depth and throughput, they often rely on specialized robotics, extended processing times, or complex intermediate steps. In contrast, our workflow focuses on simplicity, scalability, and accessibility, reducing sample preparation time by ~30–90% depending on the reference method, enabling parallel processing of up to 192 samples with standard laboratory equipment, and facilitating large-scale spatial proteomics studies without specialized infrastructure. Accordingly, we have introduced a new section in the Results (lines 255–262) and Discussion (lines 440–448) to contrast our workflow with other existing methods, which reads as follows:

255-262

"Encouragingly, our method identified 27% more PGs and 38% more peptides than openDVP for samples with same area and thickness (8,000 µm², 5 µm) using the 80 SPD gradient (Supplementary Fig. 4B). Despite differences in tissue type, this comparison highlights the identification performance of our method at comparable input amount and gradient length. Furthermore, when compared with scDVP at similar single-cell equivalent inputs based on volume (2,000 µm³), our approach identified ~30% more peptides and comparable number of PGs (Supplementary Fig. 4C).The reason that scDVP detected PGs at lower intensity levels is likely attributable to the longer online LC gradient used in that approach (Supplementary Fig. 4D)".

451-459

"Spatial proteomics aims to characterize protein expression within the structural context of tissues, enabling insights into cell–cell communication, microenvironmental influences, and functional specialization of cell states that are not captured by bulk or dissociated single-cell proteomics[57]. However, the widespread adoption of spatial proteomics has been constrained by practical challenges, particularly the efficient extraction and digestion of proteins from microscale tissue regions, as well as the use of specialized equipment or low-throughput methods to tackle these challenges. To address this, we introduce a scalable high-throughput workflow based on the hanging droplet concept that enables reproducible processing of up to 96 laser-microdissected samples in parallel, generating LC-MS–ready material in ~2 hours.".

Minor Comments

1. The title may be overly broad. Including both "cellular" and "subcellular" resolution may be unnecessary, as achieving subcellular resolution inherently implies cellular resolution. A more concise title may be preferable.

We thank the reviewer for this thoughtful suggestion. We have revised the title to improve conciseness and avoid redundancy in the description of spatial scales. The title now reads:

"High-Throughput Spatial Proteomics from Cellular to Subcellular Scales via a Hanging Droplet Workflow."

We believe this revised wording more clearly reflects the continuum of spatial resolution addressed in this study, while maintaining clarity on the workflow's applicability across spatial scales.

2. The manuscript formatting is inconsistent and appears to retain elements from a previous submission, including a "Science Advances" footer. Furthermore, inconsistencies in layout, spacing, and section formatting affect the readability and overall professionalism of the manuscript. All formatting issues and remnants from previous submissions should be carefully corrected.

The manuscript has been carefully revised, and all formatting inconsistencies have been corrected.

3. The authors state that "2.5 µL of a 10 ng/µL trypsin and Lys-C mix" was used for digestion (25 ng enzyme). It is unclear for which sample input this amount was applied. For very small samples (e.g., a few cells or isolated nuclei), this enzyme amount appears excessive and should be justified.

We thank the reviewer for the comment. We have clarified the enzyme amounts used for digestion in relation to sample input size in the revised Materials and Methods section.

"Two experimental scales were defined: "single-cell level" (≤5 cells; ~2,000 µm², 5 µm-thick BC samples) and "micro-scale" (>5 cells). For single-cell level experiments, 2.5 µL of a 5 ng/µL trypsin/Lys-C mix was applied, corresponding to a total enzyme input of 12.5 ng. For micro-scale experiments, 2.5 µL of a 10 ng/µL enzyme solution was used".

4. The PRIDE raw data are provided as individual files spanning multiple acquisition dates, making it difficult to identify experimental batches and corresponding conditions. Grouping raw files from the same experiment into clearly annotated batches (e.g., via folders or a short README file) would substantially improve data organization and reuse.

We agree that the PRIDE repository was a bit confusing and have reorganized the PRIDE raw data files into clearly labeled batches corresponding to the experimental conditions. A README file and a metadata table linking each sample to its respective condition have also been added to improve clarity and ease of use.

Reviewer #2 (Remarks to the Author):

Chen H, H. Guzman, U et. al. described a sample preparation method for spatial proteomics of FFPE tissue. They integrated the approach with Evo96 chip, Evosep LC, and Astral MS with DIA analysis. They evaluated the approach's sensitivity and confirmed that it is consistent with existing technologies. They also applied to study proteomic differences between stromal and tumor regions, and the DE proteins were consistent with cancer biology. While it is a viable approach to improve the throughput of spatial proteomics, the reviewer is not convinced that such incremental improvement justifies publication in Nature Communications.

We thank the reviewer for the constructive feedback. We would like to clarify that the central advance of this study is not an increase in proteome depth or spatial resolution alone, but a step change in the practicality, scalability, and accessibility of spatial proteomics. Our workflow reduces sample preparation time by ~30% compared to the fastest currently published workflows (PMID: 41418981) and enables the parallel processing of 96 samples, readily scalable to 192 LMD FFPE samples using a single standard thermoblock, without robotics, microfluidics, or custom hardware. This throughput can be further increased simply by running multiple thermoblocks in parallel, providing a straightforward and cost-effective path toward large-scale studies.

We consider this a meaningful methodological advance, as many high-sensitivity spatial proteomics workflows rely on specialized infrastructure and limited parallelization, which restrict scalability and broader adoption. In contrast, our approach provides a robust, scalable, and broadly accessible route to high-throughput spatial proteomics while maintaining comparable proteome depth and sensitivity to existing methods. Importantly, our simple but high-sensitivity workflow enables analysis of single nuclei with unprecedented proteome coverage.

Main comments:

(1) The improvement of the hanging drop approach is mainly to facilitate high-temperature de-crosslinking in a small volume. The data shown in Figure 1 did indicates the improvement. However, they are using non-HIER-processed FFPE tissue. In most DVP or spatial proteomics, the tissues are pre-processed with HIER (high temperature epitope retrieval). Many groups (including my own lab) understand that the on-slide HIER is sufficient to de-crosslink proteins for spatial proteomics. Thus, the presented approach seems redundant, unless there is a case where tissue can not be HIER-processed. Note HIER is easy to perform and widely adopted in pathology labs.

The use of EVO96 chip for spatial proteomics is not new. Many papers have been published in the last several years. The work actually used a lot of protocol (Python script and 3D printed holders) from these published work.

We sincerely thank the reviewer for their insightful comments, which helped clarify the advantages and rationale of the hanging drop approach in this context. However, we want to emphasize that HIER is fundamentally a slide-level histological treatment performed prior to microdissection, whereas our approach addresses a different stage of the workflow: efficient de-crosslinking and digestion of microdissected material in microliter volumes after laser capture microdissection (LCM). As noted, HIER is a standard pre-processing step in pathology laboratories and some spatial proteomics studies. However, conventional HIER, as described in PMID:41418981 and PMID: 35590073, typically requires ~50 minutes and is applied to the entire sample, which can cause morphological distortion, tissue detachment, and reduced reproducibility across samples. In addition, further decrosslinking and processing steps are typically required after collection, increasing hands-on time and limiting overall throughput (PMID:41418981 and PMID: 35590073). In contrast, our hanging drop approach enables de-crosslinking and digestion immediately after sample collection, minimizing post-LCD handling. This strategy streamlines FFPE decrosslinking and digestion by removing intermediate steps such as SpeedVac drying or prolonged incubations, as compared to PMID:40240610. Additionally, the hanging drop method supports two-fold scalability and reduces sample preparation time by ~30% relative to the fastest approach published to date (PMID:41418981).

Regarding the use of the Evo96 platform and supporting tools (e.g., scripts and holders), we acknowledge that these components have been described previously and do not constitute the primary novelty of this work. Our contribution is not the hardware itself, but the integration of the hanging droplet chemistry with this infrastructure to enable a substantially simplified, faster, and more scalable FFPE spatial proteomics workflow. This integration allows parallel processing of 96 samples, easily scalable to 192 samples using standard laboratory equipment, without additional robotics or specialized automation.

(2) The authors compared the new approach with the "on-tip" approach, developed and used in the authors' lab. Note on-tip approach is not a common way to do spatial proteomics due to the technical challenge of collecting the tissue from Leica LMD to the tip. I am wondering if the author can compare their approach with the most widely used DVP approach, and use the HIER process tissue?

We thank the reviewer for raising this important point. We agree that on-tip digestion is not a standard approach in spatial proteomics due to the technical challenges highlighted. However,

our intention in performing this experiment was to evaluate the efficiency of in-droplet digestion compared to on-tip digestion following decrosslinking on the EVO96 chip.

Specifically, this experiment allowed us to benchmark the digestion efficiency achieved using the hanging-droplet approach on the chip against a previously validated low-volume processing strategy on tips. This comparison was feasible because the samples are already positioned on the EVO96 chip and can be readily transferred to pipette tips without additional processing complexity. In addition, we aimed to determine whether digestion performance would benefit from moving off-chip onto tips; however, we found that on-chip digestion provided superior performance and was therefore the preferred workflow (Lines 196-204).

"Finally, we compared on-chip hanging droplet digestion using the EVO 96 proteoCHIP with on-tip digestion performed directly on the C18 resin membrane inside an EvoTip, following transfer of the droplet from the proteoCHIP by centrifugation. Importantly, both conditions were carried out using the same input material and identical samples to ensure a fair comparison (FFPE BC samples 20,000 µm², 10 µm thickness). Encouragingly, we found that the hanging droplet approach enables the identification of twice as many protein groups and more than twice as many modified peptide sequences compared to on-tip digestion, while also showing a lower proportion of missed-cleaved tryptic peptides compared to the fastest published workflow to date using comparable digestion conditions[10] (Supplementary Fig. 2E,F)".

Importantly, our hanging-drop approach is fully compatible with upstream HIER treatment when immunohistochemistry is required, as well as with DVP-style collection if desired. The method is not intended to replace HIER or DVP, but rather to improve the efficiency of the critical post-LCM processing step, which remains necessary regardless of the upstream workflow.

(3) One of the biggest concerns is the lack of blank control samples when the authors present the proteome coverages of different tissue sizes. Because the laser dissection takes a long time and many air debris (protein contaminations) can get into the well. It is not clear how much contribution of the protein IDs is actually from dissected cells in addition to background contaminations. As such, the reviewer asks the author to include blank measurements from multiple empty sample prep wells on the same Evo96 chip

We thank the reviewer for this important and insightful comment. We agree that assessing potential background contributions is essential, particularly given the extended LCM processing times and the possibility of airborne or handling-derived contamination.

To address this concern, we have now included additional control experiments comprising both a process blank (i.e., an empty LCM membrane subjected to the full workflow, including sampling, collection, and all subsequent processing steps) and a reagent blank (i.e., processed without the sampling step). The results of these controls are now presented and discussed in Lines 336-353.

"To ensure that the detected signal originated from the collected material rather than from carry-over or background contamination introduced during collection or processing, we included two blank controls: a process blank (mimicking the whole workflow, including sampling and collection with an empty LMD membrane processed through all steps) and a reagent blank (with no sampling steps). Encouragingly, the signal intensity of the samples was more than three-fold higher than that of the blanks (Supplementary Fig.8 A, B). These controls were processed in the same batch as single-nuclei samples derived from tumor (Sn-T) and stroma (Sn-S) regions Consistent with this, we observed a marked increase in identification rates of protein groups (PGs) and peptides relative to blanks, with Sn-S yielding ~3.3-fold more PGs (~1,300) and ~20-fold more peptides (~8,000), and Sn-T yielding ~4.5-fold more PGs (~1,800) and ~35-fold more peptides (~14,000). (Supplementary Fig. 8 C, D). Moreover, PCA revealed clear separation between blanks and single-nuclei samples, with tumor and stroma nuclei also segregating according to their spatial origin in score space, despite sharing ~20% of identified PGs (Supplementary Fig. 8 E, F). To further investigate the co-identified protein groups (PGs) in blanks and samples, we performed Gene Ontology (GO) enrichment analysis, which revealed two main molecular signatures: regulation of proteolysis and peptidase activity, and keratinization (Supplementary Fig. 8E). These terms are consistent with trypsin-related proteases and skin-derived keratins (epithelial debris), which are common contaminants in proteomics workflows".

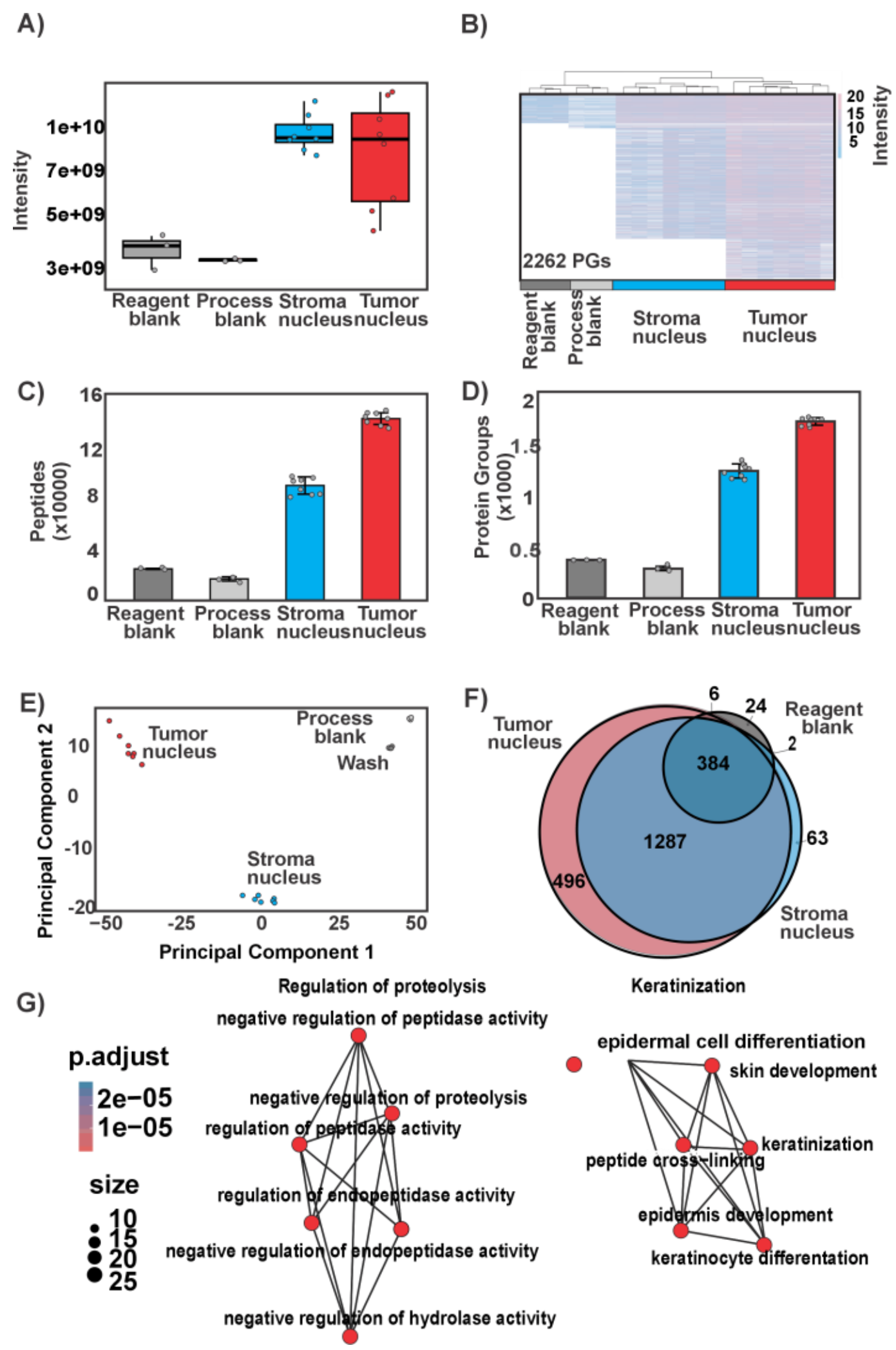

Supplementary Fig. 8. Benchmark of subcellular-resolution spatial proteomics in nuclear-enriched microregions. (A) Boxplot depicting the difference between Reagent blank (n = 3), Process blank (n = 3), Stroma_Nucleus (single nuclei from stroma, n = 8), and Tumor_nucleus (single nuclei from tumor, n = 8). **(B)** Heatmap showing protein expression intensity across different samples. **(C)** Bar plot showing peptide identification counts from nuclear-enriched stromal and tumor regions (~60 μm²), as well as reagent and process blanks. **(D)** Same as **(C)** but showing the protein group counts. **(E)** Principal component analysis illustrating differences between samples in score space. **(F)** Venn diagram showing the overlap of protein group identifications between blank samples and nuclear proteomes derived from distinct regions (stroma and tumor). **(G)** Gene Ontology (GO) enrichment analysis of biological process (BP) terms for proteins identified in blank samples. Statistical significance was determined using a hypergeometric test with Benjamini-Hochberg correction (adjusted p-value < 0.05). All box plots include the median line, the box denotes the interquartile range (IQR), whiskers denote the rest of the data distribution and outliers are denoted by points greater than ±1.5 × IQR. Data are presented as mean ± SD.

(4) Figure 2E, the author shows that the intensity distribution of different sizes is surprisingly similar, although the input changes > 50X folds. It is a confusing result. Could you plot the intensities of commonly detected proteins across these different inputs?

We thank the reviewer for drawing attention to the need for further clarification of the intensity distributions shown in Fig. 2E. We have therefore reanalyzed the data, focusing specifically on co-identified protein groups across all conditions, which are now presented in Fig. 2H. Reassuringly, we observe that the intensities of these co-identified protein groups scale with sample load and begin to saturate at 10,000 µm². Further discussion of this behavior is provided in Lines 271-278, which read:

"Nonetheless, low-input samples showed ~85% overlap in identified PGs with Pearson correlation coefficients of ~ 0.9 in their quantitative profiles, indicating high reproducibility and consistency even at minimal input levels (Supplementary Fig. 5 H,D). While larger samples exhibited higher overall protein intensity, signal intensity scaled with input as expected and reached saturation at loads exceeding ~50 cells (Fig. 5 H, I). Gene Ontology (GO) term enrichment analysis further showed that, despite fewer identifications in low-input regions, the core functional processes remained consistent (Supplementary Fig. 5E)".

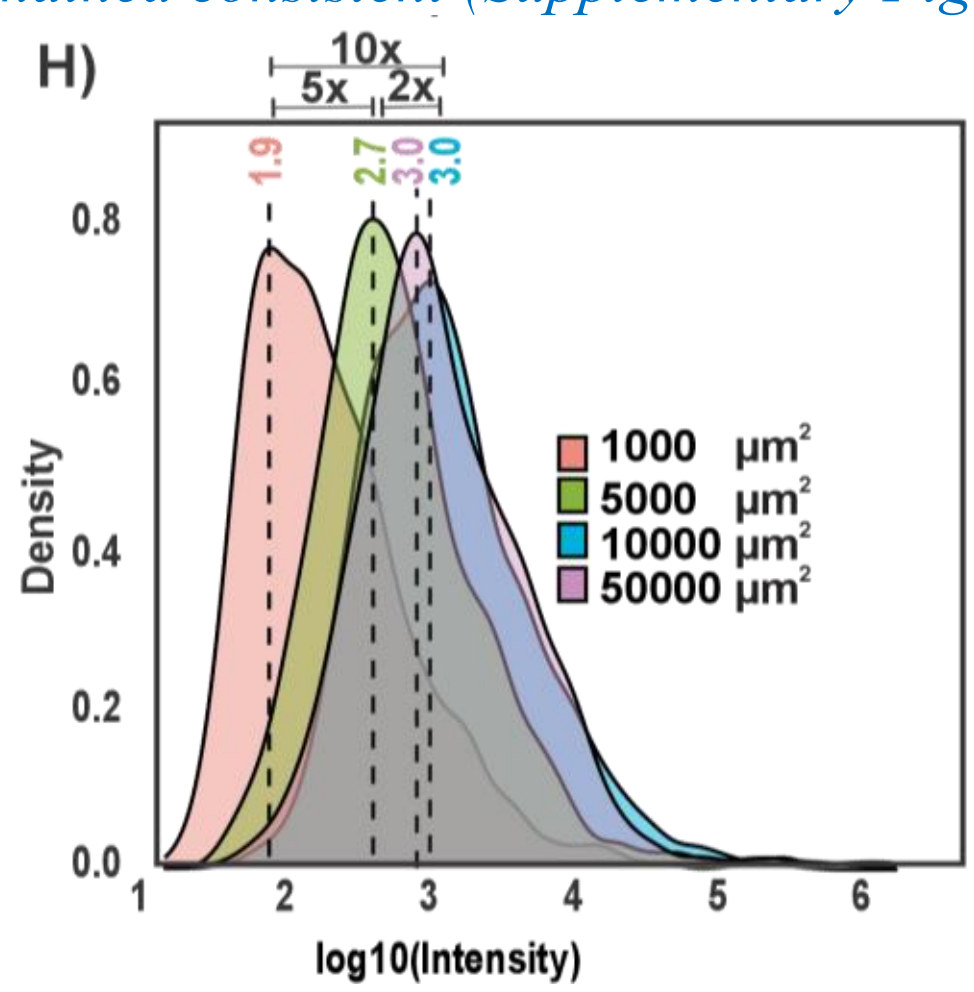

(H) Distribution of log10-transformed intensities of common protein groups across different microdissected region input sizes. Fold changes between conditions are indicated above.

(5) Figure 3A and B. The author indicates an average of 4500 proteins detected in the stromal and 5500 in the tumor. However, the Venn diagram only shows 2500-3200.

We thank the reviewer for pointing out this apparent inconsistency. It has been corrected in now Fig. 3B

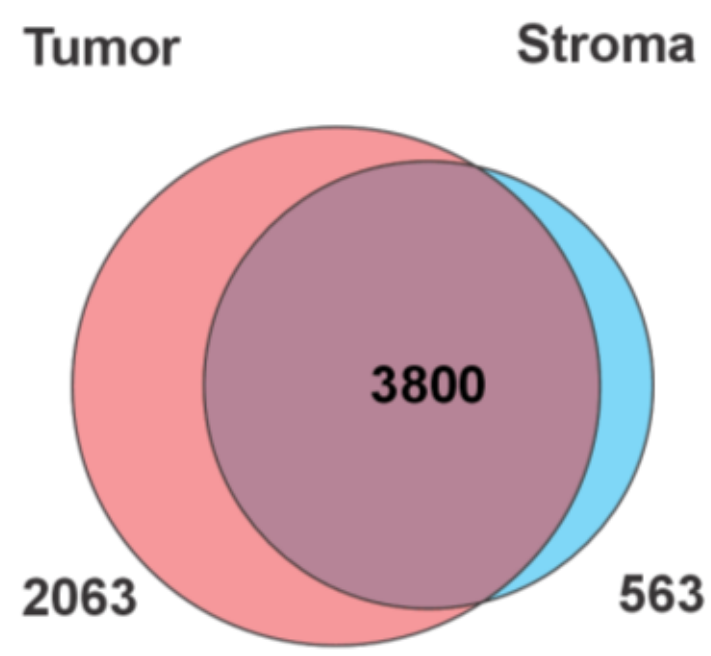

Venn diagram showing protein group identifications overlap between cancer and stromal microdissected regions.

(6) Figure 1B, the author shows that TEAB gives better sensitivity over the ABC buffer. There is no additional discussion on the buffer. Did the results agree with previously published data? It is noteworthy that ABC is among the most widely used buffers for label-free proteomics, whereas TEAB is primarily used for TMT proteomics.

We thank the reviewer for this comment. While ABC is more commonly used for label-free proteomics and TEAB is traditionally associated with labeling workflows, TEAB has recently been increasingly adopted for microscale proteomics, including single-cell and spatial applications (e.g., PMID: 38503780, 41418981, 41233595, 38513891, 40240610, 40934911). Discussion has been added in lines 139-156

"To establish a high-throughput and scalable spatial-proteomics workflow, we next defined optimal buffer conditions for Lys-C and trypsin proteolytic digestion within the hanging droplet by comparing two commonly used MS compatible digestion buffering systems: Ammonium Bicarbonate Buffer (ABC) and Triethylammonium Bicarbonate (TEAB), each tested across a range of concentrations from 0 to 100 mM applied to 20000 µm² BC sections (5 µm thickness). Notably, TEAB demonstrated better performance compared to ABC, yielding approximately 12.5% more PGs and 25% more peptide identifications at the optimal buffer concentration of 25 mM (Fig. 1B).

This improved performance of TEAB may reflect subtle differences in buffering behavior and physicochemical properties in microscale droplets. Although both salts provide comparable pH buffering capacity in solution, TEAB is less volatile than ABC and therefore may maintain more stable local ionic conditions during extended incubation in small-volume droplets.

The observation that the optimal buffer concentration for both systems is around 25 mM emphasizes the importance of balancing buffer concentration to maintain pH stability within the hanging droplet for effective tryptic digestion, while ensuring a low salt concentration to minimize disturbances in vapor pressure and surface tension within the system as such disturbances lead to reduced identifications or increased variability in the results. Regardless of the buffer system used at optimal buffer concentration, both systems identified similar subcellular terms, suggesting no apparent protein identification bias (Fig. 1C)".

Reviewer #3 (Remarks to the Author):

Chen, Guzman, and Olsen present a high-throughput spatial proteomics workflow using a hanging droplet configuration on EVO96 chips for processing FFPE samples. The method achieves reasonable proteome depth (about 5500 protein groups from 250 cells) with a streamlined 2-hour sample preparation time. The introduction is well written, and the language throughout is measured and avoids oversellling. That's refreshing, thank you! However, several technical clarifications and controls will be helpful:

We thank the reviewer for the positive and constructive assessment of our work and for recognizing the clarity of the writing and the streamlined nature of the workflow.

Major Comments

- The new method should add practicality to the field, however, a critical parameter missing from the manuscript is the time samples spend in the LMD instrument during collection. In our hands, a typical dissection protocol can take up to 18 hours for 96 wells. The authors should measure droplet volume over the duration of a typical collection run. This is essential to evaluate whether evaporation during collection undermines the advantages of the hanging droplet approach. In addition, the claim that DDM alters surface tension and increases evaporation (lines 151-153) would benefit from supporting data showing actual droplet volume reduction with DDM.

We thank the reviewer for this important and constructive comment regarding potential evaporation during prolonged LMD collection and the role of DDM in modulating droplet stability.

We agree that temporal stability of the hanging droplets is a critical parameter for practical implementation. We have now addressed this point in two ways:

New experimental data of droplet stability over extended collection times:

We have performed additional measurements tracking droplet volume over a 60-minute time course. These data show that droplet volume remains largely stable with an evaporation rate of 0.13 µL/min under decrosslinking conditions (90 °C) (Supplementary Fig. 3A). This corresponds to a loss that still allows a collection window of ~40 minutes while maintaining sufficient volume for the hanging droplet process. These results are discussed in the revised manuscript (lines 209–221) and demonstrate only minimal reduction under standard laboratory conditions. The new data have been included in the revised manuscript (Supplementary Fig. 3A):

"Because the objective of our method is to enable robust, high-throughput processing of more than 100 SPD from sample preparation through mass spectrometry analysis, we stress-tested the sample collection step of the workflow. This step represents a known limitation in laser

microdissection (LMD)–based workflows, where sample transfer from the LMD collection surface into the reaction chamber can compromise robustness and throughput18,19. We therefore optimized the liquid volume required during collection and evaluated the effect of adding buffer prior to collection versus collecting without pre-added buffer across different sample sizes. Under standard laboratory conditions, we measured an evaporation rate of 0.13 µL min^{-1} from an initial volume of 15 µL TEAB buffer on the chip pillar. This rate permits sample collection in a 96-well plate format for approximately 40 min while retaining sufficient buffer volume to support subsequent decrosslinking and proteolytic digestion using the hanging-droplet approach (Supplementary Fig. 3A). Although the collection rate depends on tissue area and the LMD cutting parameters, we found that 96 samples can typically be collected within 20–30 min under standard LMD settings.".

It is worth mentioning that the microdissection rate depends on the LMD settings, with a typical collection time of ~20 s per sample allowing 96 samples to be collected within approximately 30 min. In addition, if required, additional volume can be added to the Evo96 chip pillars after collection to ensure optimal conditions for the hanging droplet method, bringing the reaction volume to ~10 µL of buffer.

New experimental data to determine the effect of DDM on evaporation and surface tension:

To directly support the statement that DDM influences surface tension and evaporation behavior, we have added experimental measurements comparing the remaining droplet volume in the presence and absence of DDM (0.005) after subjecting the samples to decrosslinking conditions (90 °C). The results demonstrate a measurable reduction in evaporation rate when DDM is included (Supplementary Fig.1 F), consistent with its expected effect on surface tension modulation.

- The comparison to "on-tip" digestion (Fig. 1F-G) is unclear. Reading the methods (lines 448-464), both protocols appear to perform digestion in the EVO96 chip wells and the only apparent difference is using an incubator rather than the hanging configuration. This is not on-tip digestion as typically defined (e.g., the One-Tip method cited). The authors should either clarify the actual methodological difference or rename this comparison more accurately. Additionally, please confirm that the on-tip comparison used the same sample type and input amount.

We thank the reviewer for this important clarification request. In response, we have expanded the Methods section to provide a more detailed description of this experiment and have added an additional section to better clarify the rationale behind performing this comparison. Briefly, our intention was to evaluate the efficiency of in-droplet digestion on the EVO96 chip after decrosslinking, compared to an on-tip digestion workflow after decrosslinking and transfer

from the EVO96 chip to the Evotip. We further elaborate on this point in responses to other reviewers, where we discuss the methodological considerations in greater detail (Reviewer 2, point2).

- It is unclear whether replicates represent independent patients or multiple regions from single tissue blocks per subtype. If the latter, the statistical comparisons across breast cancer subtypes are not appropriately powered, and the claims need to be tempered accordingly. Please clarify the sample structure.

The sample structure has now been clarified to explicitly state that biological replicates correspond to independent tissue regions. We have also ensured that this distinction is consistently reflected throughout the manuscript. The statistical interpretation and associated claims have already been appropriately tempered to reflect this design.

- The single-nucleus proteome depths (1200 PGs) are impressive but require a blank or negative control (same workflow, just no material cut into the well during LMD). That will be essential to demonstrate that identifications are not background signal.

We agree that an appropriate blank control is essential to assess potential background contributions from the workflow. In response, we have included a section discussing the inclusion of process and reagent controls (lines 336-353). These blank controls were analyzed in parallel using identical reagents and instrument settings as for the samples.

Importantly, these blank runs yielded negligible peptide and protein group identifications compared to single-nucleus samples, supporting that the observed depth reflects true biological signal rather than background contamination.

We further elaborate on this point in responses to other reviewers (Reviewer 2, point 3).

- Please clarify whether match-between-runs (MBR) was used or files from different experimental setups were search together. I suggest to entirely separate experimental conditions during searching to avoid artificial inflation of identifications across conditions being compared.

The use of match-between-runs (MBR) has been clarified in the Methods section. In particular, files from different experimental setups were searched independently to avoid any artificial inflation of identifications. In addition, the method evaluation option was enabled for optimization experiments.

- Great to see the data on PRIDE, could you please reorganize the data for usability? That includes search results as .tsv/.csv files (not everyone has Spectronaut), group raw files by experiment in

zipped folders, and provide an overarching metadata file linking samples to experimental conditions. Also, the Data availability section states PXD071950 or so but the cover page has a different ID and credentials (that work for that ID). Please correct.

We thank the reviewer for the helpful suggestions regarding PRIDE data deposition. We have reorganized the old and new datasets into a consistent structure and added a README.csv describing the relationship between files and figures. All quantitative and identification tables have been uploaded as a single .zip archive, also containing a README for clarity, to ensure the data are fully accessible and usable.

Minor Comments

a) Results/Methods:

- Use LMD consistently throughout (LCM typically refers to thermoplastic film capture)

We thank the reviewer for this helpful suggestion. The requested changes have been implemented.

- Please comment on the 15% missed cleavage rate, is this expected given the workflow?

A 15% missed cleavage rate is a very reasonable value under the conditions used in the hanging droplet protocol, which involves a 1-hour digestion in a microreactor format. Moreover, comparison of the hanging droplet digestion efficiency with the fastest published method to date, using comparable digestion conditions (Supplementary Fig. 2F), shows a lower proportion of peptides with one or two missed cleavages in our approach (15.0% and 1.0%, respectively) compared to the fastest reported method (24.7% and 5.0%), further supporting reduced missed cleavages. Lines 197-201 now reads:

"Encouragingly, we found that the hanging droplet approach enables the identification of twice as many protein groups and more than twice as many modified peptide sequences compared to on-tip digestion, while also showing a lower proportion of missed-cleaved tryptic peptides compared to the fastest published workflow to date using comparable digestion conditions[10] (Supplementary Fig. 2E,F)".

- Supplementary Fig. 1A: specify optimal volume, show replicates with SD, clarify what the photo depicts

The requested changes have been implemented.

- Line 194: rephrase "was the primary source" to "was a major source", and consider "tissue architecture" instead of "spatial localization" **We thank the reviewer for this helpful suggestion. The requested changes have been implemented.**

- Fig. 2B: "greater variability" rather than "greater biological heterogeneity" (technical contribution unknown) **The requested changes have been implemented.**

- Describe tumor vs. stroma annotation procedure, was a pathologist involved?

Tissue region annotation was performed using established reference materials and by consulting an external pathologist to support the distinction between stromal and tumor regions. This information has been added to the Materials section.

- Report LMD sorting efficiency and dropout rates (we always have dropouts of 5-10%)

Dropout rates were calculated, demonstrating the benefit of improved collection efficiency when buffer is added for the collection of minute samples (Supplementary Fig. 3).

- Describe missing value handling and any batch correction in methods.

A description of the handling of missing values and normalization has been added to the Methods section. Independent experiments were performed within the same batch, thereby minimizing batch effects and avoiding the need for batch correction.

- Be consistent with PR/PGR nomenclature throughout

The nomenclature has been standardized throughout the manuscript.

- Missing reference at line 307 ("cancer cell invasiveness")

References added

- Add statistical analysis details for functional enrichment (e.g., Supplementary Fig. 5)

Details were added

- Fix duplicated H&E protocol text (lines 372-377)

Fixed

- Text uses "crosslinking" where "decrosslinking" is meant in several places

Discrepancies were corrected.

- Reduce redundancy in Results section, particularly for Fig. 1

Redundancy was reduced.

b) Figures:

- Unify font sizes and types across all figures; check for text/image overlap

- Fig. 1: fix "Ethonal" typo and clean up panel A composition

- Fig. 1G: add y-axis label

- Fig. 2E: add x and y axis labels

- Fig. 2F: add annotation within figure; clarify color gradient (z-score?)

- Fig. 5: dense and difficult to interpret; colors hard to distinguish

- Specify n in all figure legends

- Define error bars (SD) for all bar plots

- Add "z-score" label for color legends throughout

- Clarify protein labeling criteria in volcano plots, these seem random at the moment.

- Supplementary Fig. 1B: arrange before/after in left/right order

- Supplementary Fig. 1D: identify the red dot

- Supplementary Fig. 5: panel B not referenced in legend

- Supplementary Fig. 6: indicate which cluster from Fig. 5 these derive from

We thank the reviewer for these detailed and constructive suggestions. All figures have been re-rendered accordingly, and the requested revisions have been implemented throughout the manuscript. Specifically, font sizes and types have been unified, text and image overlaps corrected, and all panels adjusted for clarity. Typographical errors have been fixed (including "Ethanol"), and axis labels, annotations, and color scale definitions (including z-score labels where applicable) have been added. Figures with dense information (e.g., Fig. 5) have been redesigned for improved readability and clearer color discrimination. All figure legends now specify n values and define error bars (SD) for bar plots. Volcano plot labeling criteria have been clarified. Supplementary figures have been corrected as requested, including panel ordering, missing annotations, and legend references. In addition, Supplementary Fig. 6 now clearly indicates the corresponding clusters from Fig. 5.

c) References:

- Makhmut et al. (MCP 2024) cited twice (refs 9 and 47) **Corrected**

- Missing citation for openDVP/Nimo et al., which was clearly used for the QuPath-to-LMD coordinate pipeline **Added**

- Ref 7 is for antigen retrieval with temperature AND pressure, but the current manuscript only uses temperature. Please use a different reference. **Reference changed**

Reviewer #4 (Remarks to the Author):

I co-reviewed this manuscript with one of the reviewers who provided the listed reports. This is part of the Nature Communications initiative to facilitate training in peer review and to provide appropriate recognition for Early Career Researchers who co-review manuscripts.

Ok, thank you for the comments on our manuscript.

POINT-BY-POINT REBUTTAL TO REVIEWER COMMENTS (Second Revision)

High-Throughput Spatial Proteomics at Cellular and Subcellular Resolution via a Hanging Droplet Workflow

Authored by Hao C*, Guzman U.H.* & Olsen J.V.

We thank the Reviewers for their continued assessment of our manuscript in this second round of review. Their constructive comments have further enhanced the clarity and rigor of the revised manuscript.

Below, we provide a detailed, point-by-point response to all comments. All revisions made in the manuscript are clearly indicated in blue text.

Reviewer #1 (Remarks to the Author):

The authors have addressed the comments in a generally comprehensive manner, and the revised manuscript better demonstrates the strength of the method compared with existing approaches, particularly through the addition of the 120 SPD comparison. However, the reviewer still has the following concerns:

1.For major comment No. 3, regarding Figure S4C, the reviewer has concerns about the comparison between the hanging droplet method and scDVP. The standard deviation for scDVP is quite large, indicating substantial variability between replicates, and the authors should clarify the source of this variability.

We thank the reviewer for this comment. To improve the transparency and reproducibility of this comparison, we have revised the figure legend to clearly specify the experimental conditions used for the scDVP, Open DVP, and hanging droplet datasets, including the LC–MS configurations.

Figure legend now reads:

"Supplementary Fig. 4. Benchmark of the hanging droplet method against existing spatial proteomics workflows. (A) Top ten Gene Ontology (GO) biological process (BP) terms enriched among proteins identified using the 80 samples-per-day (SPD) (right panel) and 120 SPD (left panel) methods. Terms are ranked by GeneRatio. Statistical significance was assessed using a hypergeometric test with Benjamini–Hochberg correction (adjusted $P < 0.05$). (B) Peptide, precursor, and protein group identifications from 8,000 µm² triple-positive breast cancer sections (5 µm thickness) analyzed by nDIA using the hanging-droplet method (80 SPD, Evosep one; Orbitrap Astral MS), compared to Open DVP DIA-PASEF (Tonsil regions, 8,000 µm², 5 µm thickness, Evosep, 80 SPD; timsTOF Ultra AIP), as reported by Klingeberg et al. (2026). (C) As in (B), but for

single-cell–equivalent inputs, comparing the hanging-droplet method (80 SPD, Evosep one; Orbitrap Astral MS) to the scDVP workflow reported by Rosenberger et al. (2025) (40 SPD; FAIMS CV-40, Orbitrap Astral MS). (D) Log10-transformed intensity distributions of five single-cell–equivalent samples processed using the hanging-droplet method (triple-positive breast cancer regions, 5 µm), compared to single-cell DVP data (hepatocyte-like single shape, 10 µm thickness) reported by Rosenberger et al. (2025). Data are presented as mean ± SD."

Regarding the variability observed in the scDVP measurements shown in Supplementary Fig. 4c, most of the observed spread is attributable to a single outlier. Otherwise, the error bars reflect the variability across biological replicates in the published scDVP dataset. In this context, single-shape denotes the specific AI-segmented boundary of an individual cell within a tissue section that is isolated for targeted molecular analysis (variable size depending of estimated boundary). We have clarified these points in the revised figure legend. In addition, after removing the outlier, the variability in the scDVP dataset is substantially reduced, while the overall trends remain unchanged.

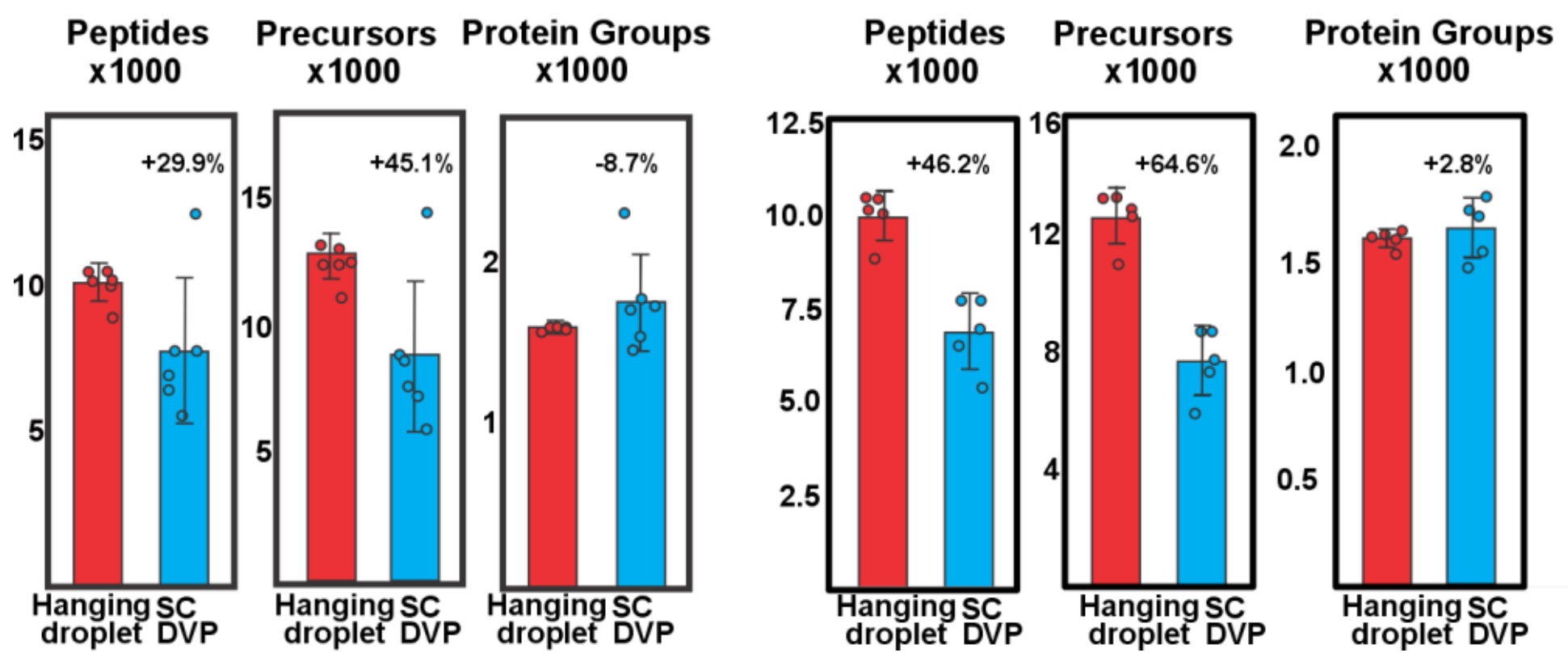

Comparison between the hanging-droplet approach (80 SPD) and scDVP (40 SPD): left panel, all scDVP data; right panel, scDVP data after removal of outliers.

Additionally, for Protein Groups (PG), the hanging droplet method appears to identify fewer proteins than scDVP in this panel, which seems inconsistent with the claim that the method performs better. Finally, the figure legend and main text do not clearly specify the data sources used for this comparison, including tissue type, sample area, gradient length, and number of replicates. The authors should provide these details to ensure that the comparison is transparent and reproducible.

We thank the reviewer for this valuable comment. The apparent discrepancy between the number of peptide identifications and protein groups arises primarily from differences in the LC–MS acquisition methods and configuration used between both datasets. Specifically, the published scDVP dataset from Rosenberger et al. (PMID: 40240610) was acquired using FAIMS

with a single compensation voltage (CV) of −40 with a 40 samples-per-day (SPD) LC gradient (Evosep; 15-cm Aurora Elite TS column), whereas the hanging-droplet dataset was acquired without FAIMS using an 80 SPD LC gradient (Evosep; 5-cm Aurora Elite TS column). These methodological differences affect peptide sampling and protein inference, which can lead to differences in the number of inferred protein groups and peptide identifications. In particular, the use of FAIMS with a single compensation voltage has been shown to increase proteome coverage at the expense of peptide coverage (PMID: 32051234). In addition, the hanging-droplet workflow identified a comparable number of protein groups to the scDVP workflow (approximately 3% fewer protein groups) while operating at twice the LC–MS throughput. We therefore consider this to represent a favorable trade-off between analytical throughput and proteome depth for low-input samples. To improve the readability of the manuscript, we have revised both the main text, the figure and the legend of Supplementary Figure 4 to explicitly state the tissue type, sample area, LC gradient length, acquisition settings, and the number of replicates used for each dataset. This additional information should make the comparison clearer for readers.

Now lines 255-256 reads : "Encouragingly, our method identified 27% more PGs and 38% more peptides than openDVP for samples with same area and thickness (8,000 µm², 5 µm) using the 80 SPD gradient (Supplementary Fig. 4B). Despite differences in tissue type, this comparison highlights the identification performance of our method at comparable input amount and gradient length. Furthermore, when compared with scDVP at similar single-cell equivalent inputs based on volume (2,000 µm³), our approach identified ~46% more peptides while achieving a comparable number of PGs (Supplementary Fig. 4C). Although scDVP identified slightly more low-intensity PGs, this is likely attributable to its longer online LC gradient (40 SPD) and the use of a single compensation voltage (CV) FAIMS acquisition strategy. Overall, the hanging-droplet methods represent a favorable trade-off between analytical throughput and proteome depth for low-input samples, providing substantial higher peptide identifications while maintaining comparable PG coverage in half analysis time (Supplementary Fig. 4D)."

2.In line 855, the concentration should be 25 mM, not 25 nM.

We thank the reviewer for pointing out this error. We have corrected the concentration from 25 nM to 25 mM in Line 855 of the revised manuscript.

3.The Methods section remains too brief. Important details, including the use of TEAB and the concentration of DDM, are not clearly documented, despite being discussed in the Results and figure legends. A comprehensive Methods section is necessary for others to reproduce the workflow.

We thank the reviewer for this helpful suggestion. In response, we have revised the Methods section to improve the clarity and reproducibility of the workflow. Specifically, we expanded the description of the experimental procedures and added the Supplementary Note 1 providing additional details to support reproducibility and facilitate implementation across laboratories.

4.Regarding the DDM comparison, the reviewer considers the current comparison inappropriate. The authors used 0.05% DDM, which is likely too high and may directly account for the observed decrease in performance. The previous study (PMID: 33649493) clearly reported that optimal SRM signals for most EGFR pathway peptides were achieved with 0.01–0.02% DDM, whereas higher DDM concentrations can saturate the LC column and substantially degrade chromatographic performance. Low DDM concentrations, such as ~0.015%, are also commonly used and have been shown to perform well in related workflows. Therefore, the authors need to repeat the comparison using an optimized low DDM concentration, such as 0.015%. Without this control, the current comparison is biased and does not support the conclusion that the hanging droplet method outperforms DDM.

We thank the reviewer for this important comment. Regarding the use of DDM, we have revised Supplementary Fig. 1d by adding x-axis labels to clearly indicate the experimental conditions. This should make it evident that the comparison includes a range of DDM concentrations spanning 0–0.2% (0, 0.005%, 0.02%, 0.05%, and 0.2% DDM). These data demonstrate that the reduction in proteome coverage is not limited to 0.02% DDM; rather, lower DDM concentrations, including 0.005% and 0.02%, also resulted in reduced proteome coverage compared with the condition without DDM. Thus, our conclusions are based on an evaluation across a broad concentration range and are not driven solely using 0.02% DDM.

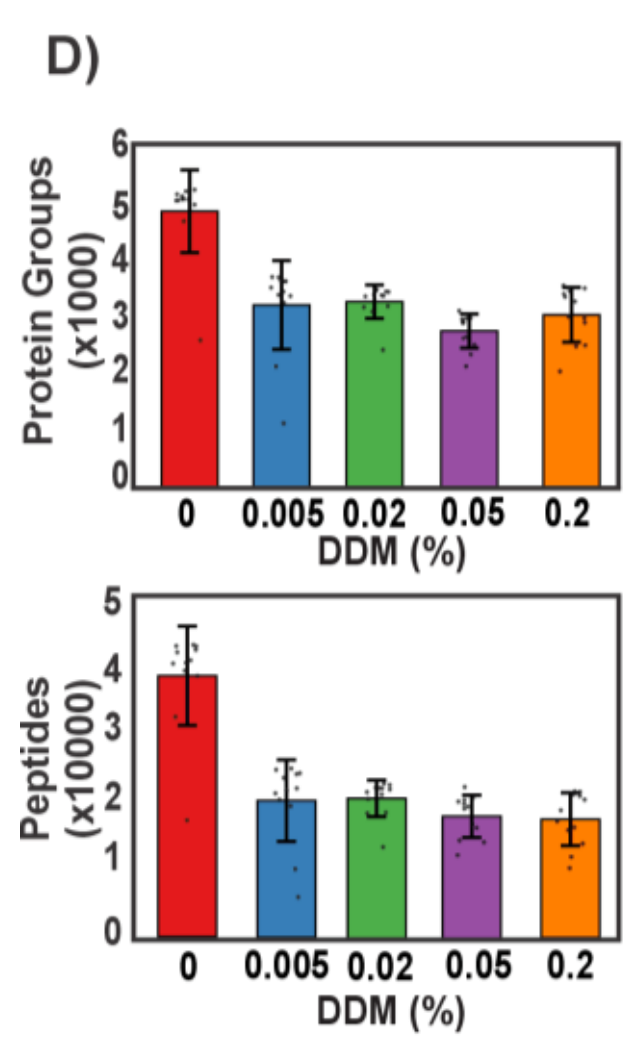

Supplementary Fig. 1. Conceptualization and optimization of the hanging droplet FFPE workflow. (D) Bar plots showing protein group (Top panel) and peptide identifications across varying DDM

concentrations (%) in 25 mM TEAB buffer during digestion (Bottom panel). Data are presented as mean ± SD.

Reviewer #2 (Remarks to the Author):

In the revised manuscript, the author provided additional supporting data (blanks), analysis, and discussions. They also lower their tone about the novelty of the work, calling it "improved throughput, scalability, and accessibility". They also cited additional references and clarified that most of the technical components have already been reported by other groups, such as the use of the proteoCHIP EVO 96 and the hanging drop approaches.

It is clear that the primary novelty of this work is the combination of the Evo-96 device with the hanging-drop approach, thereby enabling processing of multiple Evo-96 plates (192 samples across 2 plates).

However, the overall response and presentation are disappointing. If the goal of the technology development is to improve the throughput, robustness, and scalability, can you demonstrate this feasibility in the manuscript? Is it not convincing to see the major claim of throughput and robustness, but it only shows the total number of samples below 20. There must be many other technical hurdles to overcome to practically analyze > 192 samples.

We thank the reviewer for this important comment. We agree that demonstrating robustness and scalability is essential to support our claims of improved throughput. It is therefore important to emphasize that many of the optimization experiments underlying the development of this workflow were performed by processing complete 96-well Evo-96 plates in a single batch, ensuring experimental consistency while minimizing batch effects. In practice, we have successfully processed and analyzed up to 192 samples (two Evo-96 chips) demonstrating the scalability and robustness of the sample preparation platform. These samples were analyzed using an 80 samples-per-day (SPD) LC–MS method, and we further demonstrate the feasibility of increasing analytical throughput to 120 SPD, highlighting the compatibility of the workflow with high-throughput LC–MS acquisition strategies.

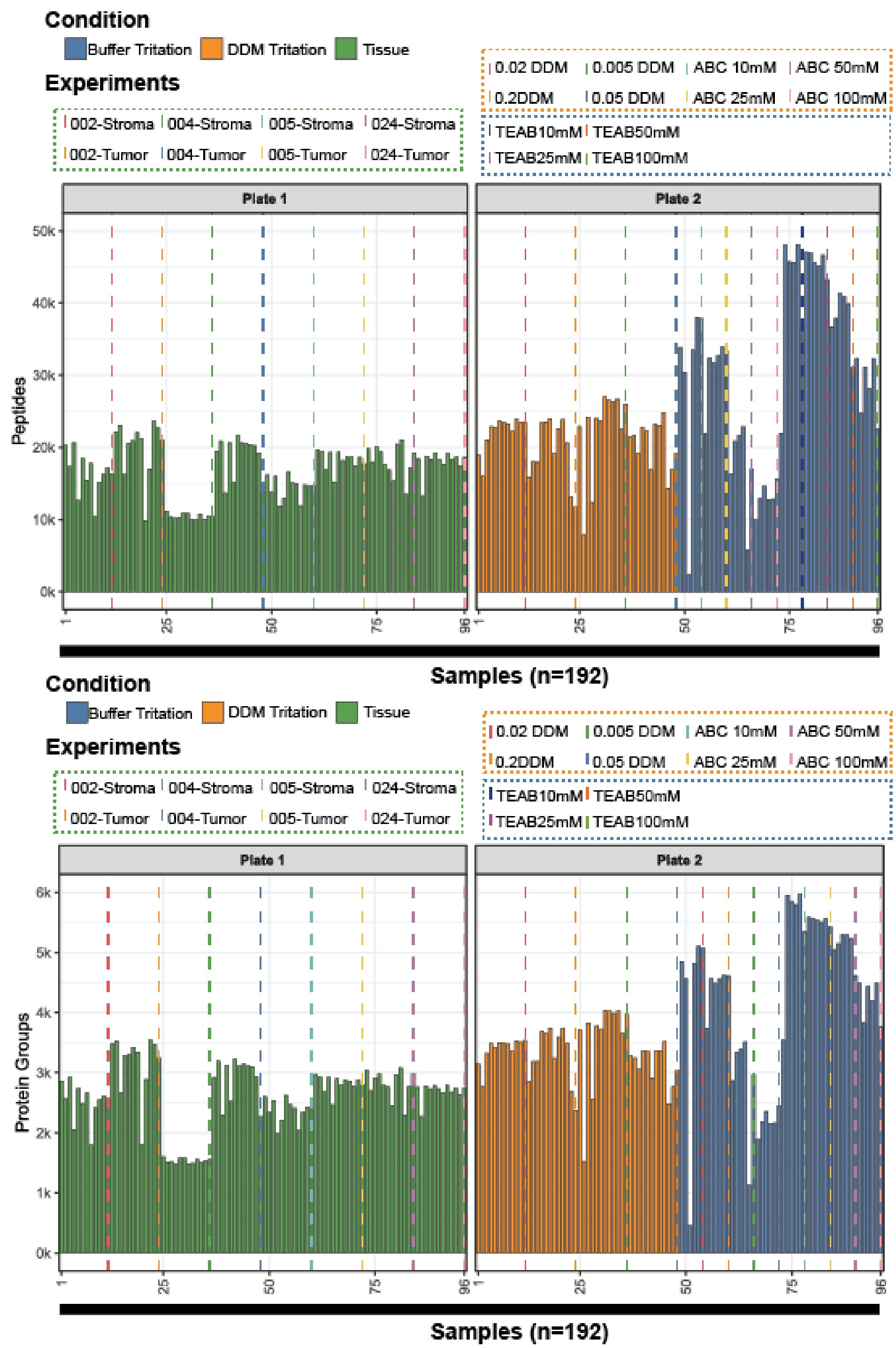

Method optimization experiments were processed in two full 96 well-plates and ProteoCHIP EVO 96, minimizing batch effects.

To better support these claims, we have revised the manuscript to explicitly state in materials and methods that the optimization experiments were performed using full 96-well plates and that the workflow has been successfully implemented for the preparation and analysis of 192 samples. We believe these additions provide stronger evidence for the robustness, scalability, and high-throughput potential of the proposed workflow.

" To ensure experimental consistency while minimizing batch effects, all optimization experiments were processed on Evo-96 plates in a single batch, enabling the successful processing and analysis of 192 samples in one workflow."

As a technology development manuscript, it is essential to compare existing technologies. Both Review 1 and 2 (myself) ask for the benchmarking study. It is important to assess the performance of this technology against the established openDVP (EVO96) or DVP workflow (384-plate). The authors ignore these requirements and only use published data for comparison, which is known to be unfair, as different MS (TIMS-TOF vs ASTRAL) are used. It should be noted that the Olsen group has all the required resources, as indicated by their recent publications.

We thank the reviewer for this constructive comment. We fully agree that benchmarking against established workflows is important for a technology development manuscript, and we appreciate the emphasis on a fair and rigorous comparison. At the same time, we acknowledge that a truly direct comparison between workflows such as openDVP (Evo-96) or the 384-well DVP format would require full implementation and optimization of each method under their respective optimal conditions. As the reviewer correctly notes, these workflows are highly developed within the originating laboratories, where substantial time, instrumentation, and optimization efforts have been invested to achieve maximal performance. Reproducing this level of optimization independently is both time- and resource-intensive and may still not fully recapitulate the performance achieved by the original developers.

For this reason, it is common practice in the field to use previously published datasets that represent the state of the art as performance benchmarks, provided that they are reprocessed using a consistent analysis workflow. This approach enables meaningful comparisons across technologies while minimizing potential biases arising from differences in optimization procedures and experimental implementation. We have followed this established benchmarking strategy in the present study.

The blank data is highly critical and should be included in the main figure (Figure 4F). The author should note how many proteins from the single nucleus are also detected in blank data (reagent and LMD membrane).

We thank the reviewer for this important suggestion and for highlighting the relevance of blank measurements for data interpretation. We fully agree that blank controls are essential for assessing background signal and potential carryover.

In the manuscript, we already present the overlap between proteins detected in single-nucleus samples and those identified in the reagent and LMD membrane blanks (Supplementary Fig. 8, now revised as Fig. 4). This analysis shows that only a minor fraction of proteins detected in single-nucleus samples are also present in the blank controls, indicating that most quantified proteins originate from true biological signal rather than background contamination. This is further supported by the substantially higher signal intensities observed in single-nucleus samples compared to blanks, which are on average more than three-fold higher (Supplementary Fig. 8A,B, now presented as Fig. 4 in the revised manuscript). In response to the reviewer's suggestion, we have further expanded this analysis by introducing a revised Figure 4 that includes the blank control data and provides a quantitative comparison between the blank signals and the single-nucleus dataset.

Supplementary Fig. 8C, please check the Y-axis label. It doesn't make sense to have 80k to 160k peptides.

We thank the reviewer for this observation. It was indeed a mistake that we labelled the y-axis peptide counts in numbers of 10,000x instead of the correct 1000x. We have corrected the Y-axis label in the revised Supplementary Fig. 8C to accurately reflect the reported metric and avoid confusion.

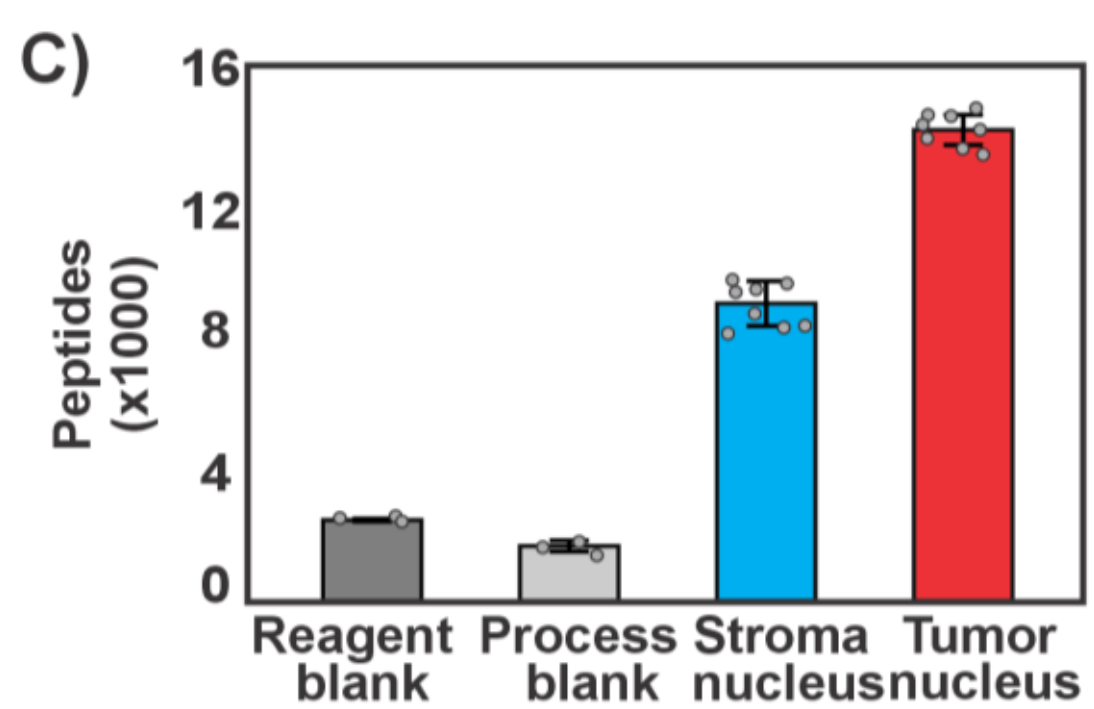

(Supplementary Fig. 8, now revised as Fig. 4). Benchmark of subcellular-resolution spatial proteomics in nuclear-enriched microregions. (C) Bar plot showing peptide identification counts from nuclear-enriched stromal and tumor regions (~60 µm²), as well as reagent and process blanks. **(D)** Same as **(C)** but showing the protein group counts.

Reviewer #3 (Remarks to the Author):

The authors have substantially improved the manuscript, and I especially appreciate the addition of process and reagent blank controls for the single-nucleus measurements, which directly address my concern that the reported identifications could partly reflect background. The clarification of the on-tip comparison is also welcome, thank you!

I have now tried the method myself but could unfortunately not entirely reproduce the authors' findings. In particular, I found dramatically varying buffer volumes in the Evochip wells after the hanging-droplet incubation. A few parameters of this step were unclear to me, and for the method to be useful I would ask the authors to expand the description of the decrosslinking incubation step, which is currently described in only about three lines (ll. 547–549).

We thank the reviewer for their positive assessment of the revised manuscript and for independently testing the method. We also greatly appreciate the constructive feedback regarding reproducibility.

Regarding the variability in buffer volumes observed after the hanging-droplet incubation, we agree that this step is critical for robust implementation. This effect is most likely related to insufficient sealing between the ProteoCHIP EVO 96and the 96-well plate during incubation. We find that this can be improved by pre-conditioning the plate (e.g., cooling the 96-well plate prior to assembly), which promotes slight thermal contraction and thereby enhances sealing with the ProteoCHIP EVO 96. These practical recommendations have now been added in more detail to the Methods section.

Accordingly, we have substantially expanded the description of the decrosslinking incubation step in the Methods section, incorporating additional operational details and troubleshooting guidance to enhance reproducibility and facilitate workflow transfer across laboratories. Further details have been provided in Supplementary Note 1 within the Materials and Methods section. In addition, we have added a link to a video recording demonstrating each individual operation throughout the protocol to provide a visual guide and further support accurate implementation of the workflow. We believe that these revisions will provide clearer instructions and improve consistency for other groups adopting this method.

Specifically: which 96-well plate was used (manufacturer and type), and whether a lid was used during the 95 °C incubation, and if so at which temperature.

The Thermo Scientific™ Armadillo™ PCR Plates (Thermo Fisher Scientific) were used in this workflow. No lid was used for incubation

In addition, I would like to re-iterate some requests that I think should be addressed in writing:

- Beyond the incubation step above, my original concern about evaporation over a realistic collection run was not addressed. A one-hour time course was measured and collection was stated to take 20–30 min, but full-plate runs can in practice be hours to days, and the data does not cover that case. Please add this as a limitation to the discussion.

We thank the reviewer for raising this important point. We apologize for not clearly explaining the collection workflow in the previous version. In our workflow, the tissue slides are pre-scanned and all collection regions are defined prior to laser microdissection. These coordinates are then automatically imported into the LMD software, allowing fully automated sample collection into the EVO96 chip. Under these conditions, collection of a complete 96-well plate typically requires approximately 30 minutes.

Lines 519:525 reads:

“Images captured at 20X or 40X magnification using the Axioscan 7 microscope are imported into QuPath 0.5.158, where regions of interest (ROIs) are marked and annotated before being exported as a GeoJSON file. The web application “Steamlist(https://qupath-to-lmd-mdcberlin.streamlit.app/)” then converts the GeoJSON file into an XML file, which contains the coordinate information necessary for the Laser Microdissection (LMD) microscope to identify the samples”

Following protein digestion, the resulting peptides are immediately loaded onto EvoTips, where they are retained on the embedded C18 material. Because the peptides are immobilized on the C18 resin and the EvoTips are stored immersed in water until LC-MS analysis, evaporation is no longer a concern at this stage, and the samples remain stable for extended periods up to weeks.

To avoid confusion, we have clarified the automated collection workflow in the revised manuscript. Given that the collection process is completed within approximately 30 minutes and that peptide storage on EvoTips eliminates concerns regarding evaporation after digestion, we do not believe that long-term evaporation during sample collection represents a limitation of the current workflow.

- The response describes the 0.13 µL/min rate as measured at decrosslinking conditions, whereas the manuscript text states standard laboratory conditions. Please clarify, since collection occurs at room temperature with the droplet exposed. And the manuscript states that DDM compromises droplet stability (Supplementary Fig. 1F shows reduced remaining volume with DDM), whereas the response to my comment states that DDM reduces evaporation. Please make these consistent.

We apologize for the confusion and appreciate the reviewer's careful reading. To clarify, the evaporation rate of 0.13 µL/min was measured under lab standard conditions (25 °C lab temperature, and about 30°C due to the laser)

Regarding DDM, we also acknowledge the inconsistency in the description. As shown in Supplementary Fig. 1F, the remaining droplet volume after exposure to decrosslinking conditions (90 °C) is reduced in the presence of 0.005% DDM, indicating an increased evaporation rate under these conditions. This is consistent with a DDM-dependent modulation of surface properties as it significantly lowers water's surface tension leading to decreased droplet stability at elevated temperature and higher evaporation. We have revised the text to ensure that the description of DDM effects is consistent throughout the manuscript and aligned with the presented data.

- The clarification that biological replicates correspond to independent tissue regions means that the subtype comparisons rest on a single block per subtype. The authors note the single-patient design (ll. 443–446) but still frame the cross-subtype differences as biologically meaningful. Please add a clear statement that, because each subtype comes from a single sample, the differences shown between subtypes cannot be generalized and may reflect patient-to-patient variation rather than true subtype differences.

We thank the reviewer for this important clarification. We agree that, given the study design in which each cancer subtype is represented by a single patient sample, the observed cross-subtype differences cannot be generalized to define subtype-specific biology. We have therefore tempered the interpretation throughout the manuscript to avoid overstatement of cross-subtype conclusions, while maintaining that the data demonstrate the feasibility of capturing spatially resolved proteomic differences within tissue regions (lines 443:448).

"Although these experiments were conducted using samples from a single patient per cancer subtype, they demonstrate the applicability of our method by capturing biologically meaningful differences both between tumor and stromal regions and across cancer subtypes. However, we acknowledge that these observations cannot yet be generalized and may in part reflect inter-patient variability rather than true subtype-specific differences".

Reviewer #4 (Remarks to the Author):

I co-reviewed this manuscript with one of the reviewers who provided the listed reports. This is part of the Nature Communications initiative to facilitate training in peer review and to provide appropriate recognition for Early Career Researchers who co-review manuscripts.

We sincerely thank the Early Career Researcher who participated in the co-review for the careful assessment of our manuscript. We greatly appreciate the time and constructive feedback, which have helped us improve the quality of our work.

POINT-BY-POINT REBUTTAL TO REVIEWER COMMENTS (Third Revision)

High-Throughput Spatial Proteomics at Cellular and Subcellular Resolution via a Hanging Droplet Workflow

Authored by Hao C*, Guzman U.H.* & Olsen J.V.

We thank the Reviewers for their careful assessment of our manuscript in this final round of review. We appreciate their constructive comments, which have helped us further improve the manuscript.

Below, we provide a point-by-point response to all comments. All changes made to the manuscript are indicated in blue text.

Reviewer #1 (Remarks to the Author):

The authors have done a great job in improving the reproducibility and accessibility of the method, particularly by providing the detailed Supplementary Note 1 and the accompanying video tutorial. These additions significantly enhance the usability of the workflow for the community.

One suggestion is that the authors may consider providing the tutorial videos as supplementary video files associated with the manuscript, rather than relying solely on external platforms such as YouTube, to ensure more stable accessibility and long-term availability.

We thank the Reviewer for this positive assessment and for the helpful suggestion. We agree that providing the tutorial videos as supplementary files would improve their accessibility and long-term availability. Accordingly, we have included the tutorial videos as supplementary video files associated with the manuscript.

Reviewer #2 (Remarks to the Author):

In this revised manuscript, the authors have addressed my primary concerns about scalability. I request that the authors include the plots (two 96-plate datasets) in the supporting information; it is direct evidence for the claim of scalability.

Although the overall novelty is not excellent, the capability to analyze large-scale samples is quite valuable to the field.

I also appreciate the authors for including a reagent blank and prep blank in the main text to show the background of the technology.

Overall, I would recommend the publication if the above requirements are met.

We thank the Reviewer for the positive assessment of the revised manuscript and for recognizing the value of the method for large-scale sample analysis. We also thank the Reviewer for the constructive feedback and recommendation for publication.

Reviewer #3 (Remarks to the Author):

The authors have now provided a detailed account of the methods, and I find particularly supplementary note 1 including the Youtube video very helpful. Minor requests:

- Please check Fig. 7 of the supplementary note 1, which to me does not show a "representative outcome".

We have improved Fig. 7 in the Supplementary Note 1.

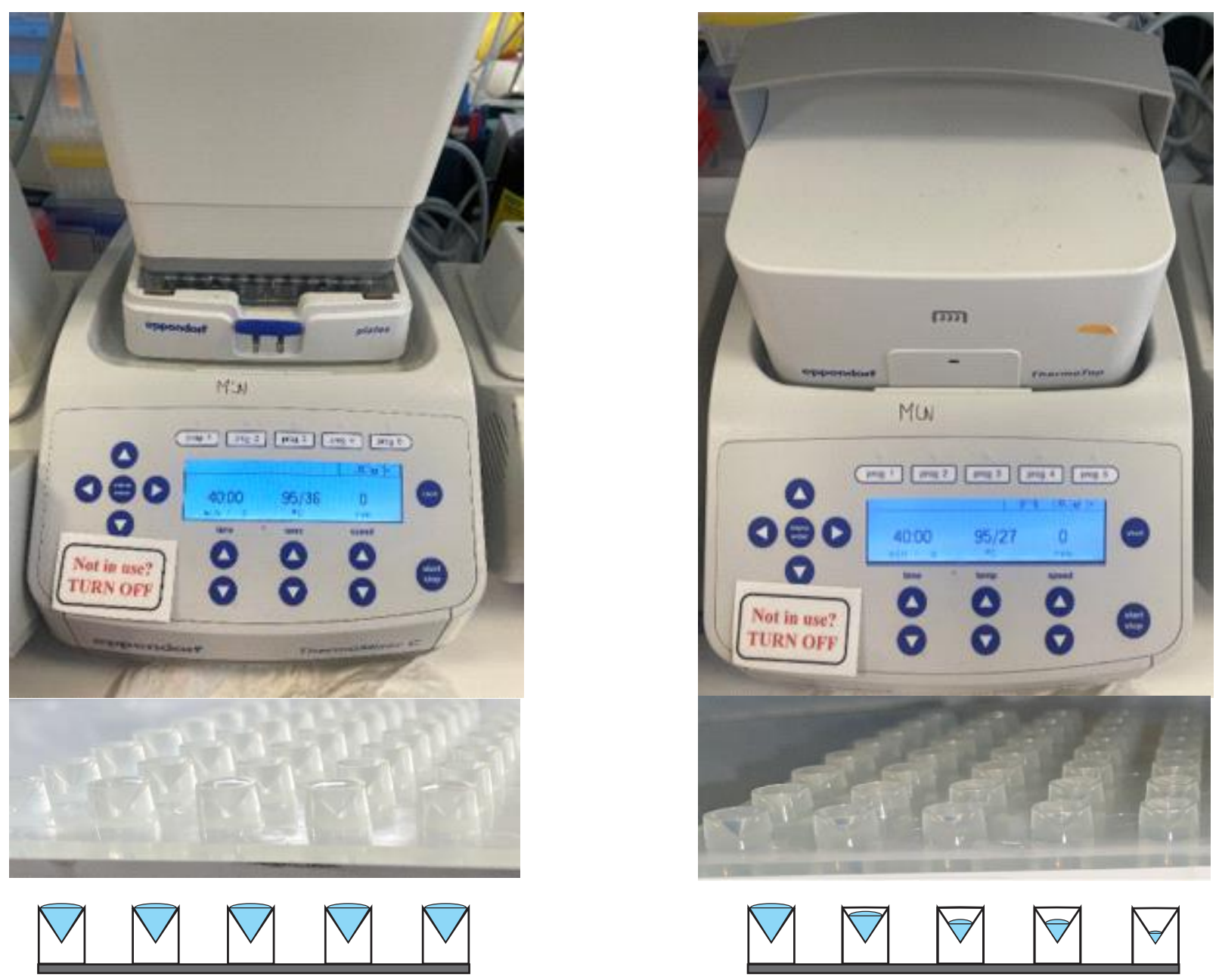

Figure 7. *Representative outcomes after decrosslinking: a correctly sealed, intact hanging droplet (left, ▯) versus a failed seal with visible droplet loss (right, ▯).*

- I am confused about the optima reported and how they are stated in the Methods and Note 1, specifically decrosslinking temperature (90°, 95 °C?), timing (1.7h [please give in minutes instead], 2.5h, 2h?), and enzyme amount (5ng or 10ng/uL).

We thank the Reviewer for highlighting this point. We have harmonized the protocol in Supplementary Note 1 and the Materials and Methods section to ensure that all reported conditions are consistent and clearly stated.

- The steps in Note 1 are off, e.g., Stage 5, steps 16-19, the section runs until step 24 (also Stage 4). Given note 1 will be extremely helpful, please revise carefully.

We thank the Reviewer for highlighting this point. We have harmonized the protocol

- "Cosias Fabian laboratory" should be "Fabian Coscia".

- The Methods section itself has not been changed extensively, but a referral to Note 1 has been added. I would welcome some key details (Armadillo plate with pre-chilling at -20 °C, removal of the heating block lid, etc.) in the methods as this is the key innovation of the paper.

- In Fig. 1B, 25mM TEAB are optimal (also stated in the main text), but then Note 1 states 10mM TEAB. Could you please harmonize?

We have harmonized the protocol

Besides these comments, I accept the replies by the authors.

We thank Reviewer #3 for the careful assessment of the revised manuscript and for the positive comments regarding the detailed methods, Supplementary Note 1, and the accompanying video tutorial. We appreciate these helpful suggestions and have carefully revised the manuscript and Supplementary Note 1 accordingly.

Reviewer #4 (Remarks to the Author):

I co-reviewed this manuscript with one of the reviewers who provided the listed reports. This is part of the Nature Communications initiative to facilitate training in peer review and to provide appropriate recognition for Early Career Researchers who co-review manuscripts.

Reviewer #5 (Remarks to the Author):

I co-reviewed this manuscript with one of the reviewers who provided the listed reports. This is part of the Nature Communications initiative to facilitate training in peer review and to provide appropriate recognition for Early Career Researchers who co-review manuscripts.

We thank Reviewers #4 and 5# for participating in the co-review of our manuscript and for their contribution to the peer-review process. We greatly appreciate the time and effort invested in the review and the constructive feedback provided.
